# TMEM106B is a receptor mediating ACE2-independent SARS-CoV-2 cell entry

**DOI:** 10.1016/j.cell.2023.06.005

**Published:** 2023-08-03

**Authors:** Jim Baggen, Maarten Jacquemyn, Leentje Persoons, Els Vanstreels, Valerie E. Pye, Antoni G. Wrobel, Valeria Calvaresi, Stephen R. Martin, Chloë Roustan, Nora B. Cronin, Eamonn Reading, Hendrik Jan Thibaut, Thomas Vercruysse, Piet Maes, Frederik De Smet, Angie Yee, Toey Nivitchanyong, Marina Roell, Natalia Franco-Hernandez, Herve Rhinn, Alusha Andre Mamchak, Maxime Ah Young-Chapon, Eric Brown, Peter Cherepanov, Dirk Daelemans

**Affiliations:** 1 KU Leuven Department of Microbiology, Immunology and Transplantation, Laboratory of Virology and Chemotherapy, Rega Institute, Leuven 3000, Belgium; 2 Chromatin Structure and Mobile DNA Laboratory, Francis Crick Institute, London NW1 1AT, UK; 3 Structural Biology of Disease Processes Laboratory, Francis Crick Institute, London NW1 1AT, UK; 4 Department of Chemistry, Britannia House, 7 Trinity Street, King's College London, London SE1 1DB, UK; 5 Structural Biology Science Technology Platform, Francis Crick Institute, London NW1 1AT, UK; 6 LonCEM Facility, Francis Crick Institute, London NW1 1AT, UK; 7 KU Leuven Department of Microbiology, Immunology and Transplantation, Laboratory of Virology and Chemotherapy, Translational Platform Virology and Chemotherapy, Rega Institute, Leuven 3000, Belgium; 8 KU Leuven Department of Microbiology, Immunology and Transplantation, Laboratory of Clinical and Epidemiological Virology, Rega Institute, Leuven 3000, Belgium; 9 KU Leuven Department of Imaging and Pathology, Laboratory for Precision Cancer Medicine, Translational Cell and Tissue Research Unit, Leuven 3000, Belgium; 10 Alector LLC, 131 Oyster Point Blvd. Suite 600, South San Francisco, CA 94080, USA; 11 Department of Infectious Disease, Section of Virology, Imperial College London, St Mary’s Campus, Norfolk Place, London W2 1PG, UK

**Keywords:** SARS-CoV-2, entry receptor, TMEM106B, coronavirus, ACE2-independent entry, antibody neutralization, cryo-EM, TMEM106B crystal structure

## Abstract

SARS-CoV-2 is associated with broad tissue tropism, a characteristic often determined by the availability of entry receptors on host cells. Here, we show that TMEM106B, a lysosomal transmembrane protein, can serve as an alternative receptor for SARS-CoV-2 entry into angiotensin-converting enzyme 2 (ACE2)-negative cells. Spike substitution E484D increased TMEM106B binding, thereby enhancing TMEM106B-mediated entry. TMEM106B-specific monoclonal antibodies blocked SARS-CoV-2 infection, demonstrating a role of TMEM106B in viral entry. Using X-ray crystallography, cryogenic electron microscopy (cryo-EM), and hydrogen-deuterium exchange mass spectrometry (HDX-MS), we show that the luminal domain (LD) of TMEM106B engages the receptor-binding motif of SARS-CoV-2 spike. Finally, we show that TMEM106B promotes spike-mediated syncytium formation, suggesting a role of TMEM106B in viral fusion. Together, our findings identify an ACE2-independent SARS-CoV-2 infection mechanism that involves cooperative interactions with the receptors heparan sulfate and TMEM106B.

## Highlights

• TMEM106B directly engages the receptor-binding domain of SARS-CoV-2 spike
• Substitution E484D increases TMEM106B binding, enhancing TMEM106B-mediated entry
• TMEM106B-specific antibodies neutralize SARS-CoV-2 infection
• TMEM106B promotes spike-mediated syncytium formation

## Graphical abstract

**Figure:**
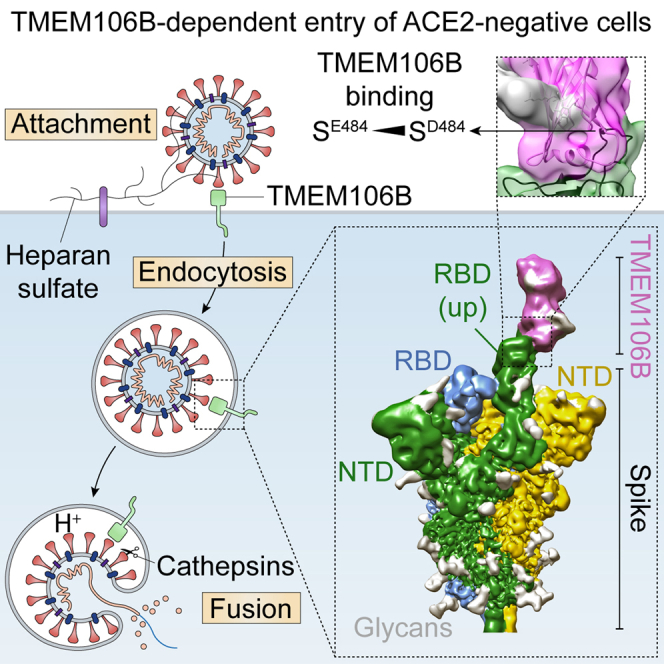

## Teaser

The lysosomal transmembrane protein TMEM106B can serve as an alternative receptor for SARS-CoV-2 entry into ACE2-negative cells. Spike substitution E484D improves spike binding to TMEM106B, enhancing TMEM106B-mediated SARS-CoV-2 infection.

## Introduction

The COVID-19 pandemic prompted unprecedented global collaboration to investigate coronavirus biology and initiated numerous clinical trials to identify vaccines and antiviral drugs against SARS-CoV-2 infection.^1^ This rapidly led to the discovery that angiotensin-converting enzyme 2 (ACE2), previously known as the main receptor for SARS-CoV-1,^2^ mediates the cell entry of SARS-CoV-2.^3^^,^^4^^,^^5^ Virus entry was found to also depend on transmembrane protease serine 2 (TMPRSS2) or endo/lysosomal cathepsins.^5^^,^^6^ Efforts to identify antiviral drug targets have focused on virus-encoded factors as well as host-encoded proviral factors. The latter strategy is thought to reduce the probability of resistance development and result in drugs with broad-spectrum activity.^7^^,^^8^ To identify such potentially druggable host factors, we and others have recently reported CRISPR-based genome-wide knockout screens to uncover genes involved in SARS-CoV-2 infection. Several of these screens, including ours, identified TMEM106B as a proviral host factor.^9^^,^^10^^,^^11^ Furthermore, *TMEM106B* was identified in a genome-wide CRISPR-based activation screen, suggesting that *TMEM106B* overexpression promotes SARS-CoV-2 infection.^12^ We and others demonstrated that *TMEM106B* is critical for the SARS-CoV-2 infection of several cell lines, whereas it is dispensable for HCoV-229E or HCoV-OC43.^9^^,^^13^ We showed that *TMEM106B* overexpression enhanced infection by pseudoviruses carrying SARS-CoV-2 spike. However, the mechanism by which TMEM106B promotes SARS-CoV-2 infection remained elusive. As a type II transmembrane protein, comprising 274 amino acid residues, TMEM106B localizes to late endosomes and lysosomes.^14^^,^^15^^,^^16^ It is expressed in a large variety of cell types, with highest levels in the brain, heart, thyroid, adrenal, and testis tissues (www.proteinatlas.org).^17^ TMEM106B is associated with brain aging; myelination disorders; and several neurodegenerative diseases, including frontotemporal lobar degeneration (FTLD), amyotrophic lateral sclerosis (ALS), Alzheimer’s disease, and Parkinson’s disease.^18^ Multiple single-nucleotide polymorphisms in *TMEM106B* have been linked to the severity of these disorders,^18^ with an association between risk alleles and increased TMEM106B expression.^19^ Recently, three independent studies reported the presence of amyloid fibrils consisting of a TMEM106B C-terminal fragment in the brains of patients with Aβ-amyloidoses, tauopathies, synucleinopathies, and TDP-43 proteinopathies.^20^^,^^21^^,^^22^ Because TMEM106B fibrils were also found in the frontal cortices of individuals without neurological disease,^21^ it remains to be determined whether these fibrils play a role in disease etiology. In addition, TMEM106B was identified as a driver of lung cancer metastasis.^23^ TMEM106B consists of an N-terminal cytosolic domain, a transmembrane helix, and a glycosylated C-terminal luminal domain (LD) that can be shed upon cleavage by lysosomal proteases.^18^ It forms homodimers as well as heterodimers with its homolog TMEM106C^24^ and possibly also with TMEM106A. TMEM106B plays a role in controlling the size and motility of lysosomes,^15^^,^^24^^,^^25^^,^^26^ but the molecular mechanism behind this function remains unknown. Several lysosomal proteins, including V-type proton ATPase subunit S1^27^ and the protease cathepsin D,^28^ bind TMEM106B. Based on sequence similarity with two yeast proteins, TMEM106B was recently proposed to be a lipid transfer protein,^29^ which is pending experimental verification.

Although ACE2 is the best characterized SARS-CoV-2 receptor, several studies reported that SARS-CoV-2 can also infect cell lines that lack detectable ACE2 expression.^30^^,^^31^^,^^32^ The ability to infect ACE2-negative cells was associated with substitution E484D in SARS-CoV-2 spike,^30^^,^^31^ but the underlying mechanism remained unknown. Here, we reveal that TMEM106B is a SARS-CoV-2 receptor that directly engages the receptor-binding domain (RBD) of spike and show that substitution E484D enhances the infection of ACE2-negative cells by increasing TMEM106B binding.

## Results

### Different SARS-CoV-2 isolates can use TMEM106B for infection

**Figure S1:**
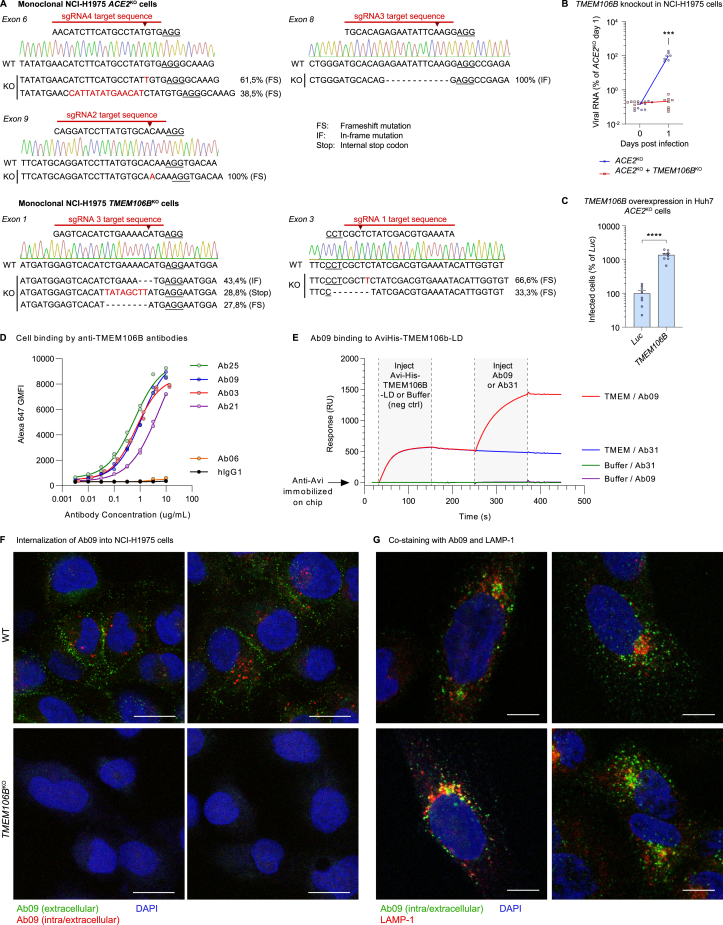
TMEM106B supports SARS-CoV-2 infection, and TMEM106B-specific monoclonal antibodies are internalized into cells, related to Figures 1 and 2 (A) Confirmation of *TMEM106B* and *ACE2* knockout in monoclonal NCI-H1975 cell lines generated by CRISPR-Cas9. For each sgRNA, the target sequence is shown. The cut site is indicated by an arrowhead, and the protospacer adjacent motif (PAM) is underlined. Wild-type (WT) sequences of the corresponding exons were determined by Sanger sequencing and are presented with chromatograms. Sequences of knockout cells were determined by next-generation sequencing. For each detected sequence variant, the detection frequency and the type of mutation are shown. (B) NCI-H1975 cells expressing sgRNAs targeting *ACE2* (monoclonal) or both *ACE2* and *TMEM106B* and infected with SARS-CoV-2 Belgium/GHB-03021/2020. Viral RNA in cells was measured by qPCR at the indicated time points (n = 8 wells examined over two independent experiments.). p values for differences between *ACE2*^KO^ and *ACE2*^KO^/*TMEM106B*^KO^ on day 1 were calculated using Mann-Whitney test with Holm-Šídák correction for multiple comparisons. ^∗∗∗^0.0001 < p < 0.001. (C) Huh7 cells transduced with sgRNAs targeting *ACE2* and with cDNA encoding luciferase (*Luc*) or *TMEM106B* and infected with SARS-CoV-2 Belgium/GHB-03021/2020. Cells were stained for nucleocapsid after 48 h. Infected cells were quantified by high-content imaging analysis (n = 8 wells examined over two independent experiments). Data were analyzed using two-sided unpaired t test with Welch’s correction. Data are mean ± SEM. ^∗∗∗∗^p < 0.0001. (D) Binding of TMEM106B-specific monoclonal antibodies to A549 cells stably overexpressing *TMEM106B*. Cells were incubated with antibody at the indicated concentrations and stained with Alexa Fluor 647-labeled anti-human IgG. The geometric mean fluorescence intensity (GMFI) compared with an hIgG1 isotype control is shown. (E) Binding of Ab09 to recombinant AviHis-TMEM106B-luminal domain (LD) was confirmed by surface plasmon resonance. Ab09 bound to recombinant AviHis-TMEM-LD that was captured by immobilized rabbit anti-Avi antibody. This confirmed that the epitope bound by Ab09 was intact on the recombinant protein. (F) WT and *TMEM106B*^KO^ NCI-H1975 cells incubated with Ab09 for 2 h at ambient temperature to assess antibody internalization. Extracellular Ab09 was stained on live cells (green), followed by fixation, permeabilization, and staining of both extracellular and intracellular Ab09 (red) and nuclei (blue). The presence of red foci in WT cells indicates the endocytic uptake of anti-TMEM106B. Scale bars, 20 μm. (G) NCI-H1975 cells incubated with Ab09 for 50 min at ambient temperature and stained for Ab09 (green), LAMP-1 (red), and nuclei (blue). Four representative images are shown. Scale bars, 10 μm.

**Figure 1:**
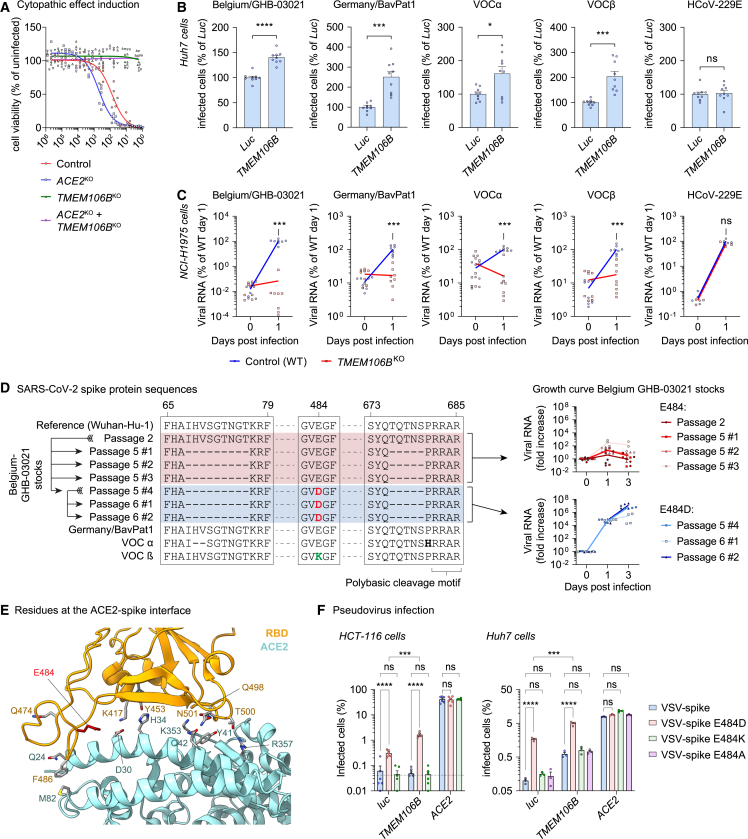
Different SARS-CoV-2 isolates can employ TMEM106B for infection, and spike substitution E484D enhances TMEM106B usage (A) NCI-H1975 cells expressing sgRNAs targeting *ACE2* (monoclonal) or *TMEM106B* (polyclonal) infected with SARS-CoV-2 Belgium/GHB-03021/2020. Cell viability was determined by MTS assay after 4 days (n = 6 wells from two experiments). Fit curves were calculated by least squares regression. (B) Huh7 cells transduced with luciferase (*Luc*) or *TMEM106B* cDNA and infected with SARS-CoV-2 isolates or HCoV-229E. Cells were stained for nucleocapsid (SARS-CoV-2) after 24 h or double-stranded RNA (dsRNA) (HCoV-229E) after 48 h (n = 9 wells from three experiments). Data were analyzed using two-sided unpaired t test with Welch’s correction. (C) NCI-H1975 wild-type (WT) or monoclonal *TMEM106B*^KO^ cells infected with SARS-CoV-2 isolates at multiplicity of infection (MOI) 10 or HCoV-229E at MOI 2. Viral RNA in cells was measured by qPCR (SARS-CoV-2: n = 8 wells from three experiments; HCoV-229E: n = 4 wells from one experiment). p values for differences between WT and TMEM106B^KO^ on day 1 were calculated using Mann-Whitney test with Holm-Šídák correction for multiple comparisons. (D) Left: alignment of spike protein sequences of SARS-CoV-2 stocks used in this study. Right: NCI-H1975 cells infected with different stocks of SARS-CoV-2 Belgium/GHB-03021/2020 at MOI 1. Viral RNA in cells was measured by qPCR (n = 4 wells from two experiments). (E) Close-up view of the interactions between ACE2 (cyan) and the SARS-CoV-2-receptor-binding domain (RBD; orange), with Glu484 in red. Side chains are shown for important residues at the ACE2-spike interface.^3^ (F) HCT-116 or Huh7 cells transduced with luciferase (*luc*), *TMEM106B*, or *ACE2* cDNA and infected with pseudoparticles harboring SARS-CoV-2 spike (VSV-spike; sequence of isolate Belgium/GHB-03021 passage 6) containing the indicated substitutions. GFP expression was quantified 24 h post-infection (n = 6 wells [HCT-116] from two experiments or n = 3 wells [Huh7] from one of two experiments with similar results). Data were log-transformed and analyzed using two-way ANOVA with Tukey’s multiple comparison test. (B and F) Data are mean ± SEM. ^∗∗∗∗^p < 0.0001; ^∗∗∗^0.0001 < p < 0.001; ^∗^0.01 < p < 0.05; ns, not significant. See also Figure S1.

We previously reported that TMEM106B is essential for the infection of several human cell lines that express *ACE2* at a low or an undetectable level,^9^ including Huh7 and lung-derived NCI-H1975 cells. To confirm that *TMEM106B* can support ACE2-independent infection, we generated *ACE2* and *TMEM106B* knockout NCI-H1975 cells (*ACE2*^KO^ and *TMEM106B*^KO^) (Figure S1A). As expected, *ACE2* knockout did not prevent cytopathic effect (CPE) induction (Figure 1A) or viral RNA production (Figure S1B) by SARS-CoV-2, whereas *TMEM106B* knockout completely abolished infection. Moreover, *TMEM106B* overexpression in Huh7 *ACE2*^KO^ cells stimulated infection (Figure S1C). Thus, TMEM106B can support SARS-CoV-2 infection independently of ACE2. To establish whether TMEM106B is a common proviral host factor for SARS-CoV-2, we tested whether two early SARS-CoV-2 isolates (Belgium/GHB-03021 and Germany/BavPat1) and the variants of concern (VOCs) α and β can utilize TMEM106B. *TMEM106B* overexpression in Huh7 cells enhanced infection with all isolates, whereas infection with the TMEM106B-independent coronavirus HCoV-229E remained unaffected (Figure 1B). *TMEM106B* knockout in NCI-H1975 cells reduced viral RNA production by the SARS-CoV-2 isolates, but not HCoV-229E (Figures 1C and S1A), demonstrating that different SARS-CoV-2 isolates depend on TMEM106B for infection.

### Amino acid substitution E484D in spike promotes TMEM106B-mediated SARS-CoV-2 infectivity

We noticed that in wild-type (WT) NCI-H1975 cells, viral RNA levels of isolate Belgium/GHB-03021 increased more than 1,000-fold within 1 day of infection (Figure 1C), whereas the remaining isolates replicated considerably slower. We also observed variations in the infection efficiency of our different stocks of isolate Belgium/GHB-03021 on NCI-H1975 cells (Figure 1D). Sequencing analysis revealed that amino acid substitution E484D in spike, which likely emerged during virus passaging, was responsible for increased infectivity (Figure 1D). Concordantly, two recent studies reported that E484D facilitates SARS-CoV-2 entry into ACE2-negative cell lines.^30^^,^^31^ Asp at position 484 is occasionally detected in circulating SARS-CoV-2 isolates,^31^ whereas Lys484 is found in VOCs alpha and beta, and Ala484 is present in VOC omicron.^33^ Remarkably, residue 484 is located within the receptor-binding motif of the RBD of spike, near the residues that directly interact with ACE2 (Figure 1E). Although substitution E484D might influence ACE2 binding, this cannot cause the increased infectivity, as the infection of NCI-H1975 cells is ACE2 independent (Figure 1A). Besides E484D, the Belgium/GHB-03021 isolate contains additional changes in spike acquired during passaging, including two deletions (Figure 1D). One of these deletions flanks the polybasic cleavage motif at the S1/S2 cleavage site, and similar deletions were shown to affect the mode of SARS-CoV-2 entry into cells.^34^ To confirm that E484D is responsible for enhancing ACE2-independent infection, we generated vesicular stomatitis virus (VSV) particles pseudotyped with SARS-CoV-2 spike harboring Glu, Asp, Lys, or Ala at position 484. We compared their infectivity in Huh7 and HCT-116 cells overexpressing either luciferase (*luc*) (control), *ACE2*, or *TMEM106B*. Pseudovirus carrying E484D displayed increased infectivity in cells transduced with control (*luc*) or *TMEM106B* cDNA compared with pseudovirus harboring E484, E484K, or E484A (Figure 1F). By contrast, there was no difference in infectivity between variants (Glu, Asp, Lys, or Ala) in cells overexpressing *ACE2*. In conclusion, these data show that E484D specifically enhances SARS-CoV-2 infection via TMEM106B but not via ACE2.

### TMEM106B-specific monoclonal antibodies block SARS-CoV-2 entry

**Figure 2:**
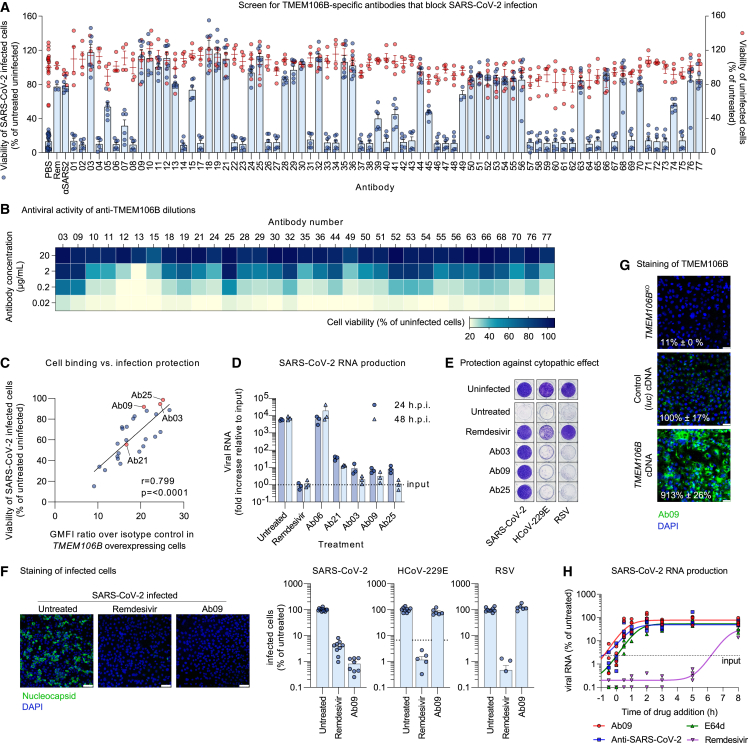
TMEM106B-specific monoclonal antibodies block SARS-CoV-2 entry (A) NCI-H1975 cells pretreated with TMEM106B-specific antibodies at 20 μg/mL and infected with SARS-CoV-2 Belgium/GHB-03021/2020. Positive controls: remdesivir (Rem) and hamster anti-SARS-CoV-2 serum (αSARS2). Cell viability was determined by MTS assay after 3 days (n = 6 wells (infected; blue) or 4 wells (uninfected; red) from two experiments). (B) Heatmap representation of the viability of NCI-H1975 cells pretreated with different concentrations of TMEM106B-specific antibodies and infected with SARS-CoV-2 Belgium/GHB-03021/2020. Cell viability was determined by MTS assay after 3 days. (C) Correlation plot showing the ability of TMEM106B-specific antibodies to neutralize SARS-CoV-2 infection of NCI-H1975 cells at 2 μg/mL (y axis) and their ability to bind A549 cells overexpressing TMEM106B. Binding is expressed as the geometric mean fluorescence intensity (GMFI) relative to a hIgG1 isotype control. r, Spearman correlation. Fit curve was calculated by linear regression. (D) NCI-H1975 cells pretreated with TMEM106B-specific antibodies or remdesivir and infected with SARS-CoV-2 Belgium/GHB-03021/2020 at MOI 10. Viral RNA in cells was measured by qPCR (n = 3 wells from one of two experiments with similar results). (E) NCI-H1975 cells pretreated with TMEM106B-specific antibodies or remdesivir and infected with SARS-CoV-2 Belgium/GHB-03021/2020, HCoV-229E, or RSV. Cells were stained with crystal violet after 4 days. (F) NCI-H1975 cells pretreated with anti-TMEM106B (Ab09) or remdesivir and infected with SARS-CoV-2 Belgium/GHB-03021/2020, HCoV-229E, or RSV. After 24 h, cells were stained for nucleocapsid (SARS-CoV-2), dsRNA (HCoV-229E), or F (RSV). Left: representative confocal images. Scale bars, 100 μm. Right: quantification (SARS-CoV-2: n = 8–12 wells from three experiments; HCoV-229E and RSV: n = 6–9 wells from two experiments). Dotted line: lower detection limit. (G) Monoclonal *TMEM106B*^KO^ or wild-type (WT) NCI-H1975 cells transduced with luciferase (*Luc*) or *TMEM106B* cDNA and stained with Ab09. Intensities were quantified using ImageJ (n = 4 wells), and representative images are shown. Scale bars, 50 μm. (H) NCI-H1975 cells treated at different time points with Ab09, anti-SARS-CoV-2, E64d, or remdesivir. At 11 h post-infection, viral RNA in cells was measured by qPCR (n = 4 wells from two experiments). Data are normalized to infected untreated cells. Fit curves were calculated by robust regression. (A, D, F, and G) Data are represented as mean ± SEM. See also Figure S1.

To establish whether TMEM106B is required for viral entry, we tested a set of 75 monoclonal antibodies raised against TMEM106B.^34^^,^^35^ NCI-H1975 cells pretreated with each antibody were infected with SARS-CoV-2, followed by cell viability measurement. Of these antibodies, 33 abrogated or reduced CPE induction by the virus, without compromising the viability of uninfected cells (Figure 2A). To further assess their potency, the active antibodies were tested at different concentrations, revealing pronounced differences between them (Figure 2B). Importantly, the neutralizing activities of the antibodies significantly correlated with their ability to bind TMEM106B-overexpressing cells (Figures 2C and S1D), suggesting that the antiviral effect depends on the affinity of the antibody for TMEM106B. To confirm that the antibodies not only prevent virus-induced CPE but also block SARS-CoV-2 infection, we measured viral RNA production in cells pretreated with the three best performing antibodies (Ab03, Ab09, and Ab25), as well as an inactive (Ab06) and a partially active (Ab21) antibody. The results were in line with the observed effects on CPE induction and showed a nearly complete block of SARS-CoV-2 infection by Ab03, Ab09, and Ab25, similar to the effect of remdesivir treatment (Figure 2D). Next, to exclude the possibility that antibodies act via non-specific binding, we tested their effect on other viruses, namely HCoV-229E and respiratory syncytial virus (RSV). Although remdesivir treatment blocked CPE induction (Figure 2E) and infection (Figure 2F) by all three viruses, treatment with Ab03, Ab09, and Ab25 inhibited only SARS-CoV-2. In addition, we confirmed the direct binding of Ab09 to TMEM106B by surface plasmon resonance (Figure S1E). Immunofluorescence staining of cells with anti-TMEM106B (Ab09) was absent in *TMEM106B*^KO^ cells and increased in *TMEM106B* overexpressing cells (Figure 2G), confirming the specificity of the antibody. Analysis of antibody uptake into cells showed that Ab09 was internalized (Figure S1F), suggesting that the neutralizing activity of anti-TMEM106B may occur on the cell surface, inside endo/lysosomes, or at both locations. The localization of Ab09 showed partial overlap with lysosome-associated membrane glycoprotein 1 (LAMP-1) (Figure S1G). Finally, we compared the timing of the antiviral action of TMEM106B antibodies and validated inhibitors that specifically target SARS-CoV-2 entry (polyclonal serum and E64d) or RNA synthesis (remdesivir) (Figure 2H). Similar to the other entry inhibitors and in contrast to remdesivir, the antiviral activity of Ab09 required early addition, confirming that blocking TMEM106B affects the entry stage of the SARS-CoV-2 life cycle.

### Crystal structure of the TMEM106B LD

**Figure S2:**
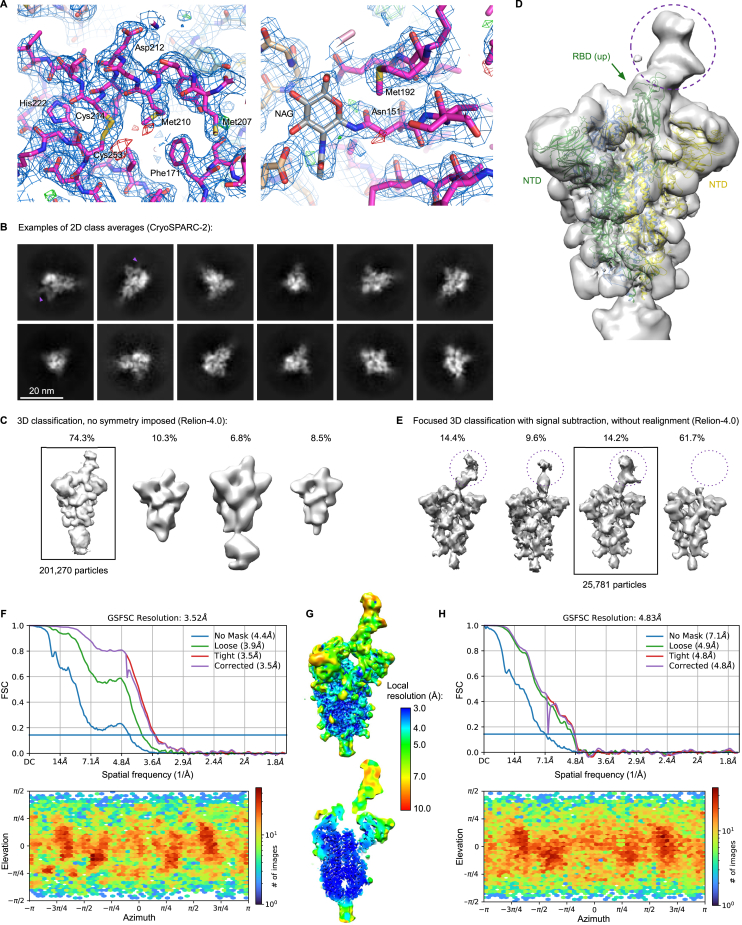
TMEM106B directly interacts with the RBD of SARS-CoV-2 spike, related to Figure 3 (A) TMEM106B crystal structure is shown as sticks with *2Fo-Fc* electron density in blue mesh (contoured at 1 RMSD) and positive and negative *Fo-Fc* density in green and red mesh (contoured at 3 RMSD), respectively. The regions shown correspond to α1 helix (residues 208–216), left, and glycosylated Asn151, right. Carbon atoms of TMEM106B amino acid residues are colored magenta (chain A) or pink (molecules related by crystal symmetry). Carbon atoms of NAG residues are shown in gray. Other atoms are colored according to the standard format: oxygen, red; nitrogen, blue; and sulfur, yellow. (B) Examples of 2D class averages of the trimeric spike ectodomain. TMEM106B^LD^ is visible in some 2D class average (purple arrowheads). (C) Result of the classification of the spike particles into four 3D classes. The class containing 201,270 particles selected for further processing is boxed. (D) Unmasked 3D reconstruction using particles images selected after initial 3D classification (B). The cryo-EM map is shown as a semi-transparent surface with the feature corresponding to associated TMEM106B indicated with dotted purple circle. Fitted is an atomistic model of the spike trimer in 1RBD-up conformation (PDB: 7NTA); RBD and NTD domains are indicated. (E) Results of focused 3D classification after signal subtraction, as detailed in the STAR Methods section. The displayed cryo-EM reconstructions were obtained after reversion to the original (non-subtracted) particles. One class selected for the final reconstruction is boxed. (F) Resolution and particle orientation metrics for final cryo-EM reconstructions. Half-map Fourier shell correlations (FSCs) and distribution of the refined particle orientations for the result of the final global non-uniform refinement, as implemented in cryoSPARC. (G) The final 3D reconstruction, colored by local resolution. (H) Half-map FSCs and particle orientation for local refinement using a soft mask covering TMEM106B^LD^ and the associated RBD.

**Figure 3:**
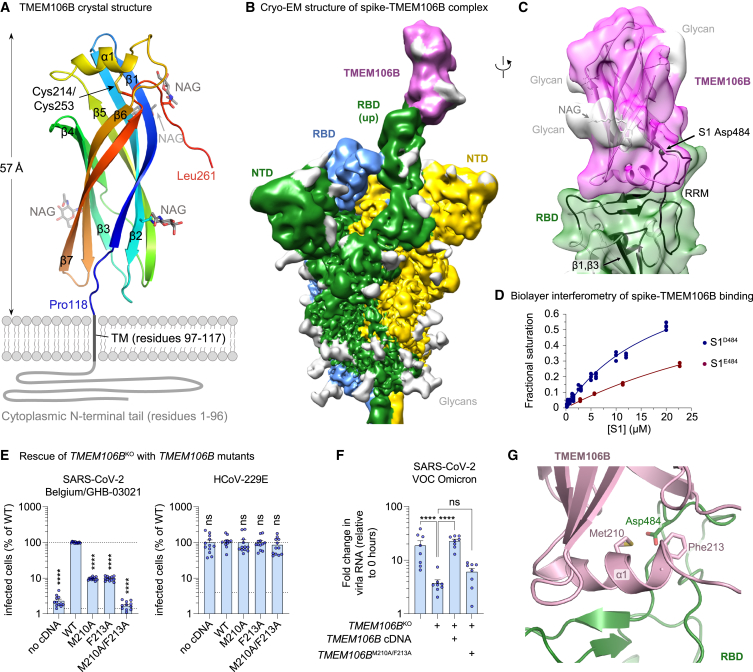
TMEM106B directly interacts with the RBD of SARS-CoV-2 spike (A) The crystal structure spanning residues 118–261 of human TMEM106B, shown as cartoons, colored by the rainbow gradient from N (blue) to C (red) terminus. The remainder of the protein, comprising the transmembrane region (TM, residues 97–117) and cytoplasmic tail (residues 1–96), is schematically represented as thick gray lines. Secondary structure elements (α1, β1–β7), N-acetylglucosamine (NAG) residues, Cys214–Cys253 disulfide, the TM, and the cytoplasmic tail are indicated. NAG residues are shown as sticks with carbon atoms in gray. (B) Cryo-EM map of the spike trimer in complex with TMEM106B^LD^. Protein chains are colored by protomer: subunits of the spike trimer in green, yellow, and blue and TMEM106B in magenta. The cryo-EM map features corresponding to glycans are light gray. (C) Local reconstruction of TMEM106B^LD^ bound to the erect RBD within the spike trimer. The cryo-EM map is shown as a semi-transparent surface, colored as in (B). The atomistic models are placed by rigid body docking and shown as cartoons. The RBD regions showing protection from HDX in the presence of excess TMEM106B^LD^ are colored dark green. (D) Biolayer interferometry results of S1 binding to immobilized TMEM106B. Data are represented as plots of variation of fractional saturation with S1 concentration for the S1^E484^ (Wuhan-Hu-1; red) versus S1^D484^ (Belgium/GHB-03021; blue) spike subunits. Symbols are measured values, and solid lines are computed best fits. (E) NCI-H1975 monoclonal *TMEM106B*^KO^ cells transduced with wild-type (WT) or mutant *TMEM106B* cDNA, infected with SARS-CoV-2 Belgium/GHB-03021/2020 or HCoV-229E. Cells were stained for dsRNA after 24 h (n = 12 wells from three experiments). Data were log-transformed and analyzed using one-way ANOVA with Dunnett’s multiple comparison test, comparing each condition with WT TMEM106B. (F) NCI-H1975 cells or monoclonal *TMEM106B*^KO^ cells transduced with WT or mutant *TMEM106B* cDNA, infected with SARS-CoV-2 VOC omicron. Viral RNA in cells was measured by qPCR at 0 and 24 h post-infection (n = 8 wells from two experiments). Data were log-transformed and analyzed using one-way ANOVA with Tukey’s multiple comparison test. (G) Close-up view of the spike-TMEM106B interface shown in (C). TMEM106B and the RBD are shown as purple and green cartoons with side chains of TMEM106B Met210 and Phe213 and spike Asp484 as sticks. Consistent with binding data (D and Figure S3F), the model predicts that the three residues project into the protein-protein interface. (E and F) Data are mean ± SEM. ^∗∗∗∗^p < 0.0001; ns, not significant. See also Figures S2, S3, and S4.

To enable biochemical and structural studies, we produced the luminal portion of human TMEM106B (residues 118–274, designated TMEM106B^LD^) by overexpression in human cells. The protein crystallized following partial deglycosylation by endoglycosidase H, and the structure was refined to 2.6 Å resolution (Figure S2A; Table S1). The final model spanning TMEM106B residues 118–261 revealed a compact fibronectin type III (Fn3) domain (Figure 3A), an ubiquitous 7-bladed β sandwich fold, closely related to immunoglobulin domains.^35^ In TMEM106B, the domain is crowned by a short α helix (α1, residues 208–216), which is inserted into a loop between the canonical Fn3 β strands 5 and 6 and stabilized by a disulfide bond between Cys214 and Cys253. Endoglycosidase H cleaves N-linked glycans within the chitobiose core, leaving N-acetyl-D-glucosamine (NAG) attached to the protein. Concordantly, four NAG residues were found in the structure, linked to TMEM106B Asn residues 145, 151, 164, and 256 (Figure 3A). The N-terminal portion of the protein, not present in the crystallized construct, comprises a cytoplasmic tail (residues 1–96) and a single-pass transmembrane region (residues 97–117). The tip of the TMEM106B LD is predicted to project by ∼60 Å from the endosomal membrane (Figure 3A).

### SARS-CoV-2 spike RBD directly interacts with TMEM106B

To test whether TMEM106B directly binds spike, we produced the ectodomain of SARS-CoV-2 Belgium/GHB-03021 spike, which carries Asp484, stabilized in the trimeric prefusion state by 6 proline substitutions (HexaPro^36^). We imaged the single particles of the spike in the presence of 10-fold molar excess of TMEM106B^LD^ using cryogenic electron microscopy (cryo-EM). Focused 3D classification revealed that ∼40% of the observed spike trimers displayed features consistent with one of the RBDs engaged with a small protein (Figures S2B–S2D). One well-defined 3D class comprising 25,781 particles (Figure S2E) resulted in the reconstruction of spike in 1RBD-up conformation with an extra density interpretable as a single molecule of glycosylated TMEM106B^LD^ bound to the erect RBD (Figures 3B and 3C). Although the global resolution of the map was 3.5 Å (Figures S2F and S2G), resolution of the map region defining the TMEM106B^LD^ position was ∼7 Å (Figure S2H), likely due to conformational flexibility of the local structure. Focused refinement with a mask around the RBD-TMEM106B^LD^ module improved the quality of the reconstruction (Figures 3C and S2H). Importantly, rigid body docking of SARS-CoV-2 spike placed RBD residue 484 within the TMEM106B-binding interface (Figure 3C).

**Figure S3:**
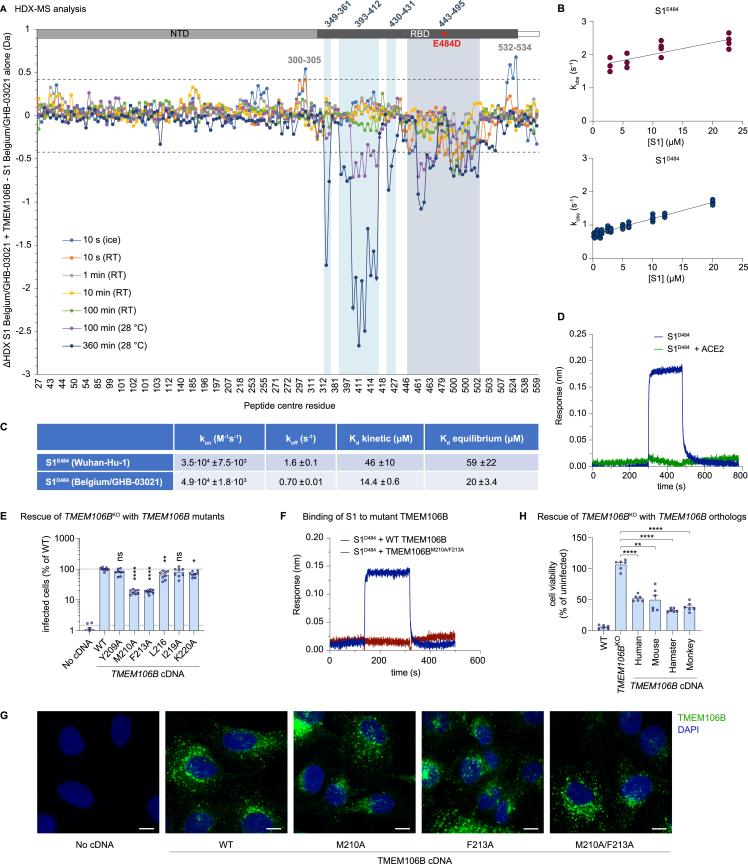
Spike residue D484 and TMEM106B residues M210 and F213 enhance spike-TMEM106B binding, related to Figure 3 (A) Differences in hydrogen-deuterium exchange (ΔHDX) between the spike S1 subunit of the Belgium/GHB-03021 isolate alone and when in the presence of excess TMEM106B. Negative values indicate protection and positive values deprotection from exchange in the presence of TMEM106B. The threshold of significance calculated with 98% CI at ±0.42 Da is indicated with a dashed gray line. Peptides are arranged from the N to C terminus according to their peptide center residue. See Table S3 and Data S1 for HDX data tables and deuterium uptake plots. (B) Biolayer interferometry results of S1 binding to immobilized TMEM106B. Data are represented as the dependence of the observed rate constant on S1 concentration for S1^E484^ (Wuhan-Hu-1) and S1^D484^ (Belgium/GHB-03021). (C) Thermodynamic parameters for S1^E484^ and S1^D484^ subunit binding to immobilized TMEM106B. For each variant, k_on_ was determined from the slope of the plot of k_obs_ against (S1) for the association phase, k_off_ was obtained from the intercept of the plot of k_obs_ against (S1) for the association phase, K_D_ kinetic was calculated as k_off_/k_on_, and K_D_ equilibrium was calculated from the plot of the amplitude versus (S1). (D) Biolayer interferometry traces of 6 μM S1^D484^ (blue) and 6 μM S1^D484^ premixed with 8 μM ACE2 (green) binding to immobilized TMEM106B (luminal domain). (E) NCI-H1975 monoclonal *TMEM106B* knockout cells transduced with cDNA encoding wild-type (WT) *TMEM106B* or *TMEM106B* containing single amino acid changes, infected with SARS-CoV-2 Belgium/GHB-03021/2020. Cells were stained for dsRNA after 24 h. Infected cells were quantified by high-content imaging analysis (n = 8 wells examined over two independent experiments). Data were log-transformed and analyzed using one-way ANOVA with Dunnett’s multiple comparison test, comparing each condition with WT TMEM106B. (F) Biolayer interferometry traces of 13.5 μM S1^D484^ binding to immobilized wild-type TMEM106B (blue) or mutant TMEM106B^M210A/F213A^ (red). (G) NCI-H1975 monoclonal *TMEM106B* knockout cells transduced with cDNA encoding WT or mutant *TMEM106B* and stained with anti-TMEM106B (Ab09) and DAPI. Representative images are shown, scale bars, 10 μm. (H) WT or *TMEM106B*^KO^ NCI-H1975 cells transduced with cDNA encoding human, mouse (*mus musculus*), hamster (*Mesocricetus auratus*), or African green monkey (*Chlorocebus sabaeus*) *TMEM106B* and infected with SARS-CoV-2 Belgium/GHB-03021/2020. Cell viability was determined by MTS assay after 3 days (n = 6 wells examined over two independent experiments). Data were analyzed using one-way ANOVA with Dunnett’s multiple comparison test, comparing each condition with *TMEM106B*^KO^ cells. (E and G) Data are mean ± SEM. ^∗∗∗∗^p < 0.0001; ^∗∗^0.001 < p < 0.01; ^∗^0.01 < p < 0.05; ns, not significant.

**Figure S4:**
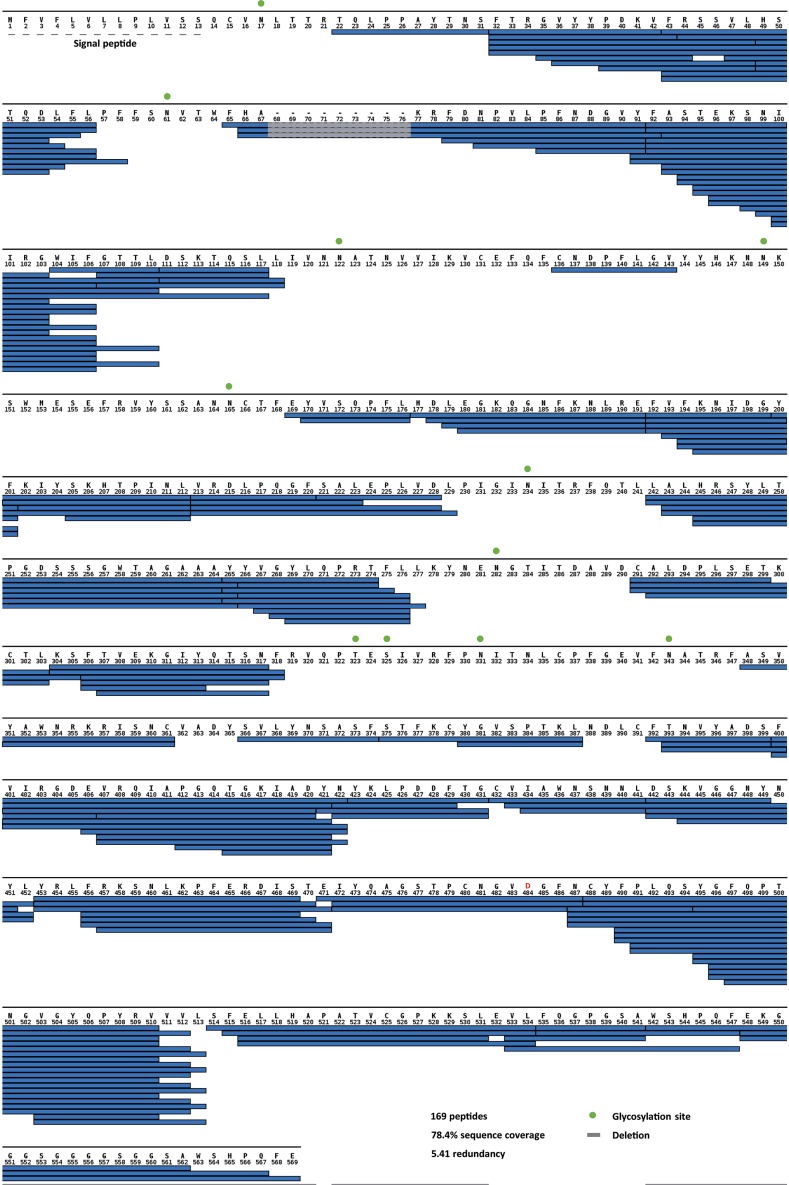
Spike sequence coverage in HDX assays, related to Figure 3 Peptides of Belgium/GHB-03021 S1^D484^ whose HDX was experimentally followed for HDX-MS analysis are indicated with blue bars. Potential sites of N- and O-linked glycosylation are indicated with green spheres above the amino acid sequence. The residue numbering is adapted to the sequence of the ancestral spike of the original Wuhan-Hu-1 isolate.

To confirm the RBD-TMEM106B interaction, we applied hydrogen-deuterium exchange mass spectrometry (HDX-MS). Incubation of monomeric Belgium/GHB-03021 S1^D484^ (residues 1–530, spanning the N-terminal domain and the RBD) in the presence of 3.5-fold molar excess TMEM106B^LD^ decreased HDX in several stretches of the RBD (residues 349–361, 393–412, 430–431, and 443–495; Figures S3A and S4). Our cryo-EM reconstruction highlighted RBD region 443–495 as the TMEM106B-binding platform, and HDX-MS data confirmed its direct engagement with the receptor. Strikingly, this region corresponds to the receptor-binding motif, which is responsible for ACE2 binding.^37^^,^^38^^,^^39^ Suppression of HDX within β1 and β3 RBD strands (residues 349–361 and 393–412) is consistent with the rigidification of the underlying structure upon the engagement of the receptor-binding motif by TMEM106B (Figure 3C). Moreover, HDX-MS data acquired on SARS-CoV-2 spike variants in the presence of ACE2 confirm the overlap of the ACE2- and TMEM106B- binding sites on the RBD.^40^

Next, we used biolayer interferometry to estimate the affinity of the spike-TMEM106B interaction. Analysis of the results in the equilibrium or kinetic regimes revealed that S1^D484^ bound TMEM106^LD^ with a dissociation constant of ∼20 or ∼14 μM, respectively, whereas S1^E484^ bound the host protein with approximately 3-fold lower affinity (Figures 3D, S3B, and S3C). Since the only difference in the RBDs of these two isolates is substitution E484D, this result demonstrates that Asp484 enhances the spike-TMEM106B interaction, explaining the observed increase in infectivity (Figure 1F). By contrast, S1^D484^ pre-incubated with the human ACE2 ectodomain did not display measurable binding to immobilized TMEM106B^LD^ (Figure S3D). This result indicates that ACE2, known to bind the RBD with at least several-hundred-fold higher affinity,^41^^,^^42^^,^^43^ competes with TMEM106B for the spike.

To further confirm that the spike-TMEM106B interaction is required for SARS-CoV-2 infection, we used our structural data to identify six putative spike-binding residues in TMEM106B, mutated each residue to alanine, and tested whether these TMEM106B mutants still supported SARS-CoV-2 infection (Figure S3E). Substitutions M210A and F213A had the most pronounced effect on virus infectivity. Combined, these two substitutions abrogated S1 binding *in vitro* (Figure S3F) and the ability of TMEM106B to support infection with SARS-CoV-2 Belgium/GHB-03021/2020 (Figure 3E) and VOC omicron (Figure 3F), without affecting correct TMEM106B expression and localization (Figure S3G). This indicates that Met210 and Phe213 are crucial for TMEM106B-mediated SARS-CoV-2 infection. Both residues are located within the α1 helix of the TMEM106B LD, predicted to project into the interface with the RBD (Figure 3G). Although TMEM106B sequence barely varies between mammalian species, some differences exist in the region near Met210 and Phe213. Despite these differences, human, mouse, hamster, and monkey *TMEM106B* all rescued SARS-CoV-2 infection of *TMEM106B*^KO^ cells to a similar extent (Figure S3H), showing that TMEM106B from these species can also support SARS-CoV-2 infection. Altogether, the above data show that TMEM106B is a SARS-CoV-2 receptor and engages RBD residues near the ACE2-binding site.

### TMEM106B is required for a post-endocytic stage of virus entry

**Figure 4:**
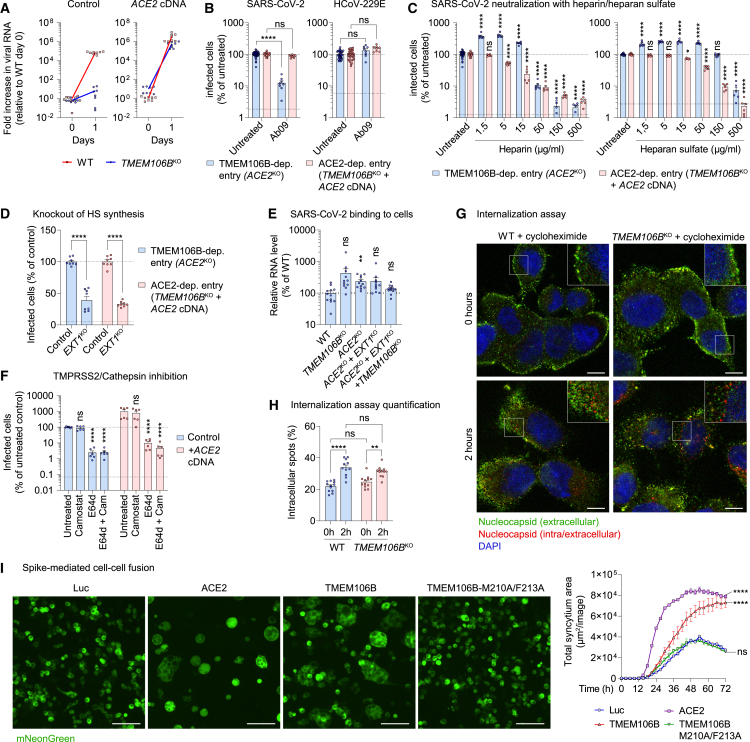
TMEM106B is required for a post-endocytic stage of virus entry (A) Wild-type (WT) or monoclonal *TMEM106B*^KO^ NCI-H1975 cells, untransduced (control) or transduced with *ACE2* cDNA and infected with SARS-CoV-2 Belgium/GHB-03021/2020 at MOI 0.03. Viral RNA in cells was measured by qPCR (n = 8 wells from two experiments). (B) Monoclonal NCI-H1975 *ACE2*^KO^ cells or *TMEM106B*^KO^ cells overexpressing *ACE2*, infected with SARS-CoV-2 Belgium/GHB-03021/2020 or HCoV-229E in the presence of anti-TMEM106B (Ab09). After 6 h (SARS-CoV-2) or 24 h (HCoV-229E), cells were stained for dsRNA (n = 8 wells from three experiments; untreated, n = 36). (C) Monoclonal NCI-H1975 *ACE2*^KO^ cells or *TMEM106B*^KO^ cells overexpressing *ACE2*, infected with SARS-CoV-2 Belgium/GHB-03021/2020 or HCoV-229E pretreated with different concentrations of heparin or heparan sulfate. After 6 h (SARS-CoV-2) or 24 h (HCoV-229E), cells were stained for dsRNA (n = 6 wells from two experiments; untreated, n = 28). (D) Monoclonal NCI-H1975 *ACE2*^KO^ cells or *TMEM106B*^KO^ cells overexpressing *ACE2*, with or without an sgRNA targeting *EXT1* (*EXT1*^KO^), infected with SARS-CoV-2 Belgium/GHB-03021/2020. After 6 h, cells were stained for dsRNA (n = 8 wells from two experiments). (E) WT NCI-H1975 cells, *ACE2*^KO^ (monoclonal), *TMEM106B*^KO^ (monoclonal), *ACE2/EXT1*^KO^ (monoclonal), or *ACE2/EXT1/TMEM106B*^KO^ (polyclonal) cells, incubated with SARS-CoV-2 Belgium/GHB-03021/2020 on ice. Viral RNA bound on cells was measured by qPCR (n = 12 wells from two experiments). Data were analyzed using one-way ANOVA with Dunnett’s multiple comparison test, comparing each condition with WT cells. (F) NCI-H1975 cells, untransduced (control) or transduced with *ACE2* cDNA, treated with E64d or camostat, infected with SARS-CoV-2 Belgium/GHB-03021/2020, and stained for nucleocapsid after 6 h (n = 6 wells from two experiments). (G) NCI-H1975 WT or monoclonal *TMEM106B*^KO^ cells, incubated with SARS-CoV-2 at MOI 8 on ice, followed by virus internalization at 35°C for 0 or 2 h in the presence of 20 μg/mL cycloheximide to block translation. Cells were stained for nucleocapsid before permeabilization (green) and after permeabilization (red) and nuclei (blue). Shown are representative images, scale bars, 10 μm. A magnification of the area in the square is shown in each upper right corner. (H) Quantified results from (G) (n = 12 wells from two experiments.). (B–D, F, and H) Upper dotted line: untreated level. Lower dotted line: detection limit. Data were log-transformed (B, C, and F) and analyzed using two-way ANOVA with Tukey’s (H), Šidák’s (B and D), or Dunnett’s (C and F) multiple comparison test, comparing each condition with the untreated control. (I) HEK293T cells co-transfected with three plasmids, encoding (1) SARS-CoV-2 Belgium/GHB-03021/2020 spike and mNeonGreen, (2) TMPRSS2, and (3) a receptor (ACE2 or TMEM106B) or control protein (Luc). Left: representative images, scale bars, 100 μm. Right: quantified syncytium area (n = 2 wells from one of two experiments with similar results). The area under the curve was calculated, followed by one-way ANOVA with Dunnett’s multiple comparison test, comparing each condition with Luc. (C, F, and I) Data are mean ± SEM. ^∗∗∗∗^p < 0.0001; ^∗∗∗^0.0001 < p < 0.001; ^∗∗^0.001 < p < 0.01; ^∗^0.01 < p < 0.05; ns, not significant. See also Figure S5.

In addition to ACE2, several alternative SARS-CoV-2 candidate receptors have been identified.^44^^,^^45^ Some of these proteins, such as neuropilin-1,^46^^,^^47^ serve as cofactors facilitating ACE2-mediated entry, whereas others support infection independently of ACE2.^45^ The observation that TMEM106B supports the infection of ACE2-negative cells (Figure 1A) suggests that TMEM106B can function as an autonomous receptor, rather than a cofactor for ACE2. To challenge this hypothesis, we assessed the effect of TMEM106B depletion on SARS-CoV-2 entry in the presence and absence of ACE2 expression. Although *TMEM106B* knockout nearly completely blocked viral RNA production in parental NCI-H1975 cells, it did not affect viral RNA production in *TMEM106B*^KO^ cells overexpressing ACE2 (Figure 4A). In line with this result, TMEM106B-specific antibody (Ab09) inhibited the infection of cells lacking ACE2 expression (*ACE2*^KO^) but did not affect the infection of cells overexpressing ACE2 (Figure 4B). These results imply that the receptors TMEM106B and ACE2 do not enable infection in a cooperative manner but support two separate modes of SARS-CoV-2 entry.

**Figure S5:**
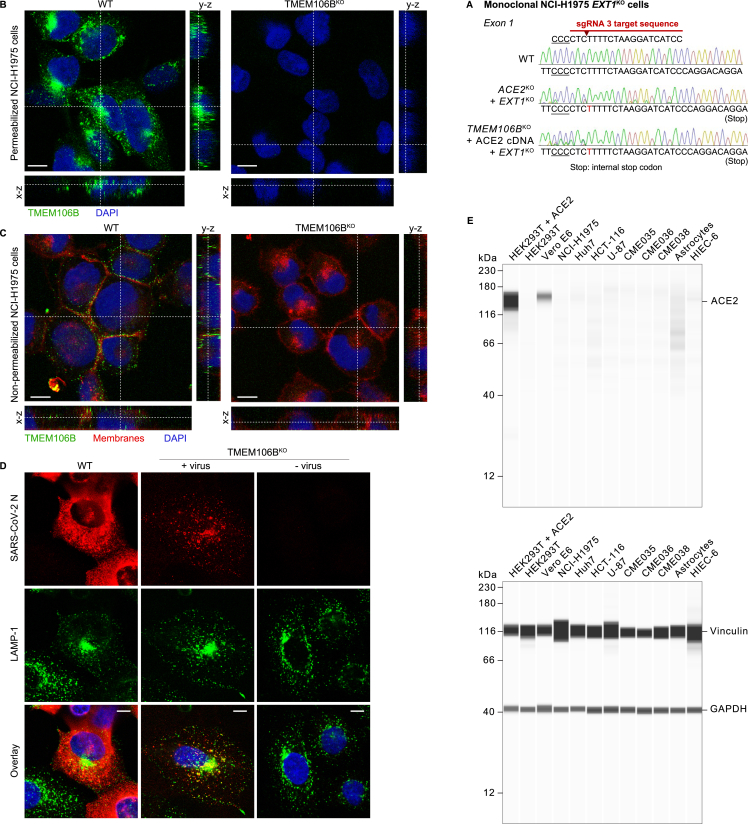
Analysis of TMEM106B cell surface expression, SARS-CoV-2 uptake into TMEM106B^KO^ cells, and ACE2 expression in various cell lines, related to Figures 4 and 5 (A) Confirmation of *EXT1* knockout in monoclonal NCI-H1975 cell lines generated by CRISPR-Cas9. The cut site within the sgRNA is indicated by an arrowhead, and the protospacer adjacent motif (PAM) is underlined. Sequences of wild-type and *EXT1* knockout cells were determined by Sanger sequencing and are shown as chromatograms. Inserted nucleotides are shown in red. (B and C) Wild-type (WT) or *TMEM106B*^KO^ NCI-H1975 cells stained for TMEM106B (Ab09; green), membranes (CellBrite Fix 640; red), and nuclei (blue). Shown are representative images from one out of two independent experiments with similar results. Cells were either permeabilized before staining (B) or not permeabilized (C) to visualize only TMEM106B expressed on the cell surface. Scale bars, 10 μm. (D) WT or *TMEM106B*^KO^ NCI-H1975 cells incubated with SARS-CoV-2 at MOI 1 for 24 h and stained for SARS-CoV-2 N (red), LAMP-1 (green), and nuclei (blue). Shown are representative images from one out of three independent experiments with similar results. Scale bars, 10 μm. Note that WT cells show more widespread N staining due to the translation of new N protein during productive infection. (E) Analysis of ACE2 expression levels in different cell lines. Lysates of the indicated wild-type cell lines or HEK293T cells transduced with an ACE2 overexpression construct were analyzed using a ProteinSimple Wes system, with antibodies specific for ACE2 and the endogenous controls vinculin and GAPDH.

We previously showed that SARS-CoV-2 infection via TMEM106B also requires the cell surface glycosaminoglycan heparan sulfate.^9^^,^^11^^,^^48^ Pretreatment of SARS-CoV-2 with heparan sulfate or the structurally similar glycosaminoglycan heparin (Figure 4C) inhibited the infection of NCI-H1975 cells via the ACE2- as well as the TMEM106B-dependent route, confirming the capacity of SARS-CoV-2 to bind glycosaminoglycans. Knockout of *EXT1* (Figure S5A), a gene essential for heparan sulfate synthesis, also inhibited SARS-CoV-2 infection via ACE2 and TMEM106B, confirming the supporting role of heparan sulfate for both infection routes (Figure 4D).

Previously, the endogenous expression of TMEM106B has only been reported in endosomes and lysosomes, but not on the plasma membrane.^14^^,^^15^^,^^16^ Immunofluorescence staining of permeabilized NCI-H1975 cells showed a predominantly intracellular localization of TMEM106B (Figure S5B), whereas staining of non-permeabilized cells revealed that a small fraction of TMEM106B also resides on the cell surface (Figure S5C). To investigate whether cell-surface-expressed TMEM106B is required for cell attachment, we performed a virus-binding assay, showing that the knockouts of *TMEM106B, ACE2*, or *EXT1* did not reduce SARS-CoV-2 binding to cells (Figure 4E). This suggests that, despite the importance of TMEM106B, ACE2, and heparan sulfate for infection, SARS-CoV-2 can still attach to the cell surface via additional factors, possibly via sialylated glycans.^49^

To investigate whether TMEM106B- and ACE2-dependent entry mechanisms involve viral endocytosis, we treated cells with E64d (a cathepsin protease inhibitor) or camostat (a serine protease inhibitor, effective against TMPRSS2) to block entry via endocytosis or via plasma membrane fusion, respectively. E64d blocked SARS-CoV-2 entry into cells with and without ACE2 overexpression (Figure 4F), indicating that the virus enters NCI-H1975 cells via the endo/lysosomal route, irrespective of ACE2 expression. This is possibly due to insufficient TMPRSS2 expression in this cell line. To investigate whether SARS-CoV-2 requires surface-expressed TMEM106B for internalization into cells, we used a sequential staining procedure to distinguish extracellular and intracellular virions. After virus binding on ice, virions were initially detected mainly on the cell surface (green), whereas after 2 h of virus uptake at 35°C, an increase in intracellular virus (red) was observed both in WT and *TMEM106B*^KO^ cells (Figures 4G and 4H). Moreover, SARS-CoV-2 inside *TMEM106B*^KO^ cells partially co-localized with the lysosomal marker LAMP-1 (Figure S5D). These data show that virus particles can still bind and undergo endocytosis into cells lacking TMEM106B.

Although SARS-CoV-2 may already engage TMEM106B on the cell surface, the above findings suggest that the crucial function of TMEM106B is promoting a post-endocytic entry step such as fusion, whereas virus attachment and endocytosis can also be mediated by other cell surface receptors. To establish whether TMEM106B can directly promote viral membrane fusion, we performed cell-cell fusion assays by co-expressing receptors with SARS-CoV-2 spike and TMPRSS2 in HEK293T cells. The co-expression of spike with ACE2 or TMEM106B induced the formation of syncytia, whereas co-expression with a control protein (Luc) or TMEM106B mutant M210A/F213A did not induce cell-cell fusion (Figure 4I). These data demonstrate that TMEM106B can facilitate spike-mediated membrane fusion.

### Cells from various organs support SARS-CoV-2 infection via TMEM106B

**Figure 5:**
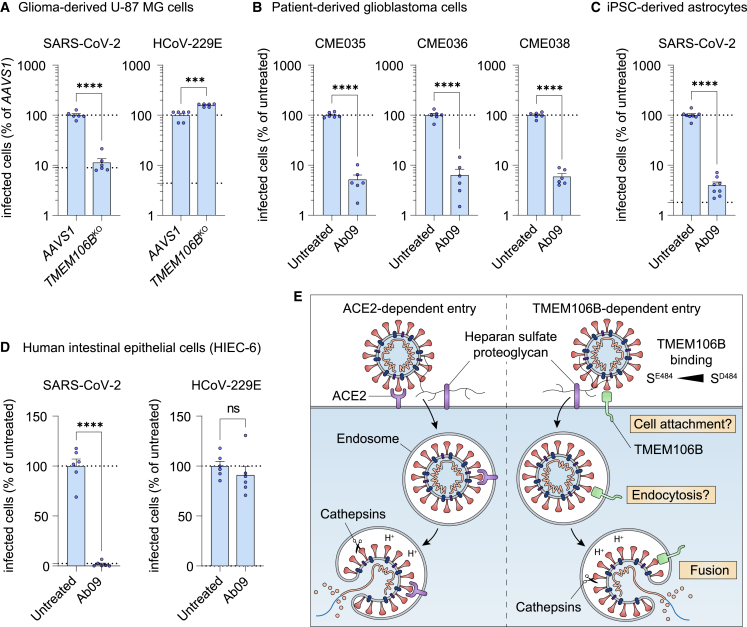
Cells from various organs support SARS-CoV-2 infection via TMEM106B (A) U-87 MG cells transduced with sgRNAs targeting *TMEM106B* or safe harbor locus *AAVS1*, infected with SARS-CoV-2 Belgium/GHB-03021/2020 or HCoV-229E, fixed after 24 (SARS-CoV-2) or 96 h (HCoV-229E). (B) Patient-derived glioblastoma cells infected with SARS-CoV-2 Belgium/GHB-03021/2020 in the presence of anti-TMEM106B (Ab09), fixed after 24 h. (C) iPSC-derived astrocytes infected with SARS-CoV-2 Belgium/GHB-03021/2020 in the presence of Ab09, fixed after 48 h. (D) HIEC-6 cells infected with SARS-CoV-2 Belgium/GHB-03021/2020 or HCoV-229E in the presence of Ab09, fixed after 96 (SARS-CoV-2) or 72 h (HCoV-229E). (A–D) Cells were stained for nucleocapsid (SARS-CoV-2) or dsRNA (HCoV-229E). Data were analyzed using two-sided unpaired t test with Welch’s correction (n = 6 wells—A, B, and D—or n = 8 wells—C —from two experiments). Upper dotted line: untreated level. Lower dotted line: detection limit. Data are mean ± SEM. ^∗∗∗∗^p < 0.0001; ^∗∗∗^0.0001 < p < 0.001; ^∗∗^0.001 < p < 0.01; ^∗^0.01 < p < 0.05; ns, not significant. (E) Hypothetical model summarizing the two SARS-CoV-2 infection mechanisms characterized here. See also Figure S5.

We previously showed that SARS-CoV-2 can use TMEM106B for the infection of several airway-derived cell types.^9^ As SARS-CoV-2 has been found to replicate in many organs,^50^^,^^51^^,^^52^ including organs that express ACE2 at low levels, such as the brain,^53^ we tested whether SARS-CoV-2 can use TMEM106B for the infection of several non-airway cell types derived from the intestines and brain. Analysis of ACE2 protein levels revealed very low or undetectable ACE2 expression in intestinal HIEC-6 cells, glioma-derived U-87 cells, patient-derived glioblastoma cells, and induced pluripotent stem cell (iPSC)-derived astrocytes (Figure S5E). *TMEM106B* knockout (Figure 5A) or treatment with TMEM106B-specific antibody Ab09 (Figures 5B–5D) blocked the SARS-CoV-2 infection of these cells. These results show that TMEM106B can support the SARS-CoV-2 infection of various cell types that have low ACE2 expression.

## Discussion

SARS-CoV-2 can use two different routes to enter host cells.^54^ The virus either fuses with the plasma membrane upon activation by the cell-surface protease TMPRSS2 or enters cells via endocytosis to fuse with the endo/lysosomal membrane upon activation by cathepsin proteases. Here, we show that TMEM106B and ACE2 can support separate infection mechanisms (Figures 4A and 4B). In NCI-H1975 cells, both infection mechanisms depend on viral endocytosis and cathepsin activity (Figure 4F). However, the observation that TMEM106B promotes the spike-mediated fusion of TMPRSS2-overexpressing cells (Figure 4I) suggests that TMEM106B might also be able to facilitate plasma membrane entry in other cell types that support this route. We found that a small fraction of TMEM106B resides on the cell surface (Figure S5B). Moreover, blocking that surface pool with anti-TMEM106B antibodies concurrently with virus addition was sufficient to prevent infection (Figure 2H). These data suggest that SARS-CoV-2 either engages TMEM106B on the cell surface or that the virus and (antibody-bound) TMEM106B are co-internalized, after which binding occurs inside endocytic vesicles. Although we cannot exclude that TMEM106B contributes to SARS-CoV-2 cell attachment and endocytosis, TMEM106B was dispensable for virus binding and endocytosis into cells (Figures 4G, 4H, and S5D), suggesting that the most critical function of TMEM106B is to promote a post-endocytic entry step. Indeed, we found that TMEM106B directly promotes cell-cell fusion (Figure 4I). TMEM106B-mediated infection is also enhanced by heparan sulfate (Figures 4C and 4D). Altogether, our data suggest a mechanism in which TMEM106B, together with heparan sulfate and other possible receptors, enables virus attachment and endocytic uptake, after which TMEM106B combined with cathepsin activity is required to facilitate membrane fusion (Figure 5E). Mechanistically, TMEM106B binding might stabilize conformational changes in spike, similar to those occurring upon ACE2 binding. ACE2 binding stabilizes conformational changes in spike that make the S2′ cleavage site more accessible and mobilize the fusion peptide, thereby priming spike for fusion.^55^^,^^56^ We speculate that in the absence of ACE2, TMEM106B may take over this function. Similar to ACE2 binding, the interaction of TMEM106B with the open state of spike might stimulate a progressive opening of S1 components^55^ by pushing the equilibrium of the different spike conformations toward the more open ones, which have increasingly accessible S2′ sites and fusion peptides.

Cryo-EM and HDX-MS analyses identified the RBD region 443–495 as the TMEM106B-binding site. The striking overlap in the footprints of ACE2 and TMEM106B on the RBD suggests that these receptors cannot simultaneously bind spike, as was confirmed by a competition assay (Figure S3D). Using targeted mutagenesis, we identified Met210 and Phe213 as TMEM106B residues crucial for spike binding (Figures 3E and 3F). We also showed that spike substitution E484D facilitates entry via the TMEM106B-dependent route (Figure 1F). Several studies have reported that E484D enables the SARS-CoV-2 infection of ACE2-negative cell lines,^30^^,^^31^ but the underlying mechanism remained unknown. Our structural data show that Asp484 is buried within the spike-TMEM106B interface (Figure 3C). Concordantly, spike containing Asp484 binds TMEM106B with higher affinity than the protein containing Glu484 (Figures 3D and S3C). Thus, Asp484 increases TMEM106B binding and enhances infection, demonstrating the ease with which the virus can adapt to an alternative receptor. However, viruses lacking this residue can also employ TMEM106B for infection (Figures 1B, 1C, and 3F). The observed affinity of 10 to 20 μM for the spike-TMEM106B interaction (Figure S3C) is much lower than what has been reported for the spike-ACE2 interaction (∼10–100 nM).^42^^,^^57^ However, it is similar to the affinity of SARS-CoV-2 spike binding to neuropilin-1 (10–20 μM)^46^ and higher than the affinity of, for example, Ebola virus binding to its primary receptor, the endosomal protein Niemann-Pick C1 (100–200 μM).^58^

Although we show that TMEM106B is essential for SARS-CoV-2 infection only in cells lacking high-level ACE2 expression, we speculate that TMEM106B might also play a role in ACE2-mediated infection. Indeed, a role of TMEM106B as a cofactor for ACE2-mediated SARS-CoV-2 entry was suggested by the simultaneous identification of *ACE2* and *TMEM106B* in a functional genomic screen for proviral genes.^13^ Moreover, *TMEM106B* knockout was shown to reduce SARS-CoV-2 infection in A549 cells that had been transduced with *ACE2* cDNA,^9^^,^^13^ although this might be due to modest ACE2 expression. In addition to its direct role as viral receptor, TMEM106B might influence SARS-CoV-2 infection via indirect mechanisms, which could help create a favorable environment for fusion. For instance, TMEM106B might influence luminal conditions in the endo/lysosomal compartments by regulating lysosome localization and maturation or, as was recently proposed,^29^ via a lipid transfer activity.

We show that TMEM106B-specific monoclonal antibodies can efficiently block SARS-CoV-2 infection when applied externally to cells. Such potent and specific antibodies may have scientific as well as therapeutic applications in the future. For instance, these reagents may be used to investigate the role of TMEM106B in SARS-CoV-2 infection *in vivo* and also to decipher the physiological function of TMEM106B in lysosome biology, which remains enigmatic. Although several monoclonal antibodies that directly target spike have been approved for COVID-19 therapy,^59^ they may have limited activity against new SARS-CoV-2 variants.^60^^,^^61^ This problem could potentially be circumvented by using monoclonal antibodies to block viral access to host factors.

Given the relevance of TMEM106B in multiple neurodegenerative disorders^62^ and cancer metastasis,^23^ it may be useful to develop modulators of TMEM106B activity for therapeutic applications. However, an *in vitro* assay to screen for such modulators of TMEM106B activity is not available at present. Here, we show that the SARS-CoV-2 infection of human cell lines can serve as a readout for TMEM106B inhibition, facilitating the identification of TMEM106B modulators. Moreover, the crystal structure of the TMEM106B LD (Figure 3A) can serve as a starting point for structure-based drug design efforts to develop small molecule compounds targeting TMEM106B. The sheer ubiquity of Fn3 domains and closely related immunoglobulin-like domains^35^ greatly complicates the prediction of TMEM106B function solely based on the structure of its LD. Comparing the TMEM106B^LD^ crystal structure with other known protein structures using the Dali server^63^ confirmed that it resembles the late embryogenesis abundant (LEA-2) protein, as recently predicted.^29^ However, the proposed lipid transfer function of TMEM106B^29^ requires experimental verification, and our crystal structure did not reveal a lipid-binding groove.

ACE2 is considered to be the main SARS-CoV-2 entry receptor for the infection of the respiratory epithelium, which is supported by the observation that SARS-CoV-2 RNA is most frequently detected in cell types that are also the dominant ACE2-expressing cell types.^64^^,^^65^ Nevertheless, whether SARS-CoV-2 infection is strictly limited to ACE2-expressing cells has not been established to date. Besides the respiratory tract, SARS-CoV-2 was found to reside in the gastrointestinal tract, heart, kidneys, blood, and brain,^50^^,^^51^^,^^52^ consistent with the multi-organ pathology observed in COVID-19. Although ACE2 expression is high in some of these organs (gastrointestinal tract, kidneys, and heart), it is very low in other, such as the lungs, liver, and brain.^53^ Considering the above points and the fact that a plethora of other SARS-CoV-2 receptor candidates was identified, it should not be assumed that ACE2 is the sole receptor mediating the infection of all SARS-CoV-2-permissive tissues and cell types. Several studies reported the infection of ACE2-negative cells,^30^^,^^31^^,^^32^ and here we show that SARS-CoV-2 can enter ACE2-deficient host cells via a TMEM106B-dependent entry mechanism. Thus, TMEM106B may facilitate virus entry into specific ACE2-negative cell types or tissues, possibly contributing to the multi-organ pathology seen in COVID-19. We showed that, for instance, intestine- and brain-derived cells with low or undetectable ACE2 expression support TMEM106B-mediated SARS-CoV-2 infection (Figures 5A–5D). We previously observed a correlation between SARS-CoV-2 infection and elevated *TMEM106B* expression in epithelial airway cells from COVID-19 patients.^9^ Further establishing TMEM106B as a relevant host factor in COVID-19 will require blocking TMEM106B function in animal models, along with an extensive analysis of the viral tropism and pathology caused by SARS-CoV-2 infection. As we found that mouse, hamster, and monkey TMEM106B also support SARS-CoV-2 infection (Figure S3H), these species could be suitable models for such animal studies. Finally, single-cell transcriptome analyses of different tissues from COVID-19 patients to correlate viral RNA levels with host gene expression could shed more light on the *in vivo* relevance of TMEM106B and other candidate receptors for SARS-CoV-2 infection.

### Limitations of the study

We show that spike substitution E484D enhances the binding and usage of TMEM106B but is not a prerequisite for TMEM106B binding. Our data also show that multiple SARS-CoV-2 isolates can use TMEM106B for infection, but mechanistic studies were mainly performed using a SARS-CoV-2 isolate that contains spike substitution E484D. It, therefore, remains to be fully established whether TMEM106B-dependent infection by SARS-CoV-2 isolates lacking the E484D substitution involves the same mechanism. Furthermore, since most infection experiments in this study were performed in immortalized cell lines, these analyses could be extended to fully differentiated organoid models and animal models in future studies.

## STAR★Methods

### Key resources table

**Table:** 

REAGENT or RESOURCE	SOURCE	IDENTIFIER
**Antibodies**		

Mouse anti double-stranded RNA (J2)	Scicons	Cat# 10010200; RRID: AB_2651015
Rabbit anti-SARS-CoV-2 Nucleocapsid	Rockland	Cat# 200-401-A50; RRID: AB_828403
Mouse anti-RSV F Synagis (Palivizumab)	Astra Zeneca	PC (product n°): 05000456067102; SN (series n°): 05503236717903
Mouse (humanized) anti-TMEM106B	Alector; this paper	Ab01 to Ab77
Mouse Anti-Human LAMP-1 (H4A3)	Santa Cruz Biotechnology	Cat# sc-20011; RRID: AB_626853
GAPDH (0411)	Santa Cruz Biotechnology	Cat# sc-47724; RRID: AB_627678
Human/Mouse/Rat/Hamster ACE-2 Antibody	R&D Systems	Cat# AF933; RRID: AB_355722
Goat anti-Rabbit IgG (H+L) Cross-Adsorbed Secondary Antibody; Alexa Fluor™ 568	Thermo Fisher Scientific	Cat# A-11011; RRID: AB_143157
Goat anti-Rabbit IgG (H+L) Cross-Adsorbed Secondary Antibody; Alexa Fluor™ 488	Thermo Fisher Scientific	Cat# A-11008; RRID: AB_143165
Goat anti-Human IgG (H+L) Cross-Adsorbed Secondary Antibody; Alexa Fluor™ 488	Thermo Fisher Scientific	Cat# A-11013; RRID: AB_2534080
Goat anti-Human IgG (H+L) Cross-Adsorbed Secondary Antibody, Alexa Fluor™ 568	Thermo Fisher Scientific	Cat# A-21090; RRID: AB_2535746
Goat anti-Mouse IgG (H+L) Highly Cross-Adsorbed Secondary Antibody; Alexa Fluor™ 488	Thermo Fisher Scientific	Cat# A-11029; RRID: AB_2534088
Alexa Fluor® 647 AffiniPure Goat Anti-Human IgG; F(ab')₂ fragment specific	Jackson Immuno Research	Cat# 109-605-006; RRID: AB_2337881
Anti-Mouse Secondary HRP Antibody	Protein Simple	Cat# 042-205; RRID: AB_2860576
Anti-Goat Secondary HRP Antibody	Protein Simple	Cat# 043-522-2; RRID:AB_2940933
Rabbit anti-Avi Tag antibody	R&D Systems	Cat# MAB10546; RRID: AB_2935823

**Bacterial and virus strains**		

One Shot™ Stbl3™ Chemically Competent *E. coli*	Thermo Fisher Scientific	Cat# C737303
SARS-CoV-2 isolate SARS-CoV-2/Belgium/GHB-03021/2020	Boudewijns et al.^66^	GenBank: MW368439
SARS-CoV-2 isolate SARS-CoV-2/Germany/BavPat1/2020	Christian Drosten	GenBank: MW368440
SARS-CoV-2 VOCα isolate hCoV-19/Belgium/rega-12211513/2020	Abdelnabi et al.^67^	GISAID: EPI_ISL_791333
SARS-CoV-2 VOCβ isolate hCoV-19/Belgium/rega-1920/ 2021	Abdelnabi et al.^67^	GISAID: EPI_ISL_896474
SARS-CoV-2 VOC omicron isolate hCoV-19/Belgium/rega-20174/2021	Johan Neyts; Abdelnabi et al.^68^	GISAID: EPI_ISL_6794907
HCoV-229E	ATCC	Cat# VR-740
RSV strain Long	ATCC	Cat# VR-26

**Biological samples**		

Hamster anti-SARS-CoV-2 serum	Kai Dallmeier and Johan Neyts	N/A

**Chemicals, peptides, and recombinant proteins**		

Camostat mesylate	Tokyo Chemical Industry	Cat# C2977
E64d	Tokyo Chemical Industry	Cat# E1337
Remdesivir	ACROS Organics	Cat# 469411000
Heparin	Sigma-Aldrich	Cat# H4784
Cycloheximide	Sigma-Aldrich	Cat# 01810
Heparan sulfate	Galen Laboratory Supplies	Cat# GAG-HS01
CellBrite Fix 640	Biotium	Cat# 30089
DAPI	Thermo Fisher Scientific	Cat# 3571
Paraformaldehyde	Sigma-Aldrich	Cat# 252549
Triton-X100	Sigma-Aldrich	Cat# 93443
X-TremeGENE9 DNA Transfection Reagent	Merck Life sciences	Cat# 6365809001
Gentamycin	Sigma-Aldrich	Cat# 1397
Puromycin	Sigma-Aldrich	Cat# P8833
Hygromycin	Thermo Fisher Scientific	Cat# 10687010
Blasticidin	Thermo Fisher Scientific	Cat# A11139-03
Antibiotic Antimycotic 100x	Thermo Fisher Scientific	Cat# 15240062
Polybrene	Santa Cruz	Cat# sc-134220
Crystal violet	Sigma-Aldrich	Cat# C3886
Live/Dead Near-IR stain	Thermo Fisher Scientific	Cat# L34975
Kifunensine	Sigma-Aldrich	Cat# K1140; CAS# 109944-15-2
Strep-Tactin®XT 4Flow® resin	IBA Lifesiences	Cat# 2-5010-002
BXT buffer	IBA Lifesiences	Cat# 2-1042-025
HisTrap Excel	Sigma-Aldrich	Cat# GE29-0485-86
Endoglycosidase H (Endo Hf)	New England Biolabs	Cat# P0703S
Tris(2-carboxyethyl)phosphine hydrochloride (TCEP)	Fisher Scientific	Cat# 15780329; CAS 5961-85-3
Formic Acid	Fisher Scientific	Cat# A117-50; CAS 64-18-6
Acetonitrile	Fisher Scientific	Cat# 15684740; CAS 75-05-8
Leucine Enkephaline	Waters	Cat# 186006013
Gu-HCl	Sigma-Aldrich	Cat# G4505; CAS 50-01-1
Deuterium Oxide	Sigma-Aldrich	Cat# 151882; CAS 7789-20-0
HiLoad® 16/600 Superdex® 200 pg	Sigma-Aldrich	Cat# GE28-9893-35
n-octyl glucoside	Sigma-Aldrich	Cat#10634425001
Poly-L-ornithine	Sigma-Aldrich	Cat# P4957
Geltrex	Thermo Fisher Scientific	Cat# A1569601
Laminin	Sigma-Aldrich	Cat# L2020
RIPA lysis buffer	Sigma-Aldrich	Cat# R0278
Stabilized trimeric Belgium/GHB-03021 SARS-CoV-2 spike ectodomain	This paper	N/A
Monomeric Belgium/GHB-03021 S1	This paper	N/A
TMEM106B^LD^	This paper	N/A
ACE2 ectodomain	Wrobel et al.^43^	N/A
Wuhan-Hu-1 SARS-CoV-2 S1(1-530)	Rosa et al.^66^	N/A
AviHis-TMEM106B^LD^ (AA118-274)	This paper	N/A

**Critical commercial assays**		

QIAamp DNA mini kit	Qiagen	Cat# 51304
CloneAmp HiFi PCR premix	Takara	Cat# 639298
Nucleospin Gel and PCR Clean-up	Machery-Nagel	Cat# 740609.50
CellTiter 96 AQueous One Solution Cell Proliferation Assay	Promega	Cat# G1111
CellsDirect™ One-Step qRT-PCR Kit	Thermo Fisher Scientific	Cat# 11753100
SARS-CoV-2 N1+N2 Assay Kit	Qiagen	Cat# 222015
TaqMan™ Gene Expression Assay (FAM) actin beta	Thermo Fisher Scientific	Cat# 4331182 Assay ID: Hs01060665_g1
NEBuilder HiFi DNA Assembly kit	New England Biolabs	Cat# E5520S
In-Fusion HD Kit	Takara	Cat# ST0345
Clonacell-HY Hybridoma Kit	Stem Cell Technologies	Cat# 03800
Expi293™ Expression System Kit	Thermo Fisher Scientific	Cat# A14635

**Deposited data**		

Crystal structure of TMEM106B^LD^	Protein Data Bank	PDB: 8B7D
Cryo-EM map of the spike-TMEM106B complex obtained by global consensus refinement	EM Data Bank	EMDB: EMD-17169
Cryo-EM map of the RBD-TMEM106B complex obtained by local refinement	EM Data Bank	EMDB: EMD-17170

**Experimental models: Cell lines**		

Human: HEK293T	Jason Moffat lab^69^	N/A
African green monkey: Vero E6	ATCC	Cat# CRL-1586; RRID: CVCL_0574
Human: Huh-7	CSL	Cat# 300156; RRID: CVCL_0336
Human: HCT-116	ATCC	Cat# CCL-247origin; RRID: CVCL_0291
Human: NCI-H1975	ATCC	Cat# CRL-5908; RRID: CVCL_1511
Human: HEp-2	ATCC	Cat# CCL-23; RRID: CVCL_1906
Human: CME035	Frederik De Smet^70^	NA
Human: CME036	Frederik De Smet^70^	NA
Human: CME038	Frederik De Smet^70^	NA
Human: iPSC-derived Astrocytes	Tempo Bioscience	Tempo-iAstro
Human: HIEC-6	ATCC	Cat# CRL-3266; RRID: CVCL_6C21
Human: U-87 MG	ATCC	Cat# HTB-14; RRID: CVCL_0022
Human: Monoclonal NCI-H1975 *TMEM106B*^KO^	This paper	N/A
Human: Monoclonal NCI-H1975 *ACE2*^KO^	This paper	N/A
Human: Monoclonal NCI-H1975 *ACE2*^KO^*EXT1*^KO^	This paper	N/A
Human: Monoclonal NCI-H1975 *TMEM106B*^KO^*+ ACE2* cDNA	This paper	N/A
Human: Monoclonal NCI-H1975 *TMEM106B*^KO^*+ ACE2* cDNA + *EXT1*^KO^	This paper	N/A
Hamster: BHK-21J	Peter Bredenbeek, LUMC, The Netherlands	N/A
Human: I1-Hybridoma	ATCC	Cat# CRL-2700; RRID: CVCL_G654
Human: HEK293	ATCC	Cat# CRL-1573; RRID: CVCL_0045
Human: A549	ATCC	Cat# CCL-185; RRID: CVCL_0023
Human: A549 expressing *TMEM106B* cDNA	This paper	N/A
Human: Expi293F	Gibco	CAT# A14527

**Experimental models: Organisms/strains**		

Mouse: NZBWF1/J (female)	Jackson Laboratory	RRID: IMSR_JAX:100008
Mouse: SJL/J (female)	Jackson Laboratory	RRID: IMSR_JAX:000686
Mouse: C57BL/6N TMEM106B knockout (female)	Taconic, Rensselaer, NY	N/A

**Oligonucleotides**		

qPCR primer 229E-FP: TCCGACGTGCTCGAACTTT	Vijgen et al.^71^	N/A
qPCR primer 229E-RP: CCAACACGGTTGTGACAGTGA	Vijgen et al.^71^	N/A
qPCR probe 229E-TP: FAM-TCCTGAGGT CAATGCA-NFQ-MGB	Vijgen et al.^71^	N/A
Oligonucleotides, synthetic genes and gBlocks: see Table S2	This paper	N/A

**Recombinant DNA**		

pMD2.G	Didier Trono	Addgene plasmid # 12259; RRID: Addgene_12259
psPAX2	Didier Trono	Addgene plasmid # 12260; RRID: Addgene_12260
pLentiCRISPRv2	Sanjana et al.^72^	Addgene plasmid # 52961; RRID: Addgene_52961
pLentiCRISPRv2-Hygro	This paper	N/A
pLCKO	Hart et al.^69^	Addgene plasmid # 73311; RRID: Addgene_73311
pLCKO-CMV-Luc-P2A-Blasti	This paper	N/A
pcDNA3.1-hACE2	Li et al.^73^	Addgene plasmid #1786; RRID: Addgene_1786
pLCKO-CMV-ACE2-P2A-Blasti	This paper	N/A
pLCKO-CMV-TMEM106B-P2A-Blasti	This paper	N/A
pLCKO-CMV-TMPRSS2-IRES-Hygro	This paper	N/A
pGACGG-nCOV19del18-FLAG	Berend Jan Bosch	N/A
pLCKO-CMV-SARS-CoV-2-S-Bel-P5_5-7-IRES-mNeonGreen-NES/PKI-P2A-Blasti	This paper	N/A
pCAGGS	Niwa et al.^74^	BCCM Cat# LMBP 2453
Plasmid: pCAGGS (KeraFAST EH1017) human TMEM106B (Uniprot Q9NUM4)	Rosenthal et al.^75^	N/A
Plasmid: pCAGGS (KeraFAST EH1017) cyno TMEM106B (Uniprot A0A2K5W4F7)	Rosenthal et al.^75^	N/A
Plasmid: pCAGGS (KeraFAST EH1017) mouse TMEM106B (Uniprot Q80X71)	Rosenthal et al.^75^	N/A
Plasmid: pCDNA3.1 human IgG1 and IgK	Rosenthal et al.^75^	N/A
Expression construct for Wuhan-Hu-1 S1 (residues 1-530)	Rosa et al.^76^	N/A
Expression construct for Belgium/GHB-03021 S1	This work	N/A
Expression construct for ACE2 ectodomain (residues 1-615)	Wrobel et al.^77^	N/A
Expression construct for TMEM106B ectodomain.	This work	N/A
Expression construct for Belgium/GHB-03021 spike ectodomain stabilized with hexa-pro mutations	This work	N/A

**Software and algorithms**		
Geneious Software (v9.1.8)	Geneious	http://www.geneious.com/; RRID: SCR_010519
HCS Studio Cell Analysis software (v 6.6.0)	Thermo Fisher Scientific	RRID: SCR_016787
Cell Profiler (v4.2.4)	Stirling et al.^78^	https://cellprofiler.org/
DIALS	Winter et al.^79^	https://dials.github.io/
Xia2	Winter et al.^80^	https://xia2.github.io/index.html
Phenix	Liebschner et al.^81^	http://www.phenix-online.org/
Phaser	McCoy et al.^82^	https://www.phaser.cimr.cam.ac.uk/index.php/Phaser_Crystallographic_Software
MolProbity	Chen et al.^83^	http://molprobity.manchester.ac.uk/
AlphaFold	Jumper et al.^84^	https://alphafold.ebi.ac.uk/
Coot	Emsley et al.^85^	https://www2.mrc-lmb.cam.ac.uk/personal/pemsley/coot/
MotionCor-2	Zheng et al.^86^	https://emcore.ucsf.edu/ucsf-software
Gctf (v1.06)	Zhang et al.^87^	https://www2.mrc-lmb.cam.ac.uk/download/gctf_v1-06-and-examples/
SPHIRE-crYOLO	Wagner et al.^88^	https://cryolo.readthedocs.io/en/stable/
cryoSPARC (v3)	Punjani et al.^89^	https://cryosparc.com/
cryoSPARC (v4)	Punjani et al.^89^	https://cryosparc.com/
Topaz	Bepler et al.^90^	https://guide.cryosparc.com/processing-data/all-job-types-in-cryosparc/deep-picking/topaz
MicrographCleaner	Sanchez-Garcia et al.^91^	https://github.com/rsanchezgarc/micrograph_cleaner_em
Relion (v4.0)	Scheres et al.^92^^,^^93^; Kimanius et al.^25^	https://relion.readthedocs.io/en/release-4.0/
UCSF Chimera	Pettersen et al.^94^	https://www.cgl.ucsf.edu/chimera/
ProteinLynx Global SERVER (PLGS) (v3.0)	Waters	https://www.waters.com/waters/en_US/ProteinLynx-Global-SERVER-(PLGS)/nav.htm?cid=513821&locale=en_US
DynamX (v3.0)	Waters	https://www.waters.com/waters/library.htm?locale=en_US&lid=134832928
Compass for Simple Western (v6.1.0)	Protein Simple	https://www.bio-techne.com/resources/instrument-software-download-center/compass-software-simple-western; RRID: SCR_022930

**Other**		

400-mesh copper R1.2/1.3 holey carbon grids (Quantifoil)	EMS	Cat# Q4100CR1.3
ACQUITY UPLC BEH C18 VanGuard Pre-column	Waters	Cat# 86003975
ACQUITY UPLC BEH C18 Analytical Column	Waters	Cat# 186002352
Dual Protease column (Pepsin: Type XIII 1:1)	Novabioassays	Cat# NBA2014002

### Resource availability

#### Lead contact

Further information and requests for resources and reagents should be directed to and will be fulfilled by the lead contact, Dirk Daelemans (dirk.daelemans@kuleuven.be).

#### Materials availability

• Plasmids generated in this study are available from the lead contact upon request.
• Cell lines generated in this study are available from the lead contact upon request.
• Certain reagents may not be available due to restrictions based on limited quantities and/or proprietary nature of the materials.

### Experimental model and subject details

#### Cell lines

All cell lines were maintained at 37 °C under 5% CO_2_ and routinely tested for contamination by mycoplasma. HEK293T (obtained from Jason Moffat, University of Toronto), Vero E6 (ATCC- CRL-1586), HEp-2 (ATCC CCL-23), U-87 MG (ATCC HTB-14) and Huh-7 (CLS - 300156; human hepatoblastoma) were grown in Dulbecco’s Modified Eagle Medium (DMEM). HCT-116 cells (ATCC CCL-247origin) were maintained in McCoy’s 5A medium. NCI-H1975 cells (ATCC-CRL-5908) were maintained in RPMI medium. BHK21J were grown in MEM, 10 mM Hepes (ThermoFisher), 1x MEM Non-Essential Amino Acids Solution (ThermoFisher), 2 mM L-glutamine (ThermoFisher), 0.075% sodiumbicarbonate (ThermoFisher) and 100 U/ml PenStrep (ThermoFisher). I1-hybridoma were grown in DMEM glutamax, 1x HT supplement (ThermoFisher), 2 mM sodium pyruvate (ThermoFisher), 1x MEM Non-Essential Amino Acids Solution (ThermoFisher), 50 μM 2-mercapto-ethanol (ThermoFisher) and 100 U/ml PenStrep (ThermoFisher). The above media were supplemented with 10% heat-inactivated fetal bovine serum (HyClone). Patient-derived glioblastoma (PD-GBM) cell lines CME035, CME036 and CME038 are approved by the Ethical Comission Research UZ/KU Leuven (S67312). They were grown in Neurocult NS-A medium, supplemented with 0.1% heparin, 20 ng/mL recombinant EGF, 20 ng/mL human recombinant BFGF and 1:100 Antibiotic Antimycotic. For seeding of PD-GBM in multiwell plates, plates were coated with laminin. Human iPSC-derived Astrocytes (Tempo-iAstro™) were grown in DMEM/F12 supplemented with 5% FBS (HyClone). Culture vessels for astrocyte subcultivation were coated with Geltrex and multiwell plates were coated overnight with poly-L-lysine and 1 hour prior to cell seeding coated with an additional layer of laminin. HEK293 and A549 were cultured in DMEM, supplemented with 1% penicillin/streptomycin (Gibco) and 10% heat-inactivated fetal bovine serum (Cytiva). A549 TMEM106B overexpressing cells were maintained in 1μg/ml puromycin (Gibco) antibiotic selection. Expi293 cells were cultured in Expi293 Expression Medium without supplement (Gibco). HIEC-6 (ATCC CRL-3266) were grown in OptiMEM 1 Reduced Serum Medium supplemented with 20 mM HEPES, 10 mM GlutaMAX, 10 ng/mL Epidermal Growth Factor (EGF) and 4% heat-inactivated FBS.

#### Mice

Six- to eight-week-old female NZB/W mice and SJL mice were obtained from Jackson Laboratory, Bar Harbor, ME. *TMEM106B* knockout mice were obtained from Taconic, Rensselaer, NY. All animal studies were conducted under protocols approved by the Alector Institutional Animal Care and Use Committee. Mice were housed in a pathogen-free, climate-controlled facility and given food and water ad libitum.

#### Virus strains

All virus-related work was conducted in the high-containment biosafety level 3 facilities of the Rega Institute from the KU Leuven (Leuven, Belgium), according to institutional guidelines. Virus stocks were produced by seeding cells in their recommended growth medium to reach a confluency of ∼80% the next day. After replacing the medium by DMEM + 4% fetal bovine serum, cells were infected with virus at a MOI of ∼0.01. When most cells were dying, supernatant was removed from the cells, centrifuged to remove cell debris and stored at -80 °C. SARS-CoV-2 isolate SARS-CoV-2/Belgium/GHB-03021/2020 (GenBank accession number MW368439) was recovered from an asymptomatic COVID-19 patient returning from Wuhan in February 2020, as described previously,^66^ and was propagated by serial passaging in Huh7 cells. Stocks containing spike substitution E484D were used unless otherwise indicated. SARS-CoV-2 isolate SARS-CoV-2/Germany/BavPat1/202065 (GenBank accession number MW368440) was obtained from Prof. Christian Drosten, Charité Universitätsmedizin Berlin and passaged twice on Vero E6 cells. The SARS-CoV-2 isolate belonging to VOCα (hCoV-19/Belgium/rega-12211513/2020; EPI_- ISL_791333, 2020-12-21) was isolated from a nasopharyngeal swab taken from a healthy subject that returned to Belgium in December 2020.^67^ The VOCβ virus (hCoV-19/Belgium/rega-1920/ 2021; EPI_ISL_896474, 2021-01-11) was isolated from a nasopharyngeal swab from a patient with respiratory symptoms that returned to Belgium in January 2021.^67^ The VOC omicron virus was isolated from a nasopharyngeal swab taken from a traveler returning to Belgium at the end of November 2021 (hCoV-19/Belgium/rega-20174/2021, EPI_ISL_6794907).^68^ These VOC isolates were all propagated in Vero E6 cells. HCoV-229E (ATCC VR-740) was propagated in Huh7 cells and RSV (strain Long, ATCC VR-26) was propagated in HEp-2 cells.

### Method details

#### Lentiviral vector production

To produce lentivirus particles, HEK293T cells were plated in 40 mL DMEM with 10% fetal bovine serum in T150 (TPP) flasks at 45% confluency and incubated overnight. After 1 day, cells were transfected using X-TremeGENE 9 (Roche) with a transfer plasmid, together with the packaging plasmids psPAX2 and envelope plasmid pMD2.G and incubated overnight to form lentiviral particles coated with the VSV-G protein. ∼16 h post-transfection, medium was replaced by serum-free growth medium (DMEM + 1.1 g/100 mL BSA and 20 μg/mL gentamicin). Supernatant containing lentivirus was harvested ∼48 h after transfection and stored at -80 °C.

#### Generation of knockout cell lines

For knockout of a specific gene, sgRNAs targeting the gene were cloned into the pLentiCRISPRv2 plasmid (Addgene 52961) following the standard cloning protocol. *TMEM106B*^KO^ and *ACE2*^KO^ cells were generated by transducing cells with a pool of four sgRNAs targeting *TMEM106B* or *ACE2* (taken from the Brunello genome-wide knockout library) and selecting with puromycin (1 μg/ml) for 3 days. Monoclonal NCI-H1975 *EXT1* knockout cells were made by cloning *EXT1* sgRNA #3 from the Brunello library into pLentiCRISPR containing a hygromycin resistance gene instead of the puromycin resistance gene, followed by transduction and hygromycin selection. Monoclonal cells were made by seeding a dilution series of cells and selecting wells containing a single cell colony. To verify knockout, genomic DNA was isolated from cells with the QIAamp DNA mini kit using RNase A. First, sgRNAs present in the monoclonal cells were identified by Sanger sequencing. sgRNA expression cassettes were amplified using the CloneAmp HiFi PCR premix (Clontech) in 25μl PCR reactions containing 50 ng genomic DNA. The amplified DNA was then purified (Nucleospin® Gel and PCR Clean-up (Macherey-Nagel)), Sanger sequenced (Macrogen), and analysed with Geneious Software (v9.1.8). Then, genomic sequences surrounding the sgRNA cut sites were amplified by PCR as described above, followed by a second PCR with primers containing Illumina adapter sequences. Illumina sequencing was performed to verify that the coding sequence was disrupted. Polyclonal NCI-H1975 *TMEM106B* and *ACE2* double knockout cells were made by transducing monoclonal *ACE2*^KO^ cells with a pool of 4 sgRNAs targeting *TMEM106B*, followed by puromycin selection. For sgRNA sequences and PCR primers, see Table S2.

#### Cells overexpressing ACE2 or TMEM106B

The pLCKO plasmid was a gift from Jason Moffat (Addgene plasmid #73311). The gRNA scaffold and the puromycin resistance gene were removed and replaced by the coding sequence of the *Photinus pyralis luc2* gene (*luc*), human ACE2 (Addgene Plasmid #1786), or human, mouse, hamster, or African green monkey *TMEM106B* (Integrated DNA Technologies), followed by a P2A-coupled blasticidin resistance gene driven by a cytomegalovirus promotor. sgRNA target sequences of sgRNAs from the Brunello library, as well as PAM sequences flanking these sites, were mutagenized in the coding sequence of *TMEM106B* by introducing silent mutations. Single and double amino acid substitutions in *TMEM106B* were introduced by PCR. The resulting vector was used to make lentiviral particles, as described above. Cells were transduced with lentivirus stock in the presence of polybrene (8 μg/ml). The next day, medium was replaced by medium containing blasticidin (10 μg/ml) and cells were incubated for an additional 2-3 days. For A549 TMEM106B overexpressing cells, parental A549 cells (ATCC CCL-185) were transfected with a pD2539-PURO vector (DNA 2.0) containing the sequence for human TMEM106B under EF-1α promoter (DNA 2.0). TMEM106B-high cells were selected using puromycin and maintained in culture at 1μg/ml puromycin. For gene sequences, see Table S2.

#### Cell viability assays

Cells were seeded in 96-well plates in medium with 8% fetal bovine serum. The following day, virus in serum-free medium was added to cells, resulting in a serum concentration of 4%. Cells were incubated at 35 °C until sufficient CPE was visible. For MTS assays, medium was removed from the cells and replaced by MTS reagent (CellTiter 96 AQueous One Solution Cell Proliferation Assay from Promega, Madison, WI) diluted in PBS. The absorbance was measured with a Tecan Spark microplate reader. For crystal violet staining, cells were fixed in 4% formaldehyde for 30 min, stained with a 1% crystal violet solution in water, and rinsed with water.

#### Quantitative PCR

To assess virus replication, cells were infected with virus for 1 h at 35 °C in DMEM without fetal bovine serum. After infection, the inoculate was replaced by DMEM with 4% fetal bovine serum. At different timepoints, supernatant was removed, and cells were frozen at -80 °C. Cell lysis and quantitative PCR were performed using the CellsDirect™ One-Step qRT-PCR Kit (Invitrogen), with primers and probes specific for SARS-CoV-2 (SARS-CoV-2 N1+N2 Assay Kit, Qiagen Catalog 222015) or the internal control actin (Taqman gene expression assay, ThermoFisher 4331182). qPCR on HCoV-229E RNA was performed using primers and probes 229E-FP, 229E-RP and minor groove binder (MGB) probe 229E-TP as described previously.^71^ Amplification and detection were performed in an QuantStudio™ 5 Real-Time PCR System (Applied BiosystemsTM). Ct values were corrected using actin mRNA levels and converted to relative RNA levels. To determine SARS-CoV-2 binding to cells, NCI-H1975 cells were incubated with virus on ice for 1 hour and washed three times with PBS. Cell lysis and qPCR for SARS-CoV-2 was performed as described above.

#### Pseudovirus assays

Expression plasmids encoding SARS-CoV-2-S were obtained from Berend Jan Bosch, Utrecht University, The Netherlands. Mutations in the S sequence were introduced via PCR and inserted into pCAGGS using the NEBuilder HiFi DNA Assembly kit. First, two deletions (68-76 and 676-680) and substitution S813I were introduced to match the sequence of SARS-CoV-2/Belgium/GHB-03021/2020 passage 6, followed by changing residue E484 to either D, K, or A. VSV spike-pseudotyped viruses were generated as described previously.^95^ Briefly, BHK-21J cells were transfected with the SARS-CoV-2 spike protein expression plasmid, and one day later infected with GFP-encoding VSVΔG backbone virus.^96^ Two h later, the medium was replaced by medium containing anti-VSV-G antibody (I1-hybridoma, ATCC CRL-2700) to neutralize residual VSV-G input. After 26 h incubation at 32 °C, the supernatants were harvested and used for infection experiments. To test the infectivity of pseudoviruses, cells were seeded in 96-well plates in DMEM with 8% fetal bovine serum, inoculated with pseudotyped VSV on the next day, and incubated at 37 °C. One day after infection, cells were fixed with 4% formaldehyde. The percentage of GFP expressing cells was quantified on a Cell Insight CX5/7 High Content Screening platform (Thermo Fischer Scientific) with Thermo Scientific HCS Studio (v 6.6.0) software.

#### Immunofluorescence assays

Cells were seeded at a density of 20,000 cells per well in 8-well μ-slides (Ibidi) or a density of 8,000 cells per well in 96-well plates. Cells were allowed to adhere for several hours before receiving compound treatment. After overnight incubation, virus was added to the medium and cells were incubated at 35 °C. After incubation, cells were fixed (4% PFA in PBS), washed and permeabilized (0.2% Triton X-100 in PBS). Fixed cells were stained with mouse anti-dsRNA (J2, Scicons) at a 1:1000 dilution, rabbit anti-SARS-CoV-2 nucleocapsid (Rockland) at a 1:500 dilution, mouse anti-RSV F (Synagis (Palivizumab), AstraZeneca) at a 1:5000 dilution, mouse (humanized) anti-TMEM106B Ab09 (Alector) at 1:200 dilution, or mouse anti-LAMP-1 (Santa Cruz Biotechnology sc-20011) at 1:200 dilution. Secondary antibodies Alexa Fluor® 568 goat anti-rabbit (A11011, Invitrogen, ThermoFisher Scientific), Alexa Fluor® 488 goat anti-rabbit (A-11008, Thermo Fisher Scientific) Alexa Fluor® 488 goat anti-human (A11013, Thermo Fisher Scientific), and Alexa Fluor® 488 goat anti-mouse (A11029, Thermo Fisher Scientific) were diluted 1:500. The percentage of infected cells was quantified by high-content imaging analysis (CellInsight CX5, ThermoFisher Scientific) employing the HCS Studio Cell Analysis software (ThermoFisher Scientific). To monitor SARS-CoV-2 internalization, cells were fixed and sequentially stained with rabbit anti-SARS-CoV-2 nucleocapsid, goat-anti-rabbit-A488, permeabilized, stained again with rabbit anti-SARS-CoV-2 nucleocapsid, and finally goat-anti-rabbit-A568. Membrane staining was performed by incubating cells for 30 min at ambient temperature with CellBrite Fix 640 (Biotium), before fixing the cells. Cell nuclei were counterstained with DAPI and the samples were imaged by confocal microscopy on a Leica TCS SP5 confocal microscope (Leica Microsystems) using a HCX PL APO 63x (NA 1.2) water immersion objective. For quantification, 6 images from each condition were taken at zoom factor 1, representing 113 to 159 cells. Cell Profiler (v4.2.4) software was used to process images and quantify both internalized (red only) and total (red+green) signal. For analysis of the localization of anti-TMEM106B Ab09 antibody, we performed a sequential labeling assay. Cells were seeded at a density of 15,000 cells per well in an 18-well μ-slide (Ibidi). The next day live cells were blocked for 15’, incubated with a 1:200 dilution of Ab09 for 120’, washed and incubated for 15’ with secondary Alexa Fluor® 488 goat anti-human antibody (A11013, Thermo Fisher Scientific). Cells were then fixed and permeabilized as described above and next incubated for 15’ with 1:500 Alexa Fluor™ 568 Goat anti-Human secondary antibody (A21090, Thermo Fisher Scientific). Nuclei were counterstained with DAPI. All treatments were performed at room temperature. Samples were imaged on a Leica TCS SP5 confocal microscope (Leica Microsystems).

#### Neutralization assays

In neutralization assays with anti-TMEM106B antibodies, cells were seeded and pretreated with antibody on the same day, unless otherwise indicated. Following overnight incubation, virus was added to the medium and cells were incubated at 35 °C for the indicated time. In neutralization assays with heparin, the virus stock was pretreated for 30 min, followed by addition of virus to the cells and incubation at 35 °C for the indicated time.

#### Time of drug addition assay

NCI-H1975 cells were seeded in 96-well plates at 10,000 cells per well in RPMI with 4% fetal bovine serum. Compounds or antisera were added to cells at the indicated timepoints before and after virus infection. On the day after seeding, virus was added to the media, followed by 60 min incubation at 35 °C. After infection, supernatant was removed from the cells and replaced by new RPMI with 4% fetal bovine serum. Compounds or antisera were added again for conditions treated before or during infection. At 11 h post-infection, supernatant was removed, and cells were frozen at -80 °C for qPCR analysis as described above.

#### Generation of anti-TMEM106B antibodies

Six to eight week old female NZB/W mice, SJL mice (Jackson Laboratory, Bar Harbor, ME), and C57BL/6N *TMEM106B* knockout mice (Taconic, Rensselaer, NY) were co-immunized with plasmid DNA encoding full-length human, cyno, and mouse TMEM106B. Hybridoma fusions were performed using splenocytes from mice whose sera demonstrated strong binding to HEK293 cells transiently overexpressing either human, cyno, or mouse TMEM106B. IgG-producing hybridomas were isolated using the Clonacell-HY Hybridoma Kit (Stemcell Technologies, Vancouver, BC) and Clonepix2 colony picker (Molecular Devices, Sunnyvale, CA), and supernatants were screened by FACS on HEK293 cells transiently overexpressing *TMEM106B*. The variable heavy and light chain sequences of positive hybridoma clones were sequenced and cloned into mammalian expression vectors encoding a human IgG1 and IgK, respectively. The resulting chimeric antibodies were expressed in Expi293 cells using Expifectamine 293 (ThermoFisher) according to the manufacturer’s protocol. The recombinant chimeric human IgG1 antibodies were purified using Protein A.

#### Anti-TMEM106B antibody cell binding

A549 human lung carcinoma cells stably overexpressing *TMEM106B* (A549 *TMEM106B* cDNA) were incubated with 10 μg/ml human recombinant anti-TMEM106B test antibodies or human IgG1 isotype control for 30 min at 4 °C. After washes, the cells were incubated with an Alexa Fluor 647 F(ab’)2 fragment Goat anti-human IgG secondary antibody (Jackson ImmunoResearch) for 20 min at 4 °C, followed by Live/Dead Near-IR (Molecular Probes) stain for 15 min at room temperature. Fluorescence intensity was determined on a LSRFortessa flow cytometer (BS Biosciences). Dead cells and doublets were excluded from the analysis and the Alexa 647 geometric mean fluorescence intensity (GMFI) was determined on the remaining population. The GMFI ratio of anti-TMEM106B to hIgG1 isotype control was calculated for each clone.

#### Surface plasmon resonance

Binding studies were carried out using a Biacore T200 (Cytiva Life Sciences). To prepare the sensor surface, rabbit anti-Avi (R&D Systems) was immobilized on a CM5 sensor chip by amine coupling. Briefly, antibody was diluted to 10 μg/mL in 10 mM NaOAc buffer, pH 4.25 and injected for 7 min at 10 μL/min over a chip that had been activated with NHS/EDC mixture according to the manufacturer’s instructions. Remaining functional groups on the chip were blocked by injecting 1M Ethanolamine pH 8.5 (Cytiva). Recombinant AviHis-TMEM106B^LD^ was diluted to 300 nM in running buffer (HBS-EP+ (Teknova, Hollister, CA) with 0.1 mg/mL BSA) and captured by injecting over the immobilized anti-Avi antibody for 2 min at 10 μL/min. As control, buffer was injected during the capture step. TMEM106B-specific antibodies were diluted to 30 μg/mL in running buffer and injected over the captured AviHis-TMEM106B^LD^ for 2 min at 10 μL/min.

#### Recombinant proteins

For expression of stabilized trimeric Belgium/GHB-03021 SARS-CoV-2 spike ectodomain or monomeric Belgium/GHB-03021 S1, codon-optimized DNA fragments encoding the ectodomain region carrying HexaPro substitutions^36^ or spike residues 1-530 (both followed by TwinStrep tags) were cloned into pcDNA3. For production of TMEM106B^LD^, a codon-optimized DNA fragment encoding residues 118-274 of human TMEM106B, with a signal peptide derived from the immunoglobulin kappa gene product (METDTLLLWVLLLWVPGSTGDAAQ) and a C-terminal hexahistidine tag cleavable with TEV protease (GSGENLYFQSAGHHHHHH) was cloned into pcDNA3.

Recombinant proteins were produced in Expi293 (Thermo Fisher Scientific) cells transfected with endotoxin-free preparations of the corresponding DNA constructs using ExpiFectamine293 (Thermo Fisher Scientific). The cells were maintained in shake flasks in FreeStyle293 (Thermo Fisher Scientific) medium at 37°C in humidified 5% CO_2_ atmosphere. To produce TMEM106B^LD^ for crystallography, cell culture medium was supplemented with 1 mg/L kifunensine (Sigma-Aldrich) to inhibit complex glycosylation (39). Conditioned medium containing recombinant product was harvested 5 days post-transfection.

Strep-tagged proteins were captured on Strep-Tactin XT (IBA Lifesciences) resin. Following extensive washes in TBSE (150 mM NaCl, 1 mM EDTA, and 25 mM Tris-HCl, pH 8.0), the proteins were eluted in BXT buffer (IBA Lifesciences). Hexahistidine-tagged TMEM106B^LD^ was captured on HisTrap Excel (Sigma-Aldrich) resin and eluted with 200 mM imidazole in TBS (150 mM NaCl and 25 mM Tris-HCl, pH 8.0). For the use in crystallography, TMEM106B^LD^ was treated with Endo Hf (New England Biolabs) and TEV protease to trim glycans and to remove the hexahistidine tag. The protein was further purified by size exclusion chromatography through a Superdex 200 16/600 column (GE Healthcare) in TBS. Recombinant ACE2 ectodomain and Wuhan-Hu-1 SARS-CoV-2 S1(1-530) were described previously.^42^^,^^76^

The Avi-His-TMEM106B luminal domain (AviHis-TMEM106B-LD) recombinant protein contains residues 118-274 of transmembrane protein 106B [Homo sapiens], accession # NP_001127704.1, preceded by a secretion signal (MGWSCIILFLVATATGVHS) and an Avi-His tag (GLNDIFEAQKIEWHEHHHHHHHHHGG) at the N-terminus. This protein was produced in Expi293 (Gibco) cells and purified on a Ni-NTA resin (Thermo Scientific) using standard methods.

#### X-ray crystallography

TMEM106B^LD^, partially deglycosylated by digestion with Endo Hf, was crystallized in sitting drops comprising 0.2 μl protein solution (16 mg/ml in TBS) and 0.2 μl reservoir solution (2.0 M NaCl, 0.1 M BisTris-HCl, pH 5.5, corresponding to condition G12 of JCSG-plus crystallization screen; Molecular Dimensions) at 18 °C. Crystals, supplemented with 25% glycerol in reservoir solution, were flash-frozen in liquid nitrogen. X-ray diffraction data were collected on beamline I24 of the Diamond Light Source (Oxfordshire, UK). Diffraction images, collected with a 0.2° oscillation, 0.2 sec, 100% transmission and 30x30 μm beam, were indexed, integrated, scaled and merged using DIALS software^79^ via Xia2 pipeline^80^ (Table S1). The structure was solved by molecular replacement using Phaser^82^ within the Phenix software suite.^81^ The search model was prepared from the TMEM106B model produced by AlphaFold^84^ (https://alphafold.ebi.ac.uk/entry/Q8N353) using phenix.process_predicted_model software. Molecular replacement generated a single high-quality solution with a translation Z score and a log likelihood gain of 20.2 and 280.4, respectively. The structure was refined interactively in real-space in Coot,^85^ and in reciprocal and real space using phenix.refine.^97^ Four NAG residues were added to Asn residues 145, 151, 164, and 256, guided by the difference electron density map. There is additional, uninterpretable, difference density adjacent to Lys178-Leu181 and Gln194-Thr198 on the outside of the beta-barrel which can be assumed to be partially ordered N or C terminal residues. The final model, spanning TMEM106B residues 118-261, had good fit to the electron density map and reasonable geometry as assessed by MolProbity^83^ (Table S1).

#### Cryo-EM data collection and image processing

Four μl Belgium/GHB-03021 spike ectodomain trimer (1 mg/ml) supplemented with TMEM106B^LD^ (1 mg/ml) and 0.1% n-octyl glucoside in TBS was applied to glow-discharged 400-mesh copper R1.2/1.3 holey carbon grids (Quantifoil) for 1 min, under 100% humidity at 20°C, before blotting and plunge-freezing in liquid ethane using Vitrobot Mark IV (Thermo Fisher Scientific). Cryo-EM data were acquired on a 300-kV Titan Krios G3i cryo-electron microscope (Thermo Fisher Scientific) equipped with a Gatan GIF BioQuantum energy filter and a K3 Summit direct electron detector (Gatan). Micrographs were recorded in dose-fractionation mode, at a calibrated magnification corresponding to 0.85 Å per physical pixel (0.425 Å per super-resolution pixel) at the detector level. Two batches of data were collected, each from an independently vitrified grid. The batches comprised 18,806 and 18,273 micrograph movies recorded with a total exposure of 30 e/Å^2^, which was fractionated over 28 and 32 frames, respectively. A total electron exposure of 30 e/Å^2^ was fractionated across 28 movie frames. A 20-eV energy slit and a defocus range of -0.5 to -2.1 μm was used to acquire the data.

The movie stacks were aligned, binned to the physical pixel size (0.85 Å) and summed, with dose weighting as implemented in MotionCor-2.^86^ Contrast transfer function parameters were estimated using Gctf-v1.06.^87^ Following discarding of imaged exhibiting evidence of crystalline ice contamination, 33,876 frame sums were retained in further processing. An initial set of particles picked with SPHIRE-crYOLO, using general model^88^ was subjected to reference-free 2D classification in cryoSPARC-3.^89^ Particles belonging to well-defined 2D classes were used for training particle picking using Topaz.^90^ Particles picked along carbon edges or ice contamination were removed using MicrographCleaner.^91^ A set of remaining 1,799,554 particles, extracted with a pixel size 4.25 Å and a box size 84 pixels, was subjected to several rounds of reference-free 2D classification in cryoSPARC-3 into 300 classes. 270,810 particles belonging to well-defined 2D classes (Figure S2B) were re-extracted with pixel size 2.55 Å were subjected to 45 cycles of classification in Relion-4.0^92^^,^^93^ into five 3D classes; the initial model was generated by low-pass filtering a previously reported SARS-CoV-2 spike cryo-EM structure (EM Data Bank entry EMD-12586).^76^ A single high-resolution 3D class comprising 201,270 particles (Figure S2C) was subjected to non-uniform 3D refinement in cryoSPARC-3. The resulting volume revealed features consistent with a small protein bound to the erect RBD (circled in Figure S2D). To enrich particles containing the bound protein, we carried out subtraction of the bulk of spike density, leaving only the three RBDs plus the additional density (the latter was masked by a sphere). Subtracted particles were subjected to 45 cycles of 3D classification without realignment in Relion-4.0 into 4 classes, while imposing a soft spherical mask enclosing the bound protein. Three of the resulting classes, collectively comprising ∼38% input particles, displayed the additional density (Figure S2E). The best-defined class including 25,781 particles was used in the final 3D reconstruction using nonuniform refinement procedure implemented in cryoSPARC-4.1 resulting in a global resolution of 3.52 Å (Figure S2F), while the local resolution around the bound TMEM106B^LD^ subunit was ∼7 Å (Figure S2G). The local map quality was improved by local refinement procedure in cryoSPARC-4.1, using a soft mask enclosing the TMEM106B^LD^ along with its associated RBD (Figures 3C and S2H). Relion-4.0 UCSF Chimera^94^ was used for rigid body docking of SARS-CoV-2 spike and TMEM106B^LD^ structures into cryo-EM maps (Figure 3C). The resolution metrics reported here are according to the gold-standard Fourier shell correlation (FSC) 0.143 criterion^98^^,^^99^ (Figures S2F and S2H). Local resolution of the 3D reconstruction was estimated in cryoSPARC-4.1 (Figure S2G).

#### Hydrogen-deuterium exchange mass spectrometry

Prior to conducting HDX-MS, peptides were identified by digesting undeuterated Belgium/GHB-03021 S1 using the same protocol and identical liquid chromatographic (LC) gradient as detailed below and performing MS^E^ analysis with a Synapt G2-Si mass spectrometer (Waters), applying collision energy ramping from 20-30 kV. Sodium iodide was used for calibration and Leucine Enkephalin was applied for mass accuracy correction. MS^E^ runs were analyzed with ProteinLynx Global Server (PLGS) 3.0 (Waters) and peptides identified in 3 out of 4, with at least 0.2 fragments per amino acid (at least 2 fragments in total) and mass error below 10 ppm were selected in DynamX 3.0 (Waters). To note, post-quenching deglycosylation was not performed and no attempt to identify glycosylated peptides was made.

For HDX experiments, 19.5 pmol Belgium/GHB-03021 S1 were incubated alone and in the presence of 68 pmol TMEM106B^LD^ (molar ratio 1:3.5) in a total volume of 1 μL per time point. After equilibration, the labelling was performed with deuterated PBS (pH_read_ 7.35) for several time points and under three different temperatures (10 s on ice; 10 s, 1 min, 10 min and 100 min at room temperature (∼23 °C); 100 min and 360 min at 28 °C) to expand the dynamic and time window studied. Given the K_d_ in the micromolar range, to avoid the complex falling apart, proteins were labelled upon dilution into a low volume of labelling buffer, as recommended by Hamuro et al.^100^ The exchange reactions were initiated by 5-fold dilution into deuterated PBS (with 4 μL of deuterated buffer per time point) and quenched 1:3 (v/v) with an ice-cold buffer containing 100 mM phosphate buffer with 4 M urea and 0.5 M Tris(2-carboxyethyl)phosphine hydrochloride (TCEP) (66% v/v) and labelling buffer (33% v/v) (final pH_read_ 2.3), to increase the injection volume. Samples were held on ice for 30 s and snap-frozen in liquid nitrogen. Triplicates were performed for time points 10 s and 100 min at room temperature, and 360 min at 28 °C; duplicates were performed for all others time points. The labelled samples were kept at -80 °C until LC-MS analysis.

Protein samples were quickly thawed and injected into an Acquity UPLC M-Class System with HDX Technology (Waters). Proteins were on-line digested at 20 °C into a dual protease column (Pepsin-Type XIII 1:1; NovaBioAssays) and trapped/desalted with Solvent A (0.23% formic acid in water, pH 2.5) for 3 min at 200 μL/min and at 0 °C through an Acquity BEH C18 VanGuard pre-column (1.7 μm, 2.1 mm x 5 mm; Waters). Peptides were eluted into an Acquity UPLC BEH C18 analytical column (1.7 μm, 2.1 mm x 100 mm, Waters) with a linear gradient raising from 8 to 40% of Solvent B (0.23% formic acid in acetonitrile) at a flow rate of 40 μL/min and at 0 °C. Then, peptides went through electrospray ionization in positive mode and underwent MS analysis with ion mobility separation. To eliminate peptide carryover, the protease column was washed between each run of deuterated samples using 1.5 M Gu-HCl in 100 mM phosphate buffer (pH 2.5) and a run with a saw-tooth gradient was carried out for washing the chromatographic segments.

The peptide deuterium incorporation was calculated with DynamX 3.0 and the statistical analysis was performed based on an approach described earlier.^101^ Briefly, the threshold of significance difference in HDX was calculated based on the average standard deviation (SD) of peptide deuterium content for time points performed in triplicates, following Equation 1:

(Equation 1) $${SD}_{state}=\frac{\sqrt{\sum {{SD}_{i}}^{2}}}{N}$$

where N is the number of peptides considered, multiplied by the number of time points performed in triplicate. The pooled SD for the difference between the two states was subsequently calculated using Equation 2:

(Equation 2) $${SD}_{pool}=\sqrt{\left({{SD}_{stateA}}^{2}+{{SD}_{stateB}}^{2}\right)}$$

Based on the pooled SD, a CI at the significance level of 98% with a zero-centered average difference in deuterium content, considering a two-tailed distribution with two degrees of freedom (n = 3), was calculated by using Equation 3:

(Equation 3) $$CI=\pm 6.965\times \frac{{SD}_{pool}}{\sqrt{3}}$$

#### Biolayer interferometry

Measurements of S1 binding to immobilized TMEM106B^LD^ were performed with the Sartorius Octet R8 system in a buffer containing 150 mM NaCl and 20 mM Tris-HCl, pH 8.0, at 25 °C, and shaking at 1,000 rpm. TMEM106B^LD^ at ∼200 μg/mL was immobilized on NiNTA sensors for 10 min. The sensors were then moved to S1 solutions at varying concentrations and association measured for 3 min, followed by dissociation for 3-5 min. Experiments with both Belgium/GHB-03021 and Wuhan-Hu-1 S1 were performed at least three times. The results were analyzed using both kinetic and equilibrium approaches. The former used association phases to derive the observed rate (k_obs_) using a single exponential function, which allowed derivation of k_on_ and k_off_ from plots of k_obs_ versus S1 concentration. The data for equilibrium analysis were normalized by dividing by the maximum observable response to obtain fractional saturation as a function of S1 concentration.

The influence of ACE2 binding on the S1-TMEM106B interaction was measured by preincubating 6 μM S1 with 8 μM ACE2 for 10 min, from which five consecutive one to one dilutions with buffer were then obtained to give a dilution series. The measurements were then carried out as described above, flowing the S1-ACE2 complex over sensor-immobilized TMEM106B^LD^. The resulting data show no association phase, indicating a lack of interaction in the measured range of concentrations.

#### Cell-cell fusion assays

The coding sequence of *TMPRSS2* (Integrated DNA Technologies) was coupled to a hygromycin resistance gene via an IRES and driven by a cytomegalovirus promotor. The coding sequence for SARS-CoV-2-S was obtained from B. J. Bosch, Utrecht University, The Netherlands. To match the sequence of SARS-CoV-2/Belgium/GHB-03021/2020 passage 6, two deletions (68-76 and 676-680) and substitutions S813I and E484D were introduced. Those cDNA sequences were coupled with an IRES mNeonGreen-NES/PKI (Integrated DNA Technologies), a P2A blasticidin resistance gene and driven by a cytomegalovirus promotor. All cDNA sequences were inserted into the pLCKO plasmid which was a gift from Jason Moffat (Addgene plasmid #73311) in which the gRNA scaffold and the puromycin resistance gene were removed using the In-Fusion HD Kit (ST0345, Takara). For cell-cell fusion assays HEK293T cells were co-transfected with three plasmids encoding spike/mNeonGreen, TMPRSS2, and Luc, ACE2 or TMEM106B using Turbofectin 8.0 (OriGene TF81001) according to the manufacturer’s protocol. Cells were scanned at regular time intervals in an Incucyte S3. Using the Incucyte S3 analysis software, syncytia were identified by filtering for objects with a surface >1000 μm^2^ and their total surface area per image was calculated.

#### Simple Western analysis

For Simple Western analysis, cells were lysed in RIPA lysis buffer (Sigma) for 1 hour on ice. Whole cell lysates were cleared by centrifugation. Proteins were separated by size (12-230 kDa) and visualized on a Wes system (ProteinSimple, San Jose, CA, USA) with an anti-mouse IgG-HRP (042-205, Protein Simple) or anti-goat IgG-HRP antibody (043-522-2, Protein Simple) detecting the primary antibody against glyceraldehyde-3-phosphate dehydrogenase (GAPDH) (sc-47724, Santa Cruz Biotechnology, diluted 1:500) vinculin (Cell Signaling, catalog no. 13901, diluted 1:500) or anti-hACE2 (AF933, R&D systems, diluted 1:400), respectively. Protein signals were visualized and quantified with the Compass software, v6.1.0 (Protein Simple).

### Quantification and statistical analysis

Statistical analyses were performed with GraphPad Prism software. All analyses were performed at threshold alpha level of 0.05. The statistical details and specific statistical test used for each dataset is mentioned in the respective figure caption, as well as any data transformation that was applied. All measurements were taken from distinct samples.

## Data Availability

• The crystal structure of TMEM106B^LD^ and the cryo-EM maps of the spike-TMEM106B complex reported in this work are publicly available as of the date of publication. The corresponding Protein and EM Data Bank accession numbers are listed in the key resources table. The HDX data tables and deuterium uptake plots are added as Supplemental information. All other data reported in this paper will be shared by the lead contact upon request. • This paper does not report original code. • Any additional information required to reanalyze the data reported in this paper is available from the lead contact upon request.

## Supplemental information

Table S1. X-ray data collection and refinement statistics, related to Figure 3

Table S2. Oligonucleotides, gBlocks, and synthetic genes used in this study, related to STAR Methods

Table S3. Summary of HDX results and HDX data table, related to Figure 3

Data S1. HDX deuterium uptake plots, related to Figure 3

## References

[1] Drożdżal S., Rosik J., Lechowicz K., Machaj F., Szostak B., Przybyciński J., Lorzadeh S., Kotfis K., Ghavami S., Łos M.J. (2021). An update on drugs with therapeutic potential for SARS-CoV-2 (COVID-19) treatment. Drug Resist. Updat..

[2] Kuba K., Imai Y., Rao S., Gao H., Guo F., Guan B., Huan Y., Yang P., Zhang Y., Deng W. (2005). A crucial role of angiotensin converting enzyme 2 (ACE2) in SARS coronavirus-induced lung injury. Nat. Med..

[3] Yan R., Zhang Y., Li Y., Xia L., Guo Y., Zhou Q. (2020). Structural basis for the recognition of SARS-CoV-2 by full-length human ACE2. Science.

[4] Zhou P., Yang X.-L., Wang X.G., Hu B., Zhang L., Zhang W., Si H.R., Zhu Y., Li B., Huang C.L. (2020). A pneumonia outbreak associated with a new coronavirus of probable bat origin. Nature.

[5] Hoffmann M., Kleine-Weber H., Schroeder S., Krüger N., Herrler T., Erichsen S., Schiergens T.S., Herrler G., Wu N.H., Nitsche A. (2020). SARS-CoV-2 cell entry depends on ACE2 and TMPRSS2 and is blocked by a clinically proven protease inhibitor. Cell.

[6] Shang J., Wan Y., Luo C., Ye G., Geng Q., Auerbach A., Li F. (2020). Cell entry mechanisms of SARS-CoV-2. Proc. Natl. Acad. Sci. USA.

[7] Kumar N., Sharma S., Kumar R., Tripathi B.N., Barua S., Ly H., Rouse B.T. (2020). Host-directed antiviral therapy. Clin. Microbiol. Rev..

[8] Martinez J.P., Sasse F., Brönstrup M., Diez J., Meyerhans A. (2015). Antiviral drug discovery: broad-spectrum drugs from nature. Nat. Prod. Rep..

[9] Baggen J., Persoons L., Vanstreels E., Jansen S., Van Looveren D., Boeckx B., Geudens V., De Man J., Jochmans D., Wauters J. (2021). Genome-wide CRISPR screening identifies TMEM106B as a proviral host factor for SARS-CoV-2. Nat. Genet..

[10] Schneider W.M., Luna J.M., Hoffmann H.H., Sánchez-Rivera F.J., Leal A.A., Ashbrook A.W., Le Pen J., Ricardo-Lax I., Michailidis E., Peace A. (2021). Genome-scale identification of SARS-CoV-2 and pan-coronavirus Host Factor Networks. Cell.

[11] Zhang Q., Chen C.Z., Swaroop M., Xu M., Wang L., Lee J., Wang A.Q., Pradhan M., Hagen N., Chen L. (2020). Heparan sulfate assists SARS-CoV-2 in cell entry and can be targeted by approved drugs in vitro. Cell Discov..

[12] Wang C., Dinesh R.K., Qu Y., Rustagi A., Cousins H., Zengel J., Guo Y., Hall T., Beck A., Tso L. (2021).

[13] Wang R., Simoneau C.R., Kulsuptrakul J., Bouhaddou M., Travisano K.A., Hayashi J.M., Carlson-Stevermer J., James R., Richards C.M., Fozouni P. (2020). Genetic screens identify host factors for SARS-CoV-2 and common cold coronaviruses. Cell.

[14] Lang C.M., Fellerer K., Schwenk B.M., Kuhn P.H., Kremmer E., Edbauer D., Capell A., Haass C. (2012). Membrane orientation and subcellular localization of transmembrane protein 106B (TMEM106B), a major risk factor for frontotemporal lobar degeneration. J. Biol. Chem..

[15] Brady O.A., Zheng Y., Murphy K., Huang M., Hu F. (2013). The frontotemporal lobar degeneration risk factor, TMEM106B, regulates lysosomal morphology and function. Hum. Mol. Genet..

[16] Chen-Plotkin A.S., Unger T.L., Gallagher M.D., Bill E., Kwong L.K., Volpicelli-Daley L., Busch J.I., Akle S., Grossman M., Van Deerlin V. (2012). TMEM106B, the risk gene for frontotemporal dementia, is regulated by the microRNA-132/212 cluster and affects progranulin pathways. J. Neurosci..

[17] Uhlén M., Fagerberg L., Hallström B.M., Lindskog C., Oksvold P., Mardinoglu A., Sivertsson Å., Kampf C., Sjöstedt E., Asplund A. (2015). Proteomics. Tissue-based map of the human proteome. Science.

[18] Feng T., Lacrampe A., Hu F. (2021). Physiological and pathological functions of TMEM106B: a gene associated with brain aging and multiple brain disorders. Acta Neuropathol..

[19] Van Deerlin V.M., Sleiman P.M.A., Martinez-Lage M., Chen-Plotkin A., Wang L.S., Graff-Radford N.R., Dickson D.W., Rademakers R., Boeve B.F., Grossman M. (2010). Common variants at 7p21 are associated with frontotemporal lobar degeneration with TDP-43 inclusions. Nat. Genet..

[20] Chang A., Xiang X., Wang J., Lee C., Arakhamia T., Simjanoska M., Wang C., Carlomagno Y., Zhang G., Dhingra S. (2022). Homotypic fibrillization of TMEM106B across diverse neurodegenerative diseases. Cell.

[21] Schweighauser M., Arseni D., Bacioglu M., Huang M., Lövestam S., Shi Y., Yang Y., Zhang W., Kotecha A., Garringer H.J. (2022). Age-dependent formation of TMEM106B amyloid filaments in human brains. Nature.

[22] Jiang Y.X., Cao Q., Sawaya M.R., Abskharon R., Ge P., DeTure M., Dickson D.W., Fu J.Y., Ogorzalek Loo R.R., Loo J.A. (2022). Amyloid fibrils in disease FTLD-TDP are composed of TMEM106B not TDP-43. Nature.

[23] Kundu S.T., Grzeskowiak C.L., Fradette J.J., Gibson L.A., Rodriguez L.B., Creighton C.J., Scott K.L., Gibbons D.L. (2018). TMEM106B drives lung cancer metastasis by inducing TFEB-dependent lysosome synthesis and secretion of cathepsins. Nat. Commun..

[24] Stagi M., Klein Z.A., Gould T.J., Bewersdorf J., Strittmatter S.M. (2014). Lysosome size, motility and stress response regulated by fronto-temporal dementia modifier TMEM106B. Mol. Cell. Neurosci..

[25] Lüningschrör P., Werner G., Stroobants S., Kakuta S., Dombert B., Sinske D., Wanner R., Lüllmann-Rauch R., Wefers B., Wurst W. (2020). The FTLD risk factor TMEM106B regulates the transport of lysosomes at the axon initial segment of motoneurons. Cell Rep..

[26] Feng T., Mai S., Roscoe J.M., Sheng R.R., Ullah M., Zhang J., Katz I.I., Yu H., Xiong W., Hu F. (2020). Loss of TMEM 106B and PGRN leads to severe lysosomal abnormalities and neurodegeneration in mice. EMBO Rep..

[27] Klein Z.A., Takahashi H., Ma M., Stagi M., Zhou M., Lam T.T., Strittmatter S.M., Klein Z.A., Takahashi H., Ma M. (2017). Loss of TMEM106B ameliorates lysosomal and frontotemporal dementia-related phenotypes in progranulin-deficient mice. Neuron.

[28] Feng T., Sheng R.R., Solé-Domènech S., Ullah M., Zhou X., Mendoza C.S., Enriquez L.C.M., Katz I.I., Paushter D.H., Sullivan P.M. (2020). A role of the frontotemporal lobar degeneration risk factor TMEM106B in myelination. Brain.

[29] Levine T.P. (2022). TMEM106B in humans and Vac7 and Tag1 in yeast are predicted to be lipid transfer proteins. Proteins.

[30] Puray-Chavez M., LaPak K.M., Schrank T.P., Elliott J.L., Bhatt D.P., Agajanian M.J., Jasuja R., Lawson D.Q., Davis K., Rothlauf P.W. (2021). Systematic analysis of SARS-CoV-2 infection of an ACE2-negative human airway cell. Cell Rep..

[31] Hoffmann M., Sidarovich A., Arora P., Krüger N., Nehlmeier I., Kempf A., Graichen L., Winkler M.S., Niemeyer D., Goffinet C. (2022). Evidence for an ACE2-independent entry pathway that can protect from neutralization by an antibody used for COVID-19 therapy. mBio.

[32] Niemeyer D., Stenzel S., Veith T., Schroeder S., Friedmann K., Weege F., Trimpert J., Heinze J., Richter A., Jansen J. (2022). SARS-CoV-2 variant Alpha has a spike-dependent replication advantage over the ancestral B.1 strain in human cells with low ACE2 expression. PLoS Biol..

[33] Magazine N., Zhang T., Wu Y., McGee M.C., Veggiani G., Huang W. (2022). Mutations and evolution of the SARS-CoV-2 spike protein. Viruses.

[34] Zhu Y., Feng F., Hu G., Wang Y., Yu Y., Zhu Y., Xu W., Cai X., Sun Z., Han W. (2021). A genome-wide CRISPR screen identifies host factors that regulate SARS-CoV-2 entry. Nat. Commun..

[35] Fraser J.S., Yu Z., Maxwell K.L., Davidson A.R. (2006). Ig-like domains on bacteriophages: A tale of promiscuity and deceit. J. Mol. Biol..

[36] Hsieh C.L., Goldsmith J.A., Schaub J.M., Divenere A.M., Kuo H.C., Javanmardi K., Le K.C., Wrapp D., Lee A.G., Liu Y. (2020). Structure-based design of prefusion-stabilized SARS-CoV-2 spikes. Science.

[37] Li F., Li W., Farzan M., Harrison S.C. (2005). Structure of SARS coronavirus spike receptor-binding domain complexed with receptor. Science.

[38] Shang J., Ye G., Shi K., Wan Y., Luo C., Aihara H., Geng Q., Auerbach A., Li F. (2020). Structural basis of receptor recognition by SARS-CoV-2. Nature.

[39] Wang Q., Zhang Y., Wu L., Niu S., Song C., Zhang Z., Lu G., Qiao C., Hu Y., Yuen K.Y. (2020). Structural and functional basis of SARS-CoV-2 entry by using human ACE2. Cell.

[40] Calvaresi V., Wrobel A.G., Toporowska J., Hammerschmid D., Doores K.J., Bradshaw R.T., Parsons R.B., Benton D.J., Roustan C., Reading E. (2023). Structural dynamics in the evolution of SARS-CoV-2 spike glycoprotein. Nat. Commun..

[41] Wrapp D., Wang N., Corbett K.S., Goldsmith J.A., Hsieh C.L., Abiona O., Graham B.S., McLellan J.S. (2020). Cryo-EM structure of the 2019-nCoV spike in the prefusion conformation. Science.

[42] Wrobel A.G., Benton D.J., Xu P., Roustan C., Martin S.R., Rosenthal P.B., Skehel J.J., Gamblin S.J. (2020). SARS-CoV-2 and bat RaTG13 spike glycoprotein structures inform on virus evolution and furin-cleavage effects. Nat. Struct. Mol. Biol..

[43] Barton M.I., Macgowan S.A., Kutuzov M.A., Dushek O., Barton G.J., van der Merwe P.A. (2021). Effects of common mutations in the sars-cov-2 spike rbd and its ligand the human ace2 receptor on binding affinity and kinetics. eLife.

[44] Peng R., Wu L.A., Wang Q., Qi J., Gao G.F. (2021). Cell entry by SARS-CoV-2. Trends Biochem. Sci..

[45] Baggen J., Vanstreels E., Jansen S., Daelemans D. (2021). Cellular host factors for SARS-CoV-2 infection. Nat. Microbiol..

[46] Daly J.L., Simonetti B., Klein K., Chen K.-E., Williamson M.K., Antón-Plágaro C., Shoemark D.K., Simón-Gracia L., Bauer M., Hollandi R. (2020). Neuropilin-1 is a host factor for SARS-CoV-2 infection. Science.

[47] Cantuti-Castelvetri L., Ojha R., Pedro L.D., Djannatian M., Franz J., Kuivanen S., van der Meer F., Kallio K., Kaya T., Anastasina M. (2020). Neuropilin-1 facilitates SARS-CoV-2 cell entry and infectivity. Science.

[48] Clausen T.M., Sandoval D.R., Spliid C.B., Pihl J., Painter C.D., Thacker B.E., Glass C.A., Narayanan A., Majowicz S.A., Zhang Y. (2020). SARS-CoV-2 infection depends on cellular heparan sulfate and ACE2. Cell.

[49] Nguyen L., McCord K.A., Bui D.T., Bouwman K.M., Kitova E.N., Elaish M., Kumawat D., Daskhan G.C., Tomris I., Han L. (2022). Sialic acid-containing glycolipids mediate binding and viral entry of SARS-CoV-2. Nat. Chem. Biol..

[50] Schurink B., Roos E., Radonic T., Barbe E., Bouman C.S.C., de Boer H.H., de Bree G.J., Bulle E.B., Aronica E.M., Florquin S. (2020). Viral presence and immunopathology in patients with lethal COVID-19: a prospective autopsy cohort study. Lancet Microbe.

[51] Puelles V.G., Lütgehetmann M., Lindenmeyer M.T., Sperhake J.P., Wong M.N., Allweiss L., Chilla S., Heinemann A., Wanner N., Liu S. (2020). Multiorgan and renal tropism of SARS-CoV-2. N. Engl. J. Med..

[52] Liu J., Li Y., Liu Q., Yao Q., Wang X., Zhang H., Chen R., Ren L., Min J., Deng F. (2021). SARS-CoV-2 cell tropism and multiorgan infection. Cell Discov..

[53] Hikmet F., Méar L., Edvinsson Å., Micke P., Uhlén M., Lindskog C. (2020). The protein expression profile of ACE2 in human tissues. Mol. Syst. Biol..

[54] Jackson C.B., Farzan M., Chen B., Choe H. (2022). Mechanisms of SARS-CoV-2 entry into cells. Nat. Rev. Mol. Cell Biol..

[55] Benton D.J., Wrobel A.G., Xu P., Roustan C., Martin S.R., Rosenthal P.B., Skehel J.J., Gamblin S.J. (2020). Receptor binding and priming of the spike protein of SARS-CoV-2 for membrane fusion. Nature.

[56] Cai Y., Zhang J., Xiao T., Peng H., Sterling S.M., Walsh R.M., Rawson S., Rits-Volloch S., Chen B. (2020). Distinct conformational states of SARS-CoV-2 spike protein. Science.

[57] Wrobel A.G., Benton D.J., Roustan C., Borg A., Hussain S., Martin S.R., Rosenthal P.B., Skehel J.J., Gamblin S.J. (2022). Evolution of the SARS-CoV-2 spike protein in the human host. Nat. Commun..

[58] Wang H., Shi Y., Song J., Qi J., Lu G., Yan J., Gao G.F. (2016). Ebola viral glycoprotein bound to its endosomal receptor Niemann-Pick C1. Cell.

[59] Quiros-Roldan E., Amadasi S., Zanella I., Degli Antoni M.D., Storti S., Tiecco G., Castelli F. (2021). Monoclonal antibodies against sars-cov-2: current scenario and future perspectives. Pharmaceuticals.

[60] Planas D., Saunders N., Maes P., Guivel-Benhassine F., Planchais C., Buchrieser J., Bolland W.H., Porrot F., Staropoli I., Lemoine F. (2022). Considerable escape of SARS-CoV-2 Omicron to antibody neutralization. Nature.

[61] Dejnirattisai W., Huo J., Zhou D., Zahradník J., Supasa P., Liu C., Duyvesteyn H.M.E., Ginn H.M., Mentzer A.J., Tuekprakhon A. (2022). SARS-CoV-2 Omicron-B.1.1.529 leads to widespread escape from neutralizing antibody responses. Cell.

[62] Feng T., Luan L., Katz I.I., Ullah M., Van Deerlin V.M., Trojanowski J.Q., Lee E.B., Hu F. (2022). TMEM106B deficiency impairs cerebellar myelination and synaptic integrity with Purkinje cell loss. Acta Neuropathol. Commun..

[63] Holm L. (2022). Dali server: structural unification of protein families. Nucleic Acids Res..

[64] Chua R.L., Lukassen S., Trump S., Hennig B.P., Wendisch D., Pott F., Debnath O., Thürmann L., Kurth F., Völker M.T. (2020). COVID-19 severity correlates with airway epithelium–immune cell interactions identified by single-cell analysis. Nat. Biotechnol..

[65] Bost P., Giladi A., Liu Y., Bendjelal Y., Xu G., David E., Blecher-Gonen R., Cohen M., Medaglia C., Li H. (2020). Host-Viral Infection Maps Reveal Signatures of Severe COVID-19 Patients. Cell.

[66] Boudewijns R., Thibaut H.J., Kaptein S.J.F., Li R., Vergote V., Seldeslachts L., Van Weyenbergh J., De Keyzer C., Bervoets L., Sharma S. (2020). STAT2 signaling restricts viral dissemination but drives severe pneumonia in SARS-CoV-2 infected hamsters. Nat. Commun..

[67] Abdelnabi R., Boudewijns R., Foo C.S., Seldeslachts L., Sanchez-Felipe L., Zhang X., Delang L., Maes P., Kaptein S.J.F., Weynand B. (2021). Comparing infectivity and virulence of emerging SARS-CoV-2 variants in Syrian hamsters. EBiomedicine.

[68] Abdelnabi R., Foo C.S., Zhang X., Lemmens V., Maes P., Slechten B., Raymenants J., André E., Weynand B., Dallmeier K. (2022). The omicron (B.1.1.529) SARS-CoV-2 variant of concern does not readily infect Syrian hamsters. Antiviral Res..

[69] Hart T., Chandrashekhar M., Aregger M., Steinhart Z., Brown K.R., MacLeod G., Mis M., Zimmermann M., Fradet-Turcotte A., Sun S. (2015). High-resolution CRISPR screens reveal fitness genes and genotype-specific cancer liabilities. Cell.

[70] Parik S., Fernández-García J., Lodi F., De Vlaminck K., Derweduwe M., De Vleeschouwer S., Sciot R., Geens W., Weng L., Bosisio F.M. (2022). GBM tumors are heterogeneous in their fatty acid metabolism and modulating fatty acid metabolism sensitizes cancer cells derived from recurring GBM tumors to temozolomide. Front. Oncol..

[71] Vijgen L., Keyaerts E., Moës E., Maes P., Duson G., Van Ranst M. (2005). Development of one-step, real-time, quantitative reverse transcriptase PCR assays for absolute quantitation of human coronaviruses OC43 and 229E. J. Clin. Microbiol..

[72] Sanjana N.E., Shalem O., Zhang F. (2014). Improved vectors and genome-wide libraries for CRISPR screening. Nat. Methods.

[73] Li W., Moore M.J., Vasilieva N., Sui J., Wong S.K., Berne M.A., Somasundaran M., Sullivan J.L., Luzuriaga K., Greenough T.C. (2003). Angiotensin-converting enzyme 2 is a functional receptor for the SARS coronavirus. Nature.

[74] Niwa H., Yamamura K., Miyazaki J. (1991). Efficient selection for high-expression transfectants with a novel eukaryotic vector. Gene.

[75] Rosenthal A., Brown E., Schwabe T., Yee A., Rhinn H. (2019).

[76] Rosa A., Pye V.E., Graham C., Muir L., Seow J., Ng K.W., Cook N.J., Rees-Spear C., Parker E., dos Santos M.S. (2021). SARS-CoV-2 can recruit a heme metabolite to evade antibody immunity. Sci. Adv..

[77] Wrobel A.G., Benton D.J., Xu P., Calder L.J., Borg A., Roustan C., Martin S.R., Rosenthal P.B., Skehel J.J., Gamblin S.J. (2021). Structure and binding properties of Pangolin-CoV spike glycoprotein inform the evolution of SARS-CoV-2. Nat. Commun..

[78] Stirling D.R., Swain-Bowden M.J., Lucas A.M., Carpenter A.E., Cimini B.A., Goodman A. (2021). CellProfiler 4: improvements in speed, utility and usability. BMC Bioinformatics.

[79] Winter G., Waterman D.G., Parkhurst J.M., Brewster A.S., Gildea R.J., Gerstel M., Fuentes-Montero L., Vollmar M., Michels-Clark T., Young I.D. (2018). DIALS: implementation and evaluation of a new integration package. Acta Crystallogr. D Struct. Biol..

[80] Winter G., Lobley C.M.C., Prince S.M. (2013). Decision making in xia2. Acta Crystallogr. D Biol. Crystallogr..

[81] Liebschner D., Afonine P.V., Baker M.L., Bunkóczi G., Chen V.B., Croll T.I., Hintze B., Hung L.W., Jain S., McCoy A.J. (2019). Macromolecular structure determination using X-rays, neutrons and electrons: recent developments in Phenix. Acta Crystallogr. D Struct. Biol..

[82] McCoy A.J., Grosse-Kunstleve R.W., Adams P.D., Winn M.D., Storoni L.C., Read R.J. (2007). Phaser crystallographic software. J. Appl. Crystallogr..

[83] Chen V.B., Arendall W.B., Headd J.J., Keedy D.A., Immormino R.M., Kapral G.J., Murray L.W., Richardson J.S., Richardson D.C. (2010). MolProbity: all-atom structure validation for macromolecular crystallography. Acta Crystallogr. D Biol. Crystallogr..

[84] Jumper J., Evans R., Pritzel A., Green T., Figurnov M., Ronneberger O., Tunyasuvunakool K., Bates R., Žídek A., Potapenko A. (2021). Highly accurate protein structure prediction with AlphaFold. Nature.

[85] Emsley P., Cowtan K. (2004). Coot: model-building tools for molecular graphics. Acta Crystallogr. D Biol. Crystallogr..

[86] Zheng S.Q., Palovcak E., Armache J.-P., Verba K.A., Cheng Y., Agard D.A. (2017). MotionCor2: anisotropic correction of beam-induced motion for improved cryo-electron microscopy. Nat. Methods.

[87] Zhang K. (2016). Gctf: real-time CTF determination and correction. J. Struct. Biol..

[88] Wagner T., Merino F., Stabrin M., Moriya T., Antoni C., Apelbaum A., Hagel P., Sitsel O., Raisch T., Prumbaum D. (2019). SPHIRE-crYOLO is a fast and accurate fully automated particle picker for cryo-EM. Commun. Biol..

[89] Punjani A., Rubinstein J.L., Fleet D.J., Brubaker M.A. (2017). cryoSPARC: algorithms for rapid unsupervised cryo-EM structure determination. Nat. Methods.

[90] Bepler T., Morin A., Rapp M., Brasch J., Shapiro L., Noble A.J., Berger B. (2019). Positive-unlabeled convolutional neural networks for particle picking in cryo-electron micrographs. Nat. Methods.

[91] Sanchez-Garcia R., Segura J., Maluenda D., Sorzano C.O.S., Carazo J.M. (2020). MicrographCleaner: A python package for cryo-EM micrograph cleaning using deep learning. J. Struct. Biol..

[92] Scheres S.H.W. (2020). Amyloid structure determination in RELION-3.1.. Acta Crystallogr. D Struct. Biol..

[93] Kimanius D., Dong L., Sharov G., Nakane T., Scheres S.H.W. (2021). New tools for automated cryo-EM single-particle analysis in RELION-4.0.. Biochem. J..

[94] Pettersen E.F., Goddard T.D., Huang C.C., Couch G.S., Greenblatt D.M., Meng E.C., Ferrin T.E. (2004). UCSF Chimera - A visualization system for exploratory research and analysis. J. Comput. Chem..

[95] Sanchez-Felipe L., Vercruysse T., Sharma S., Ma J., Lemmens V., Van Looveren D., Javarappa M.P.A., Boudewijns R., Malengier-Devlies B., Liesenborghs L. (2021). A single-dose live-attenuated YF17D-vectored SARS-CoV-2 vaccine candidate. Nature.

[96] Whitt M.A. (2010). Generation of VSV pseudotypes using recombinant ΔG-VSV for studies on virus entry, identification of entry inhibitors, and immune responses to vaccines. J. Virol. Methods.

[97] Afonine P.V., Grosse-Kunstleve R.W., Echols N., Headd J.J., Moriarty N.W., Mustyakimov M., Terwilliger T.C., Urzhumtsev A., Zwart P.H., Adams P.D. (2012). Towards automated crystallographic structure refinement with phenix.refine. Acta Crystallogr. D Biol. Crystallogr..

[98] Rosenthal P.B., Henderson R. (2003). Optimal determination of particle orientation, absolute hand, and contrast loss in single-particle electron cryomicroscopy. J. Mol. Biol..

[99] Scheres S.H.W., Chen S. (2012). Prevention of overfitting in cryo-EM structure determination. Nat. Methods.

[100] Hamuro Y., Coales S.J. (2022). Hydrogen/deuterium exchange mass spectrometry for weak binders. J. Am. Soc. Mass Spectrom..

[101] Houde D., Berkowitz S.A., Engen J.R. (2011). The utility of hydrogen/deuterium exchange mass spectrometry in biopharmaceutical comparability studies. J. Pharm. Sci..

