# Supplementary material for: TMEM106B is a receptor mediating ACE2-independent SARS-CoV-2 cell entry
Source: Cell. 2023 Aug 3;186(16):3427–3442.e22. doi: 10.1016/j.cell.2023.06.005 (PMC10409496; doi:10.1016/j.cell.2023.06.005)
Supplement: Table S1. X-ray data collection and refinement statistics, related to Figure 3 [file mmc1.pdf]

Cell, Volume *186*

Supplemental information

TMEM106B is a receptor mediating

ACE2-independent SARS-CoV-2 cell entry

Jim Baggen, Maarten Jacquemyn, Leentje Persoons, Els Vanstreels, Valerie E. Pye, Antoni G. Wrobel, Valeria Calvaresi, Stephen R. Martin, Chloë Roustan, Nora B. Cronin, Eamonn Reading, Hendrik Jan Thibaut, Thomas Vercruysse, Piet Maes, Frederik De Smet, Angie Yee, Toey Nivitchanyong, Marina Roell, Natalia Franco-Hernandez, Herve Rhinn, Alusha Andre Mamchak, Maxime Ah Young-Chapon, Eric Brown, Peter Cherepanov, and Dirk Daelemans

Table S1. X-ray data collection and refinement statistics. Related to Figure 3.

	**TMEM106B^{LD}**
**Data collection:**	
**Wavelength (Å)**	1.000
**Space group**	$P4_12_12$
**Unit cell parameters**	
a, b, c **(Å)**	52.4, 52.4, 132.9
**α, β, γ (°)**	90, 90, 90
**Number of crystals used**	1
**Resolution (Å)**	48.76-2.59 (2.64-2.59)
**Number of reflections**	
**measured**	157,094 (7,778)
**unique**	6,291 (301)
**Completeness (%)**	100 (100)
**Multiplicity**	25.0 (25.8)
**$\langle I/\sigma(I)\rangle$**	13.5 (0.7)
**R_{merge} (%)**	0.187 (3.804)
**R_{pim} (%)**	0.038 (0.756)
**CC 1/2**	0.999 (0.371)
**Refinement statistics:**	
**Resolution range (Å)**	48.76-2.59 (3.26-2.59)
**Number of reflections**	
**total**	6,244 (3030)
**free**	299 (137)
**R_{work}/R_{free}**	0.2343/2426
**Number of atoms**	
**total**	1,246
**protein**	1,185
**ligands**	56
**solvent**	5
**R.m.s. deviations from ideal**	
**bond lengths (Å)**	0.004
**bond angles (°)**	0.62
**Average B-factor (Å^2)**	75.38
**Clash Score** [b]	5.26
**Favored Rotamers** [b]	96.32
**Ramachandran plot (%)** [b]	
**favored**	97.24
**disallowed**	0

[a] Values in parentheses correspond to the highest resolution bin.

[b] Analyzed using MolProbity (http://molprobity.biochem.duke.edu/).
