# Supplementary figures and images for: TMEM106B is a receptor mediating ACE2-independent SARS-CoV-2 cell entry
Source: Cell. 2023 Aug 3;186(16):3427–3442.e22. doi: 10.1016/j.cell.2023.06.005 (PMC10409496; doi:10.1016/j.cell.2023.06.005)

**Data S1. HDX deuterium uptake plots. Related to Figure 3**

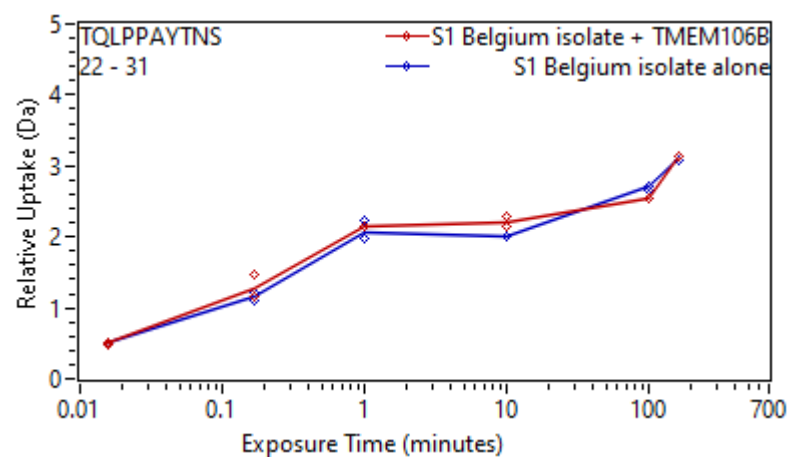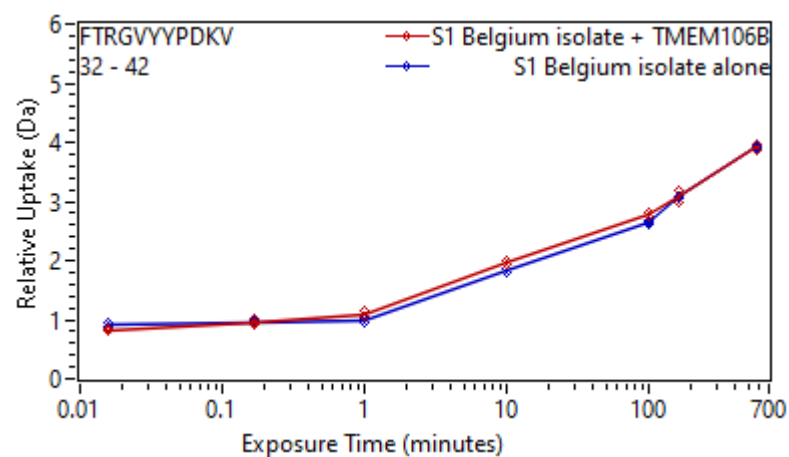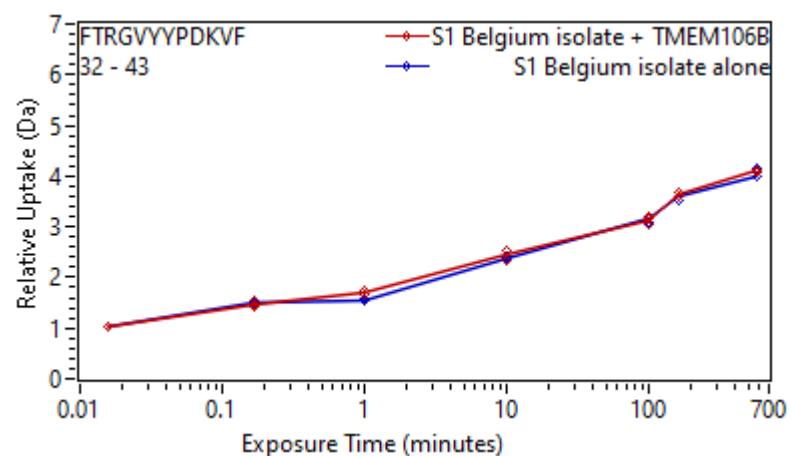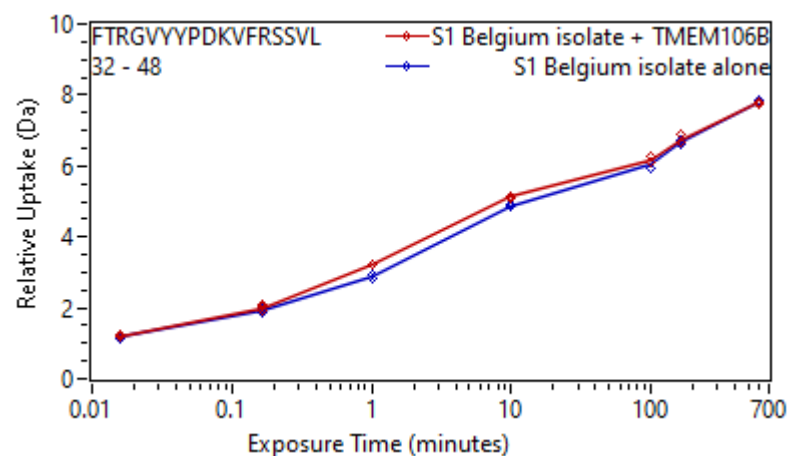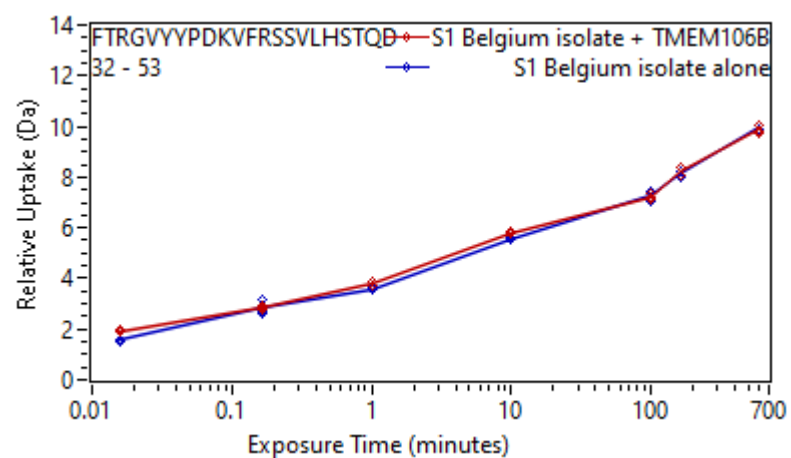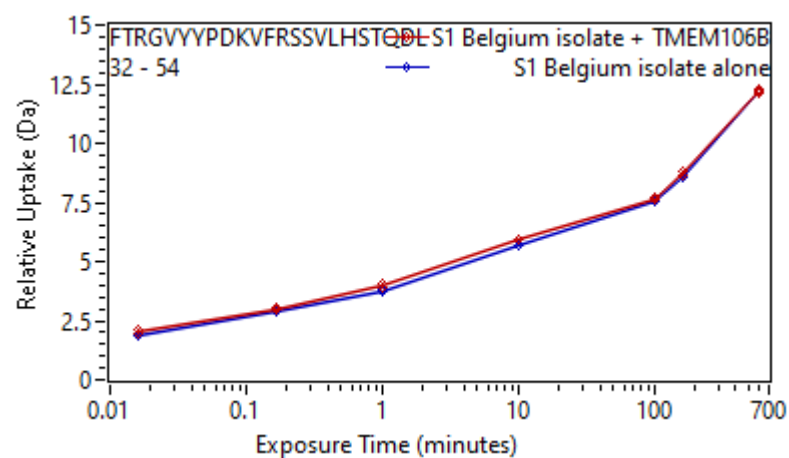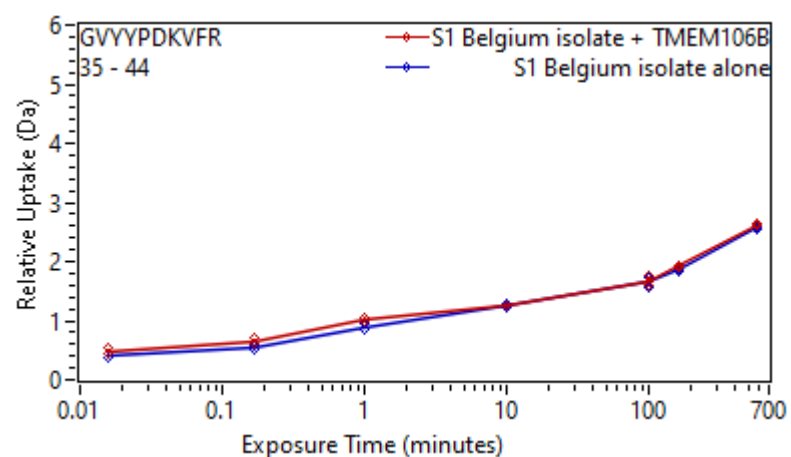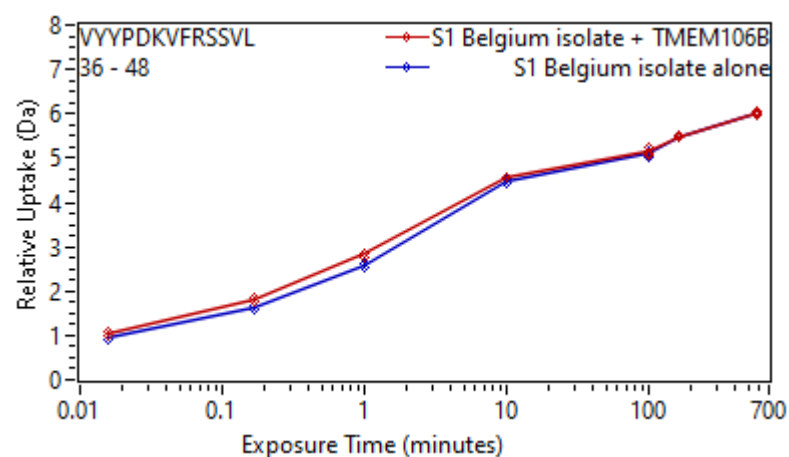

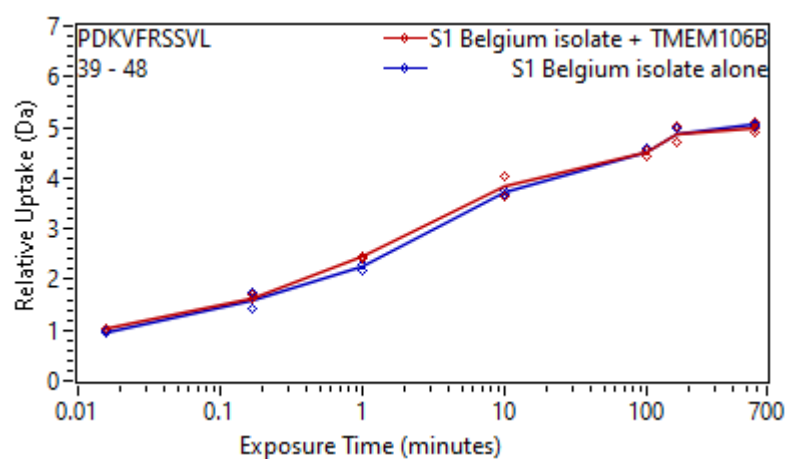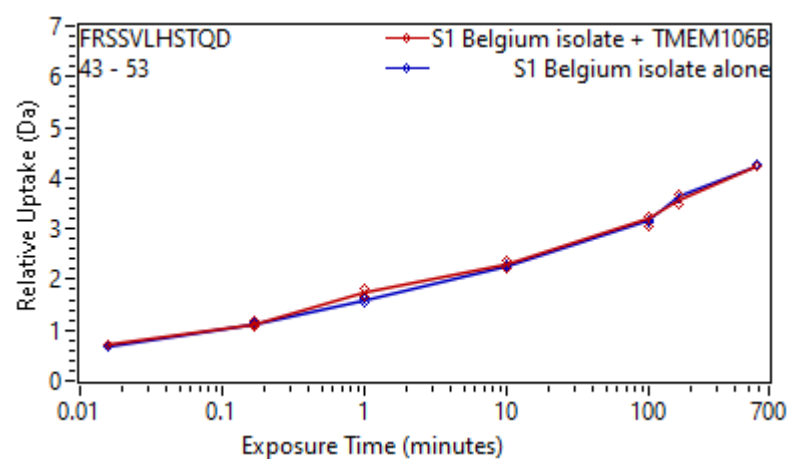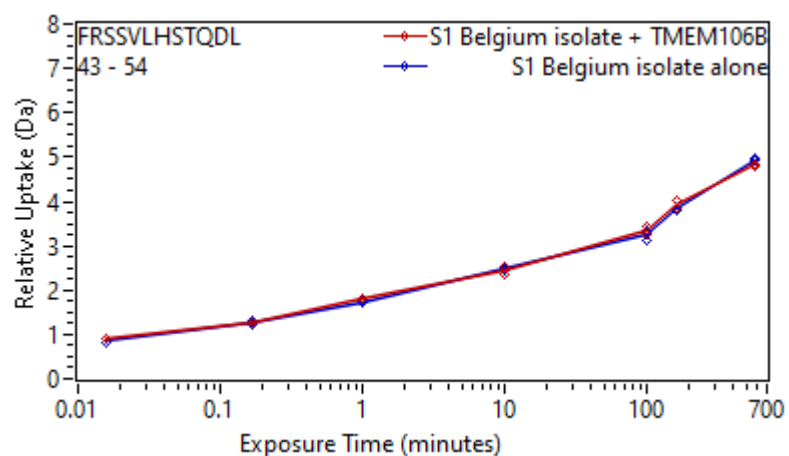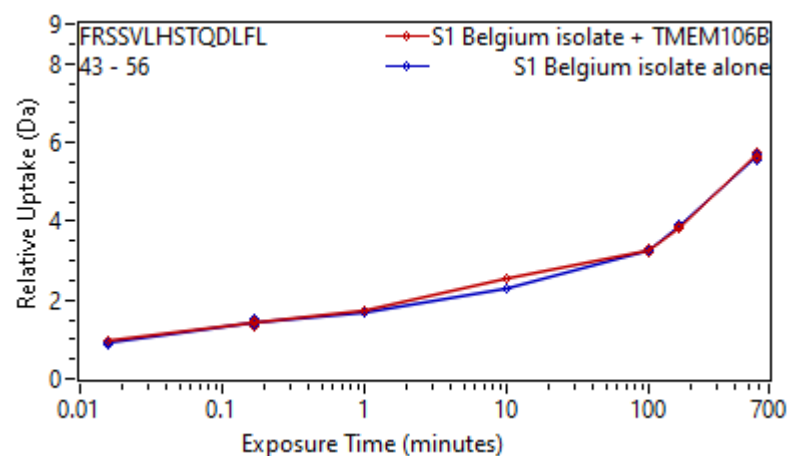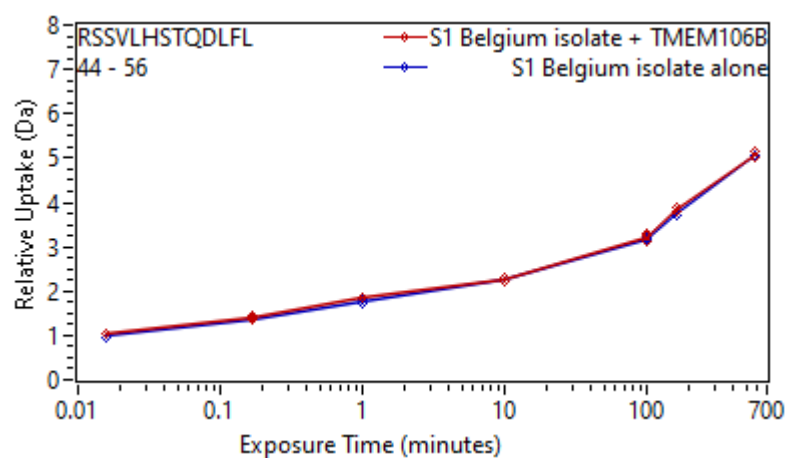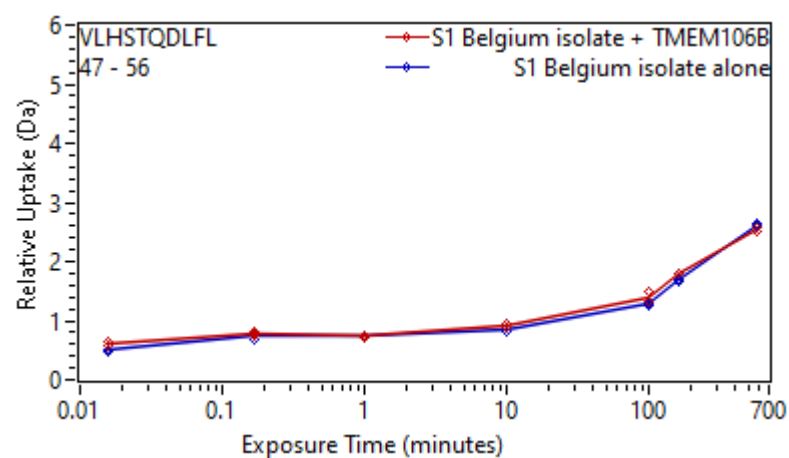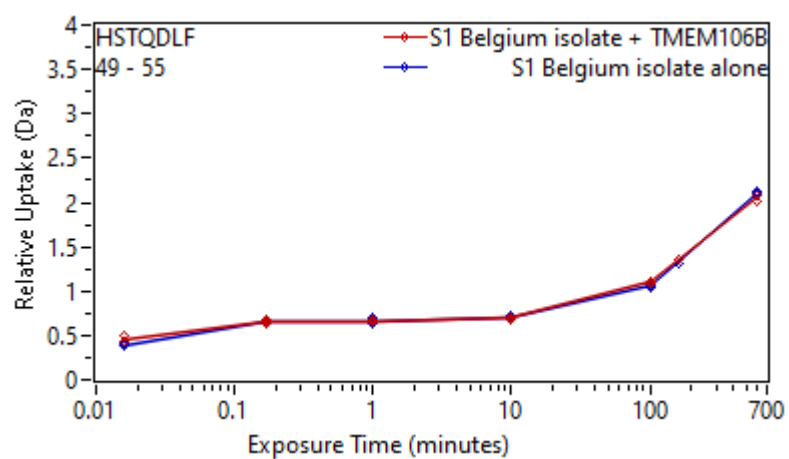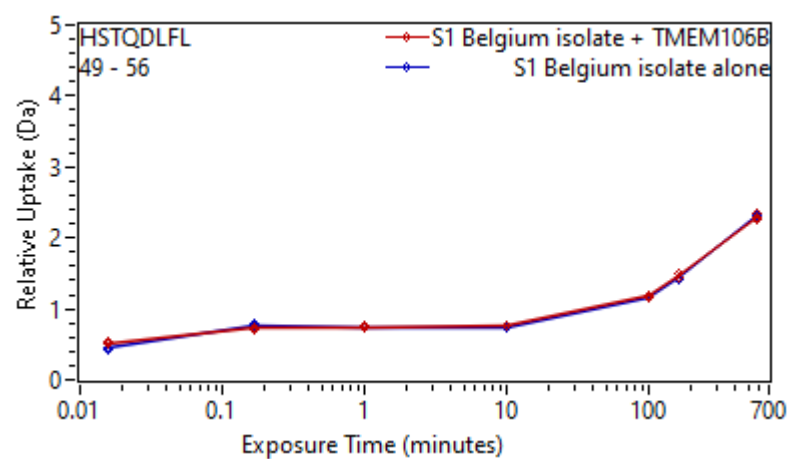

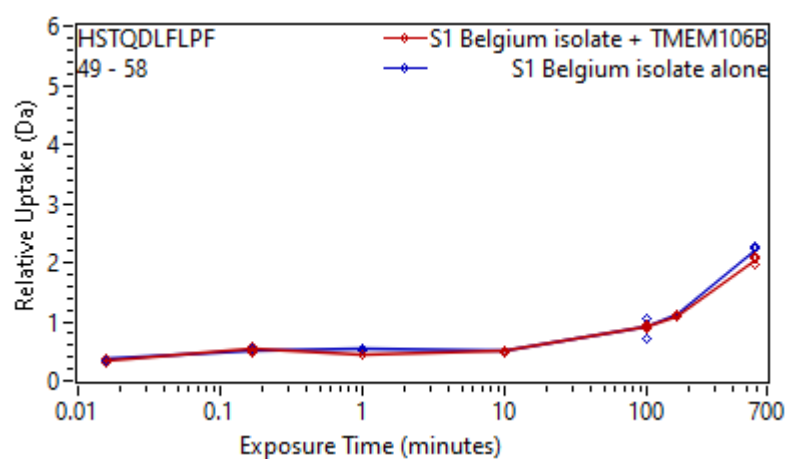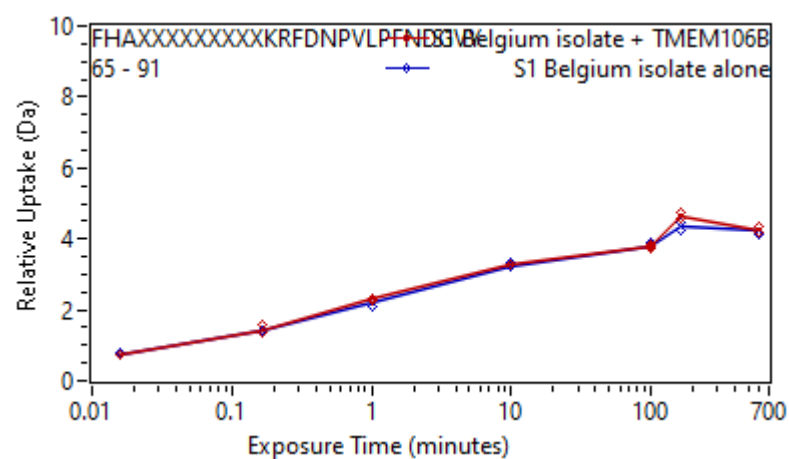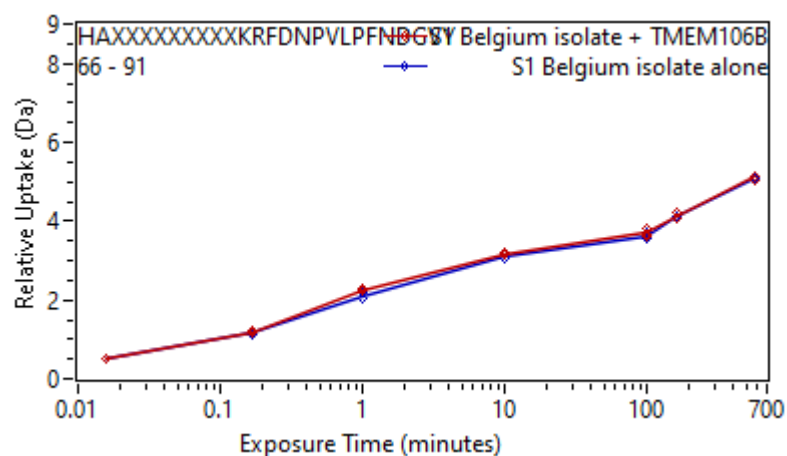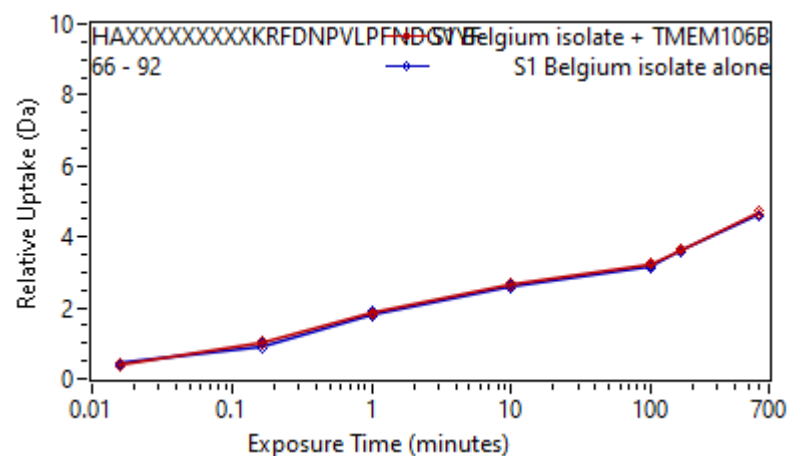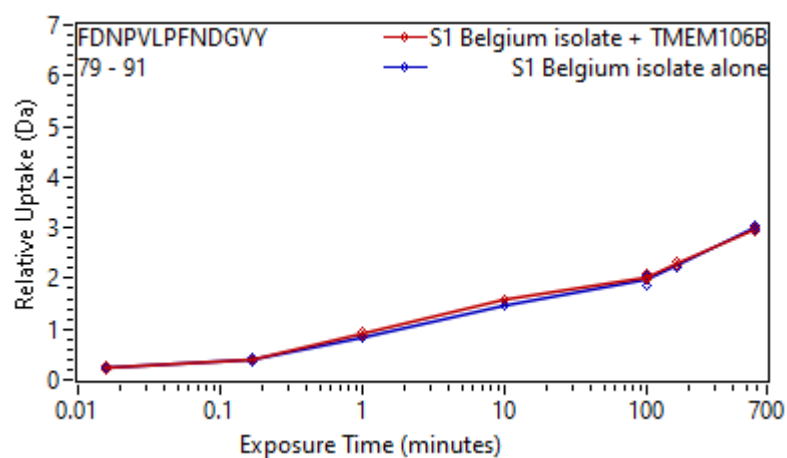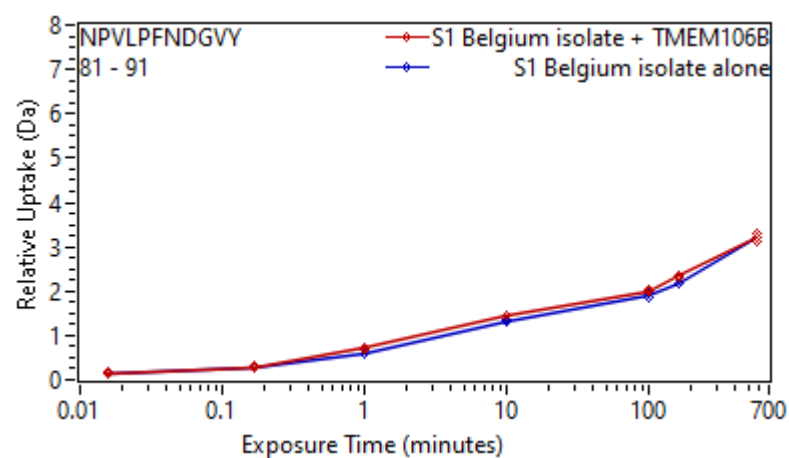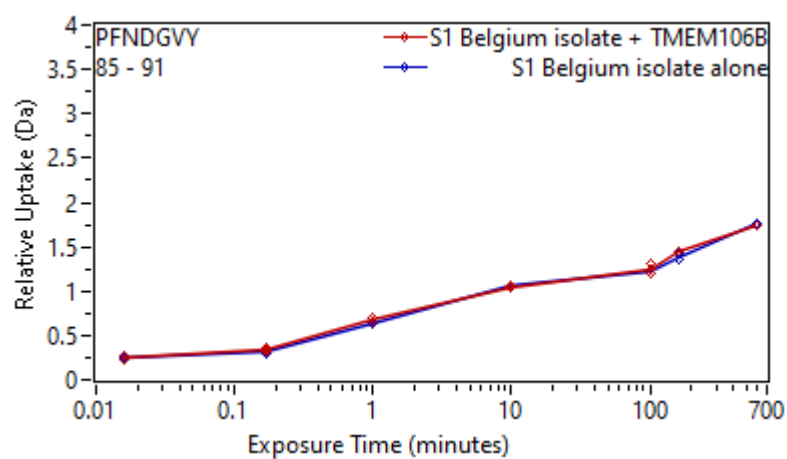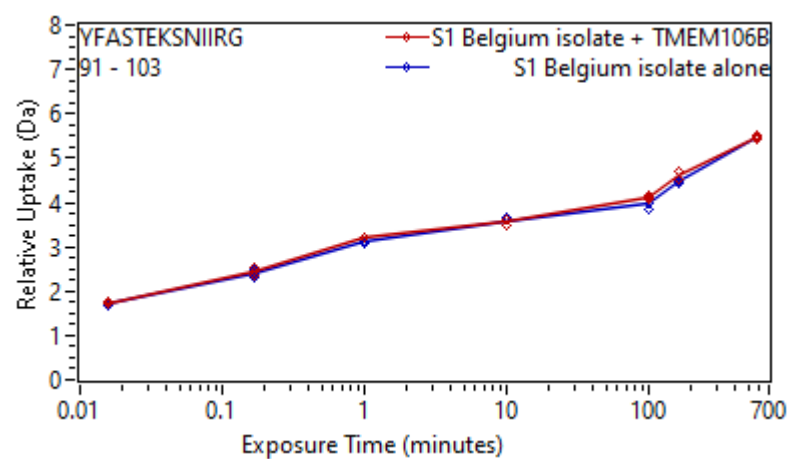

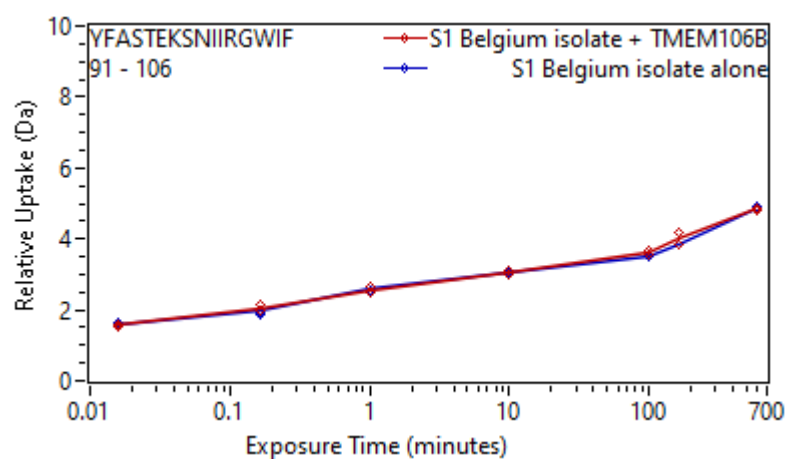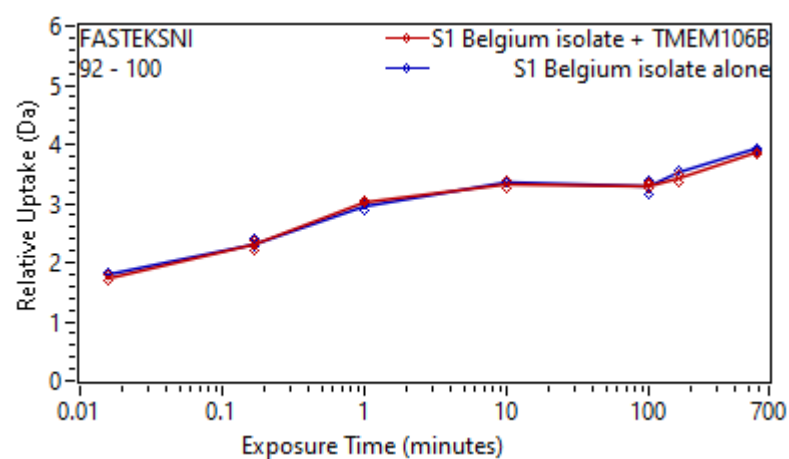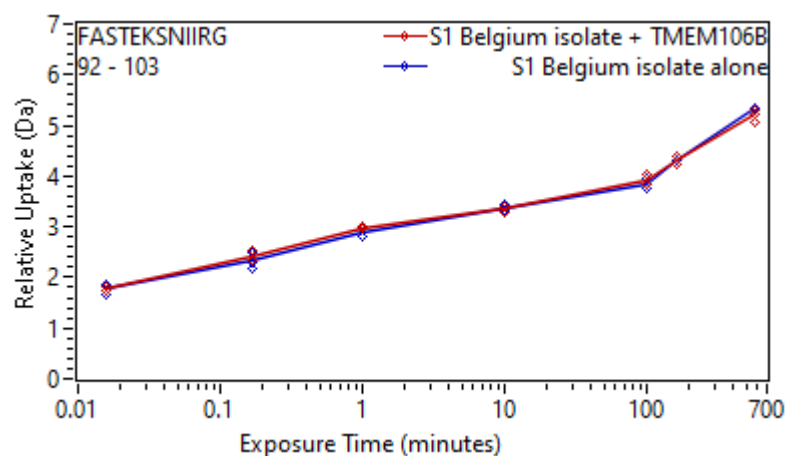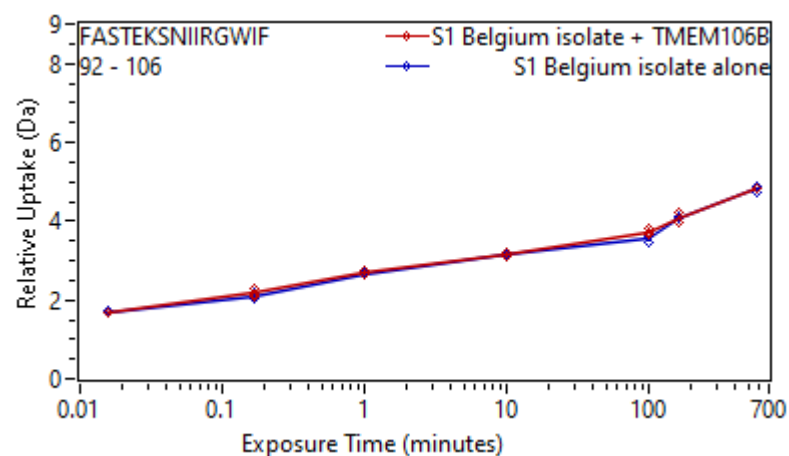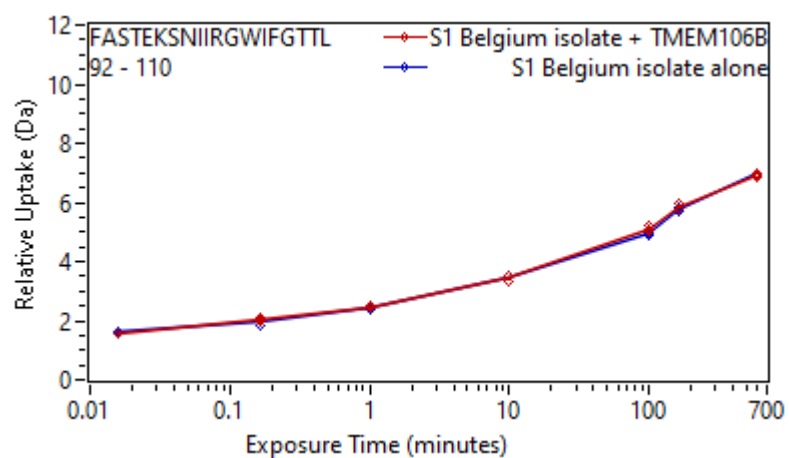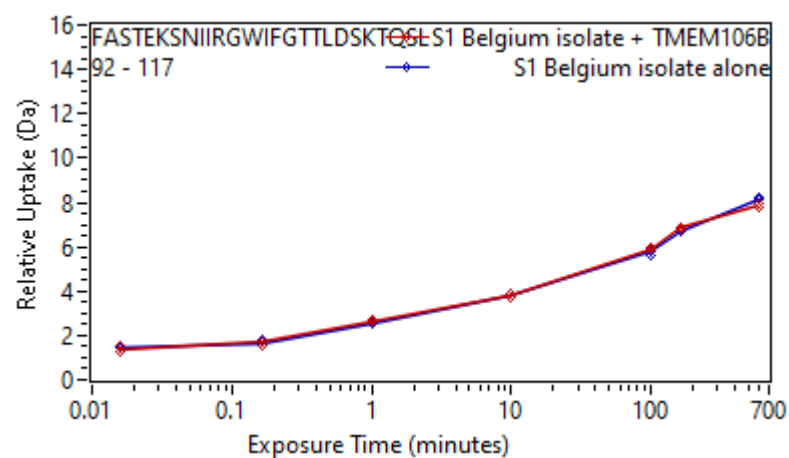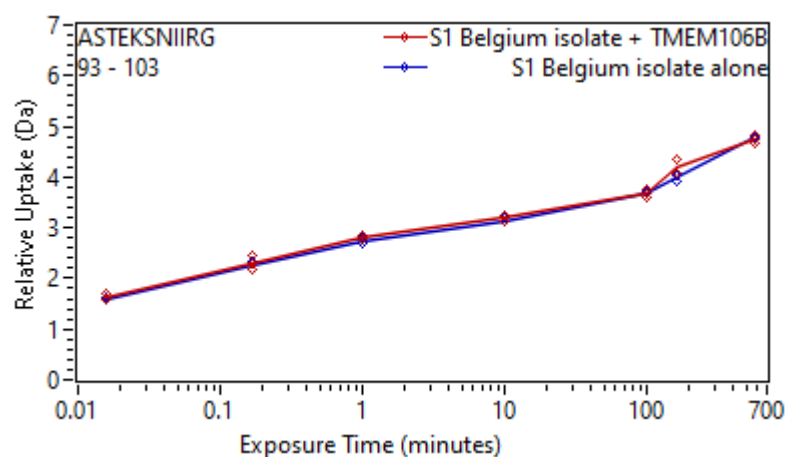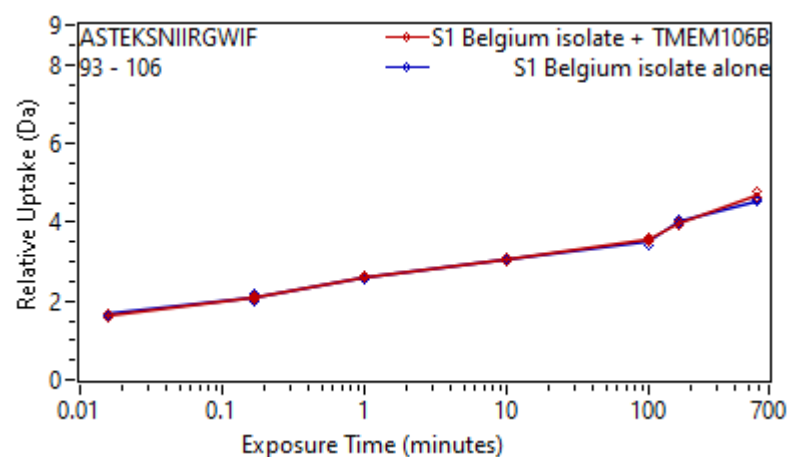

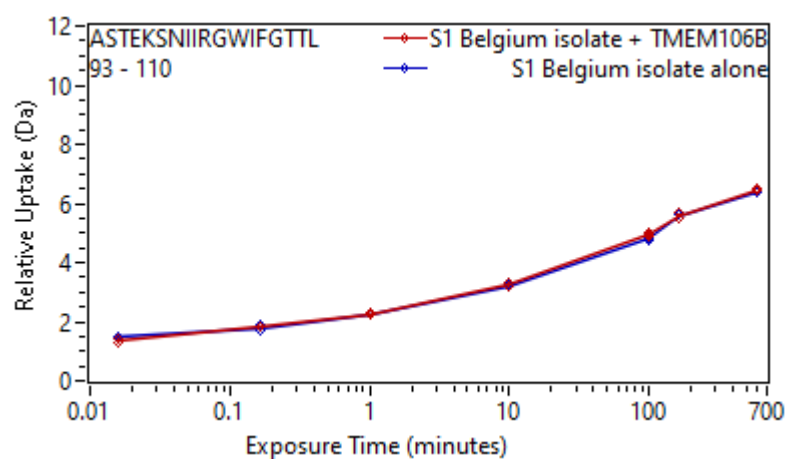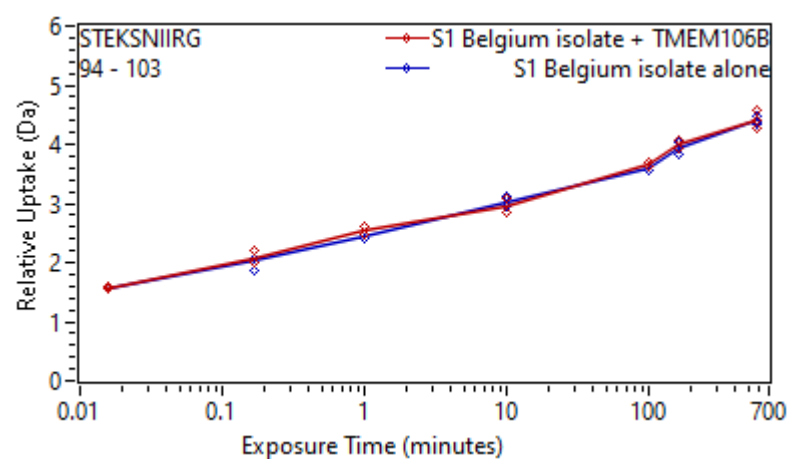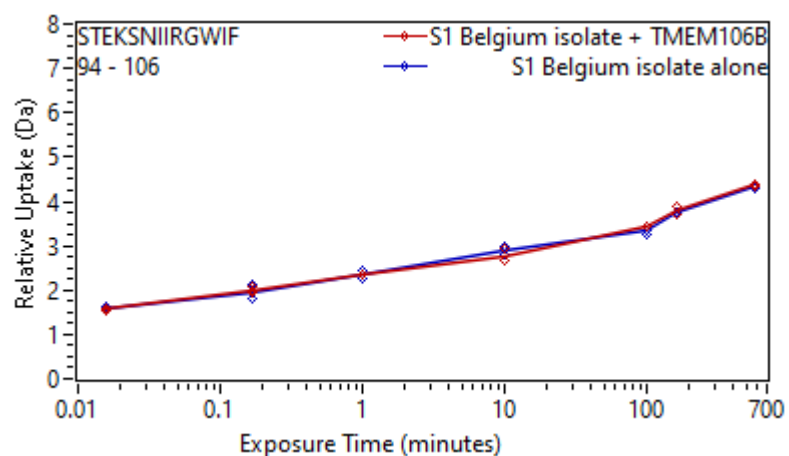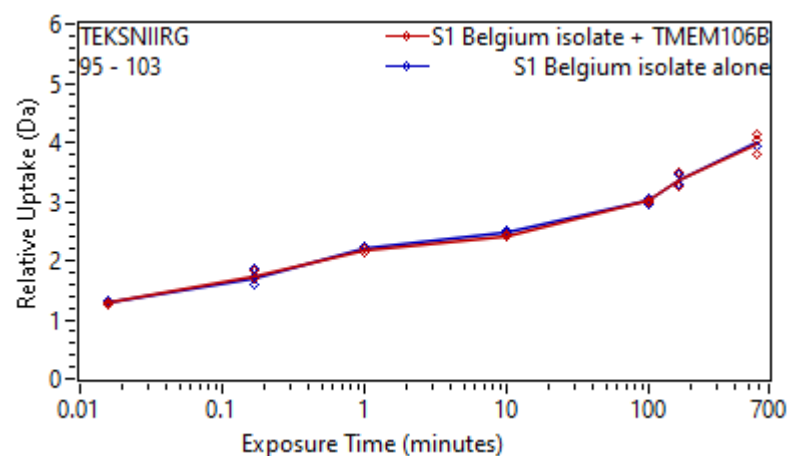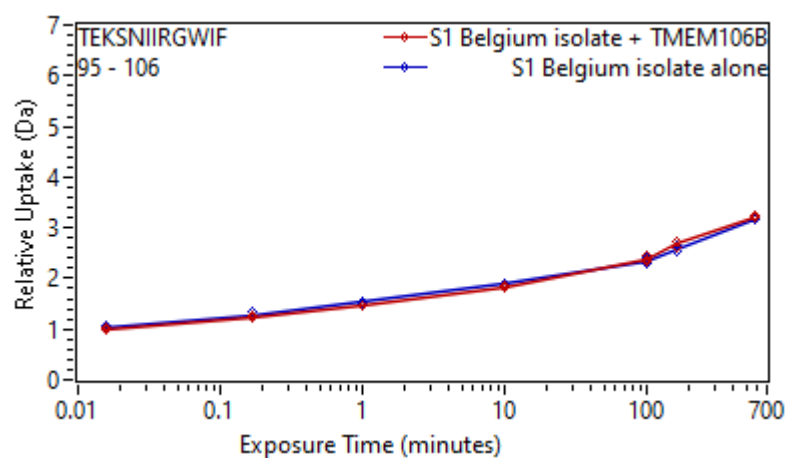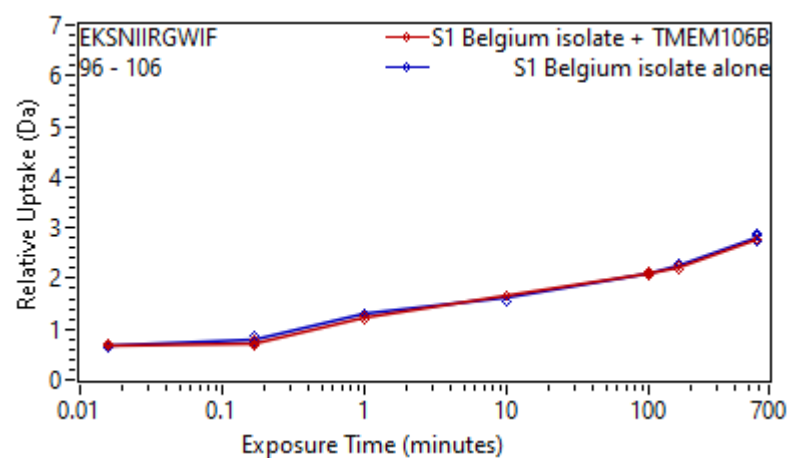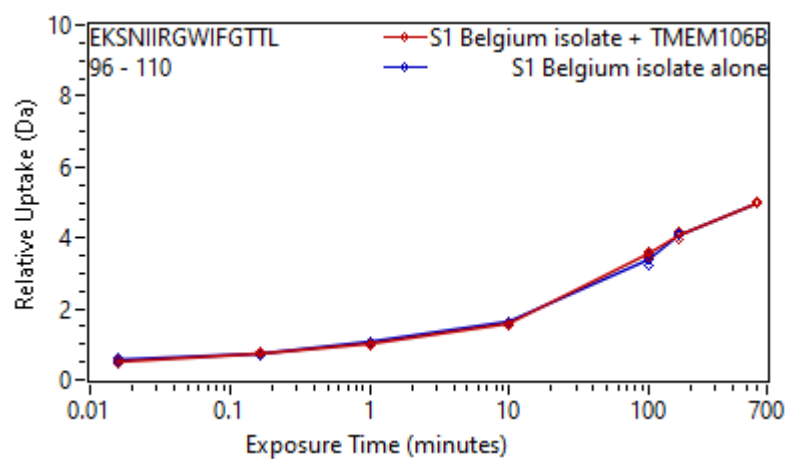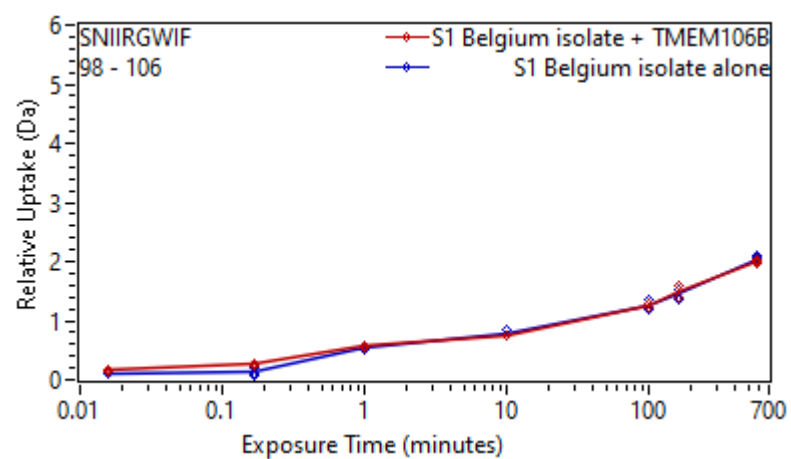

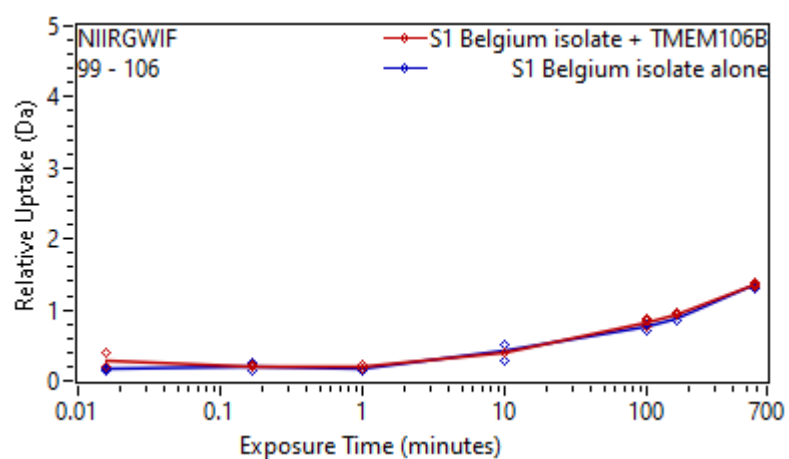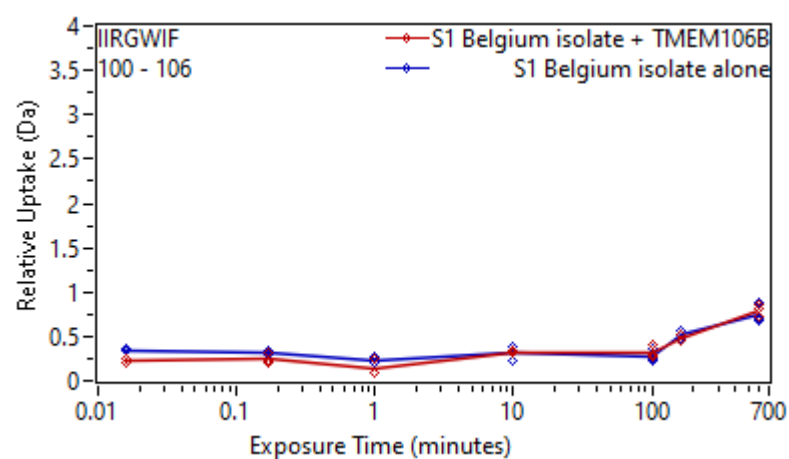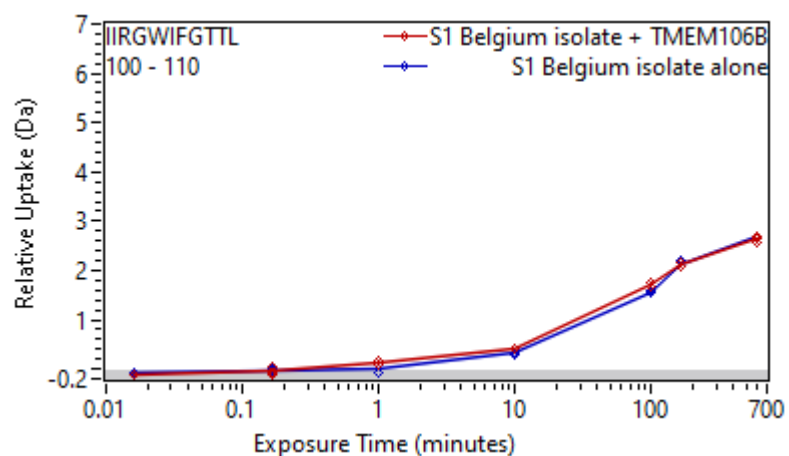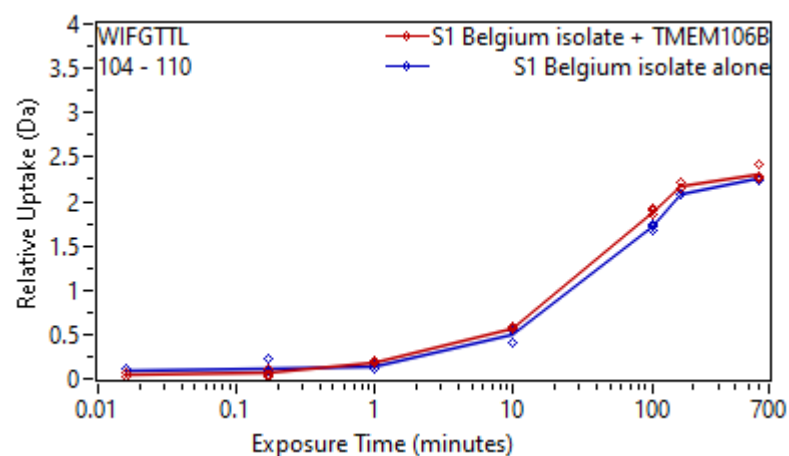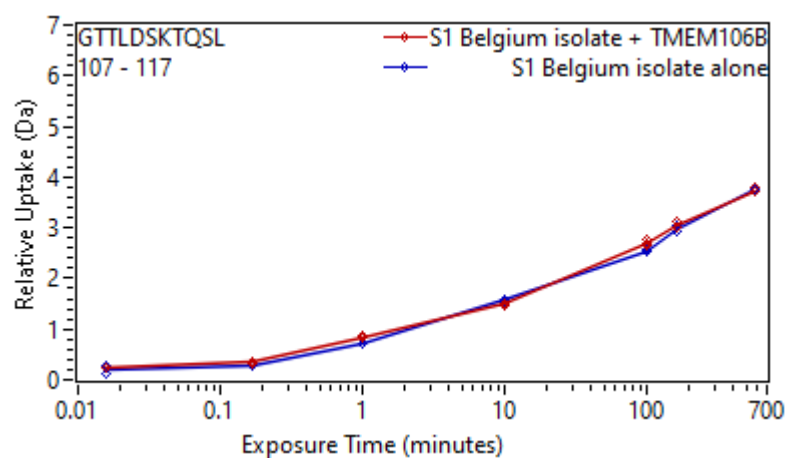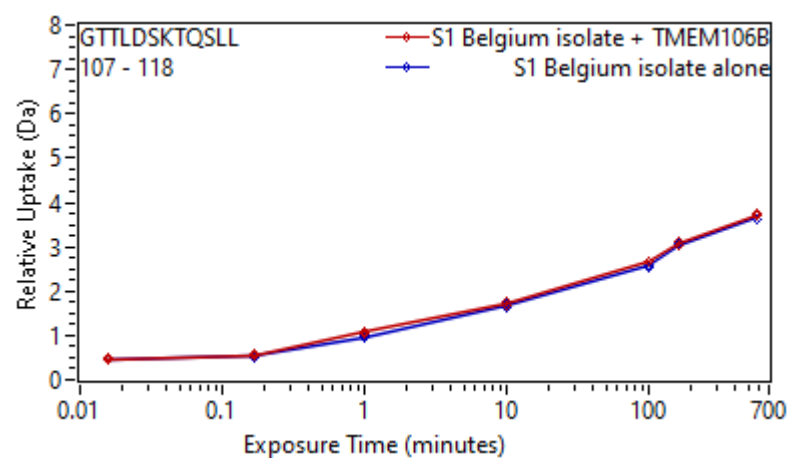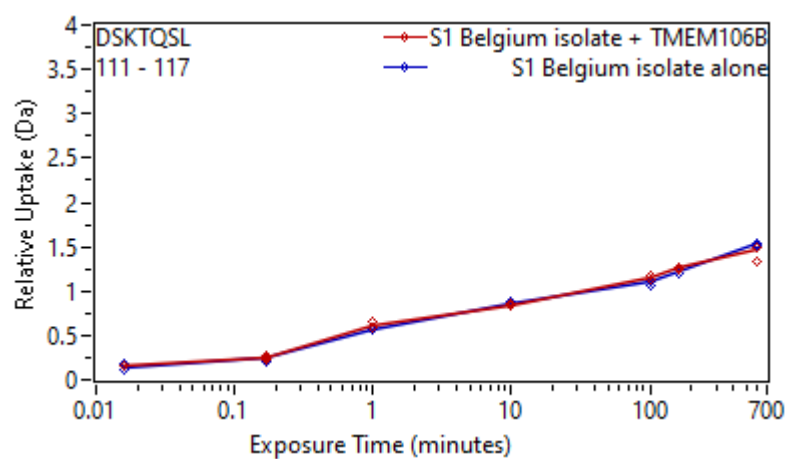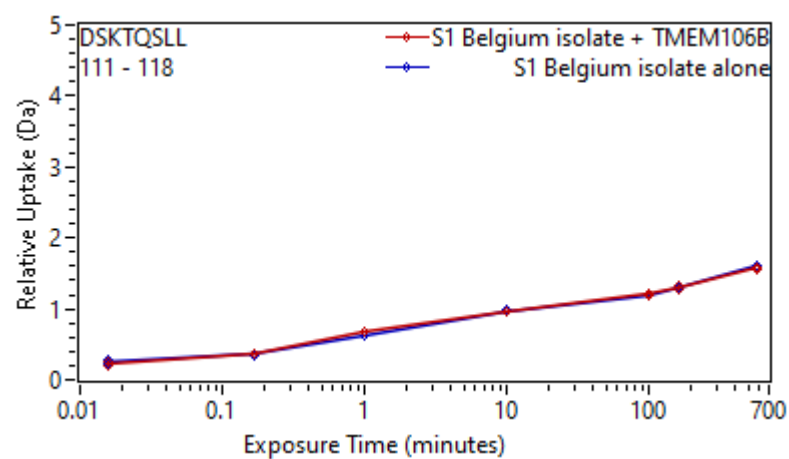

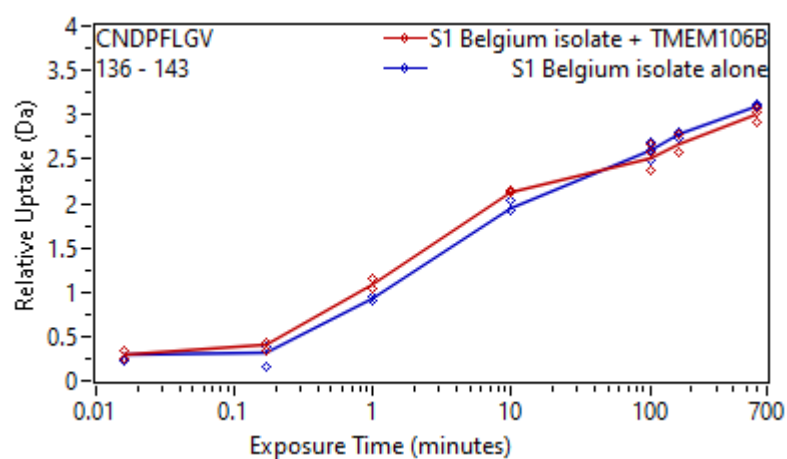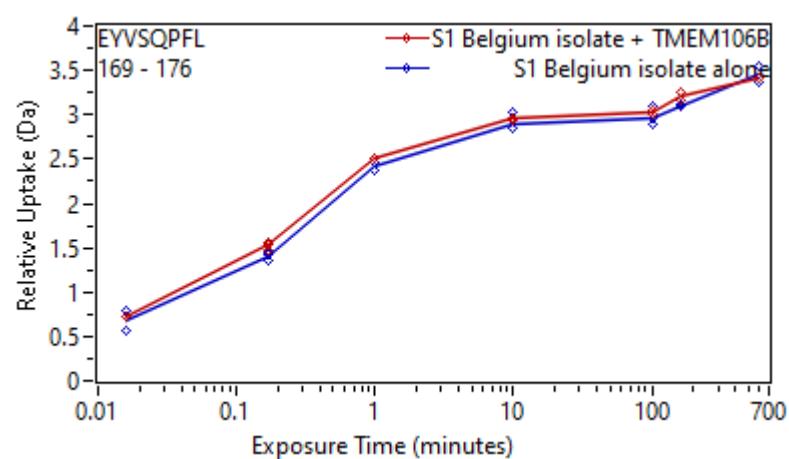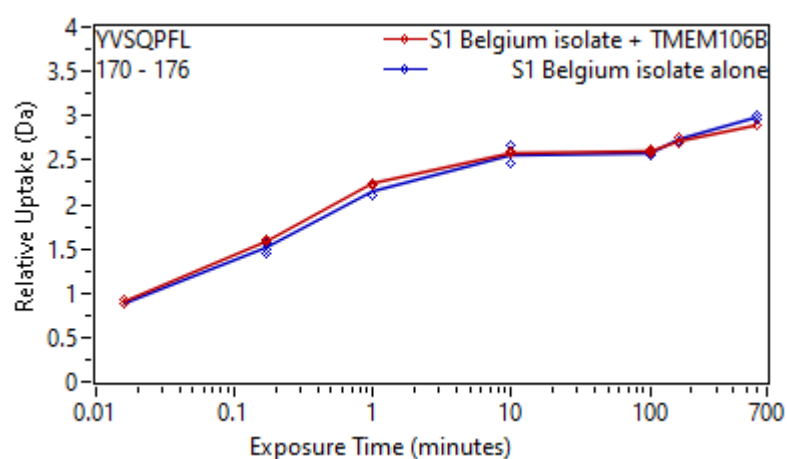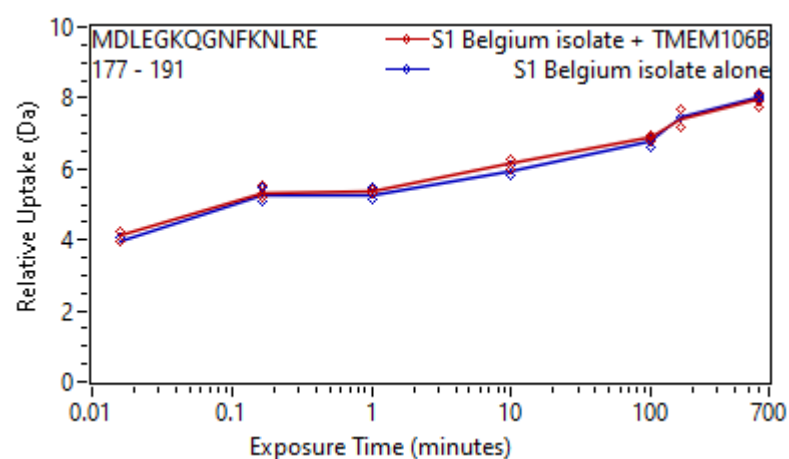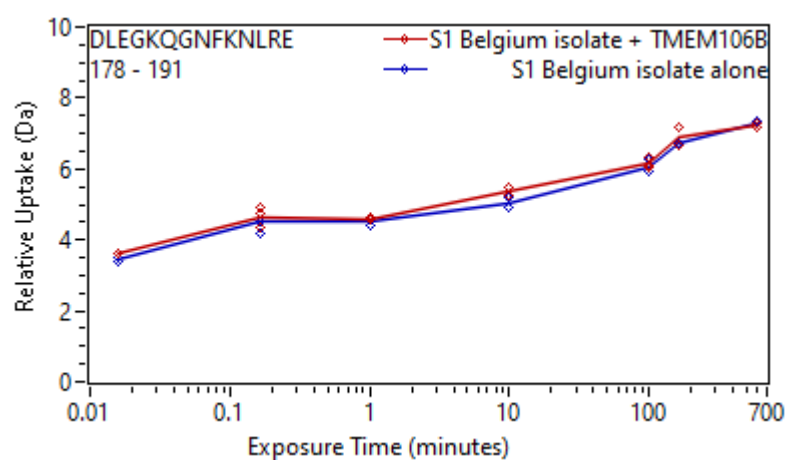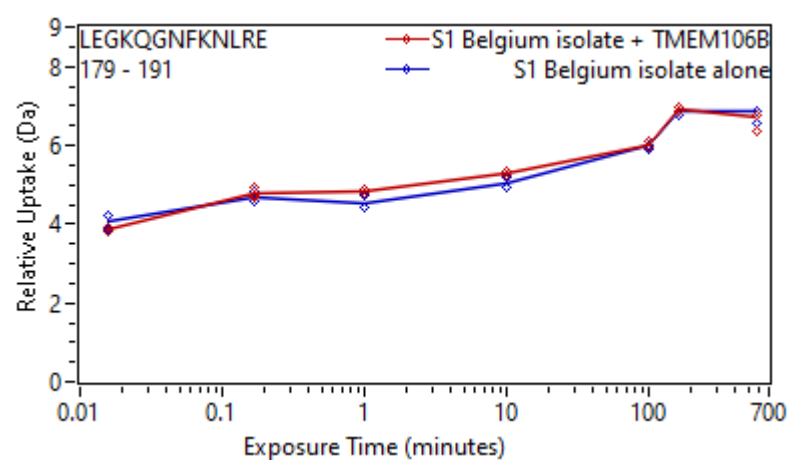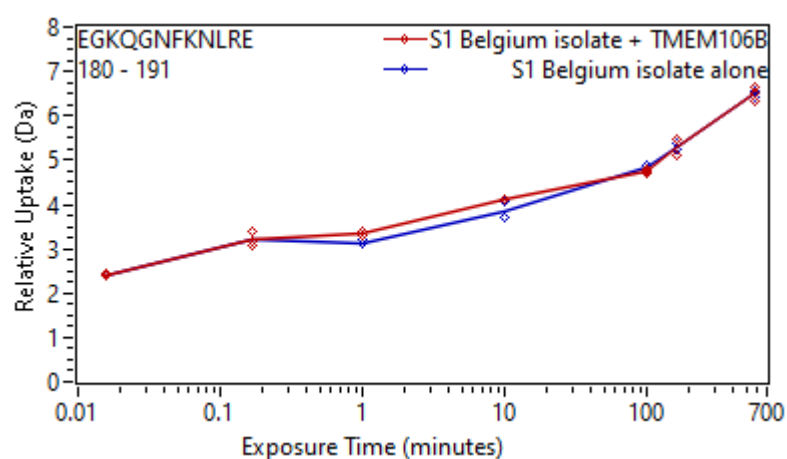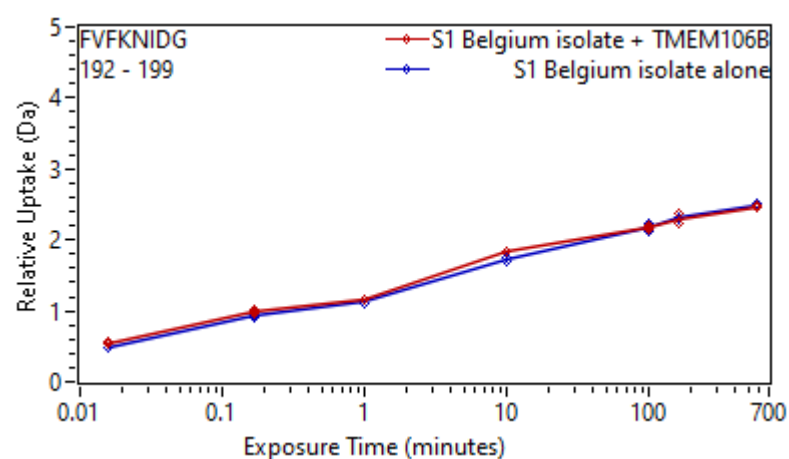

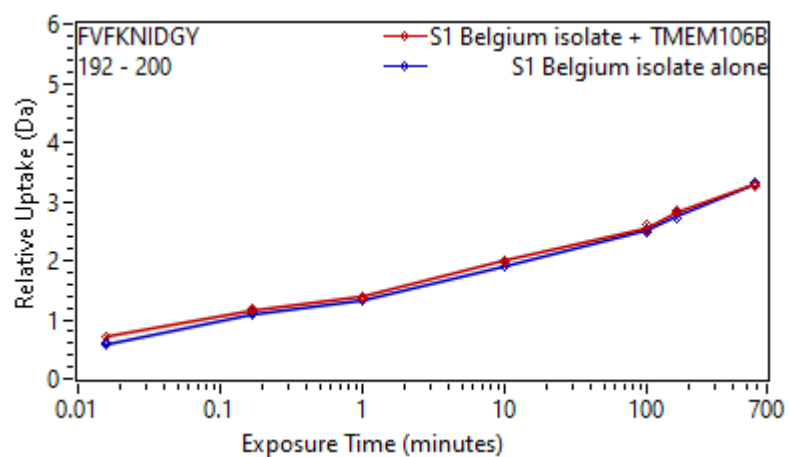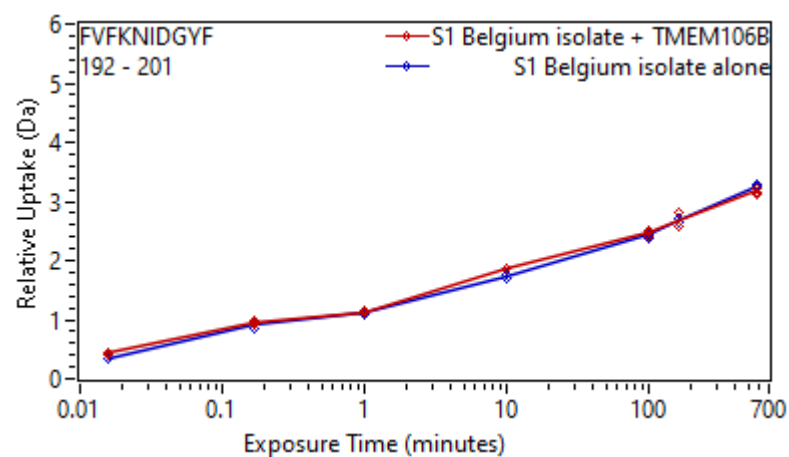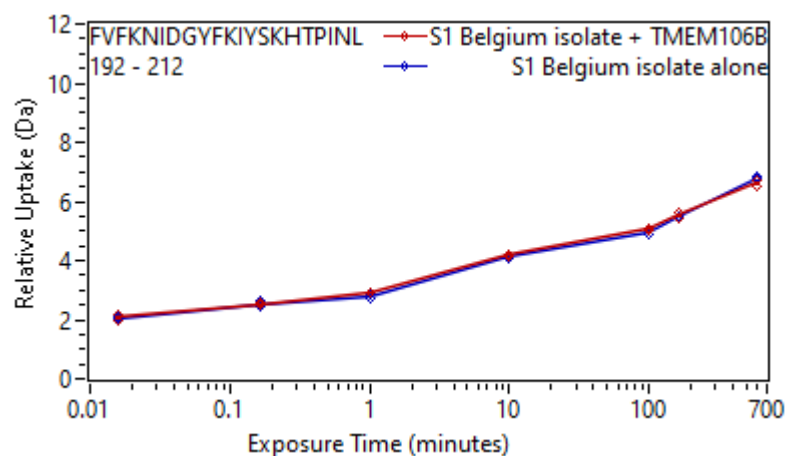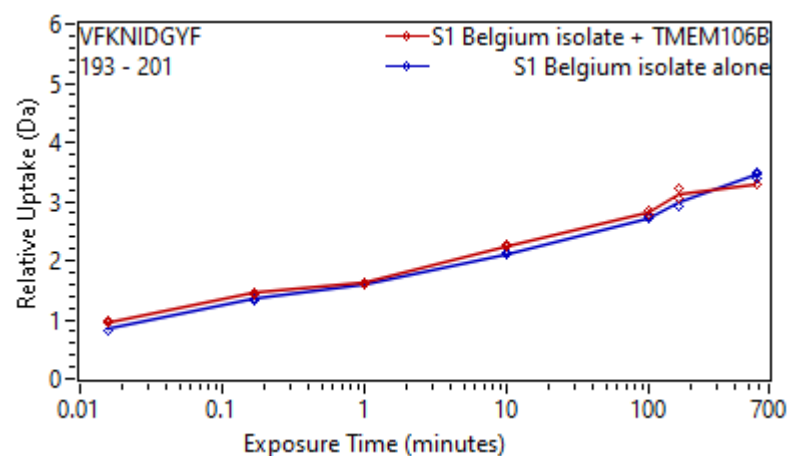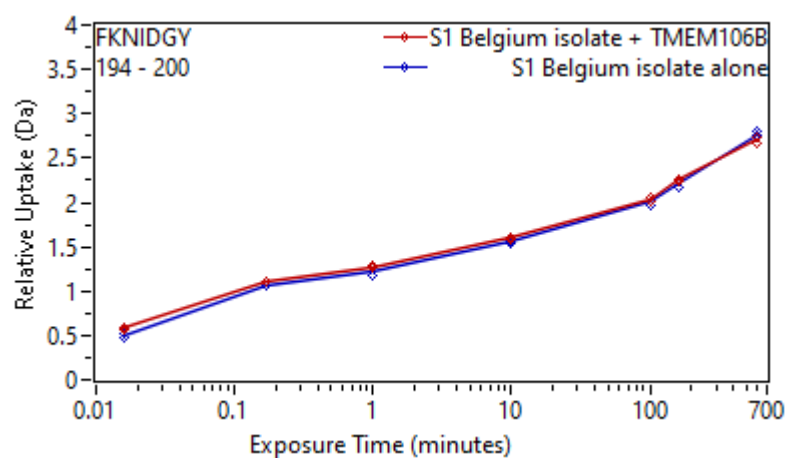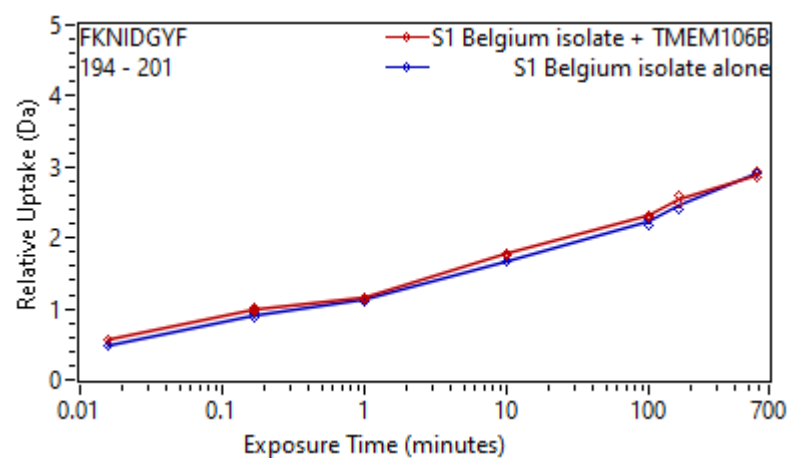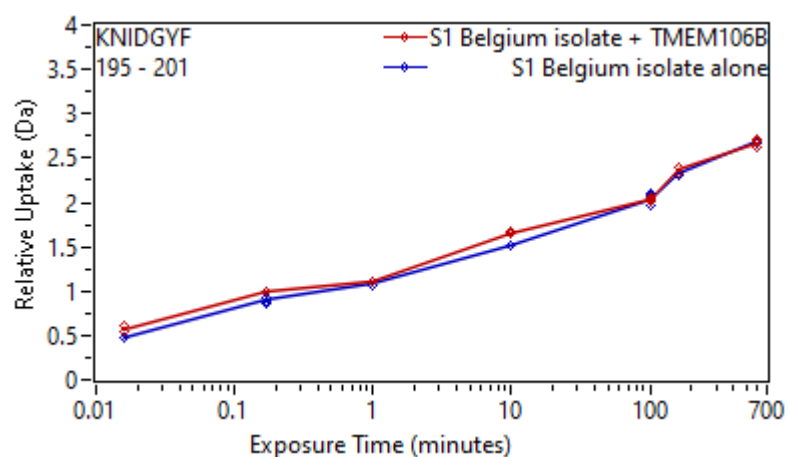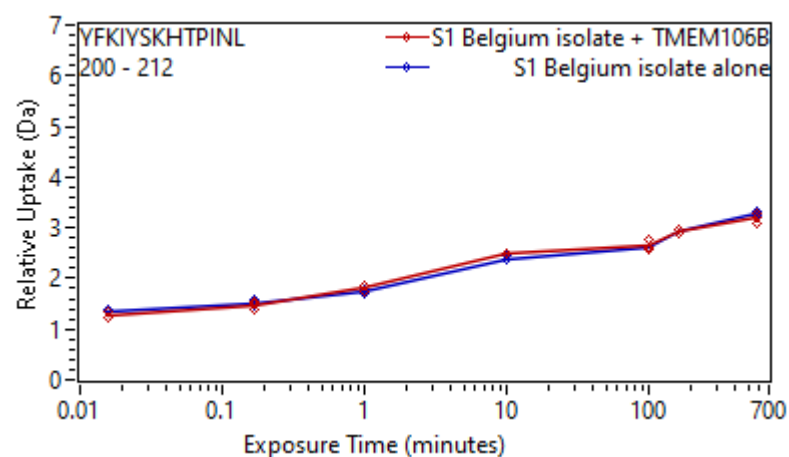

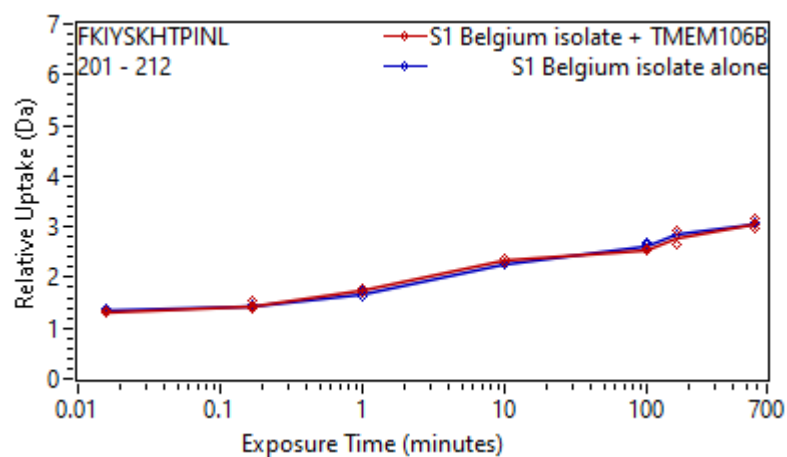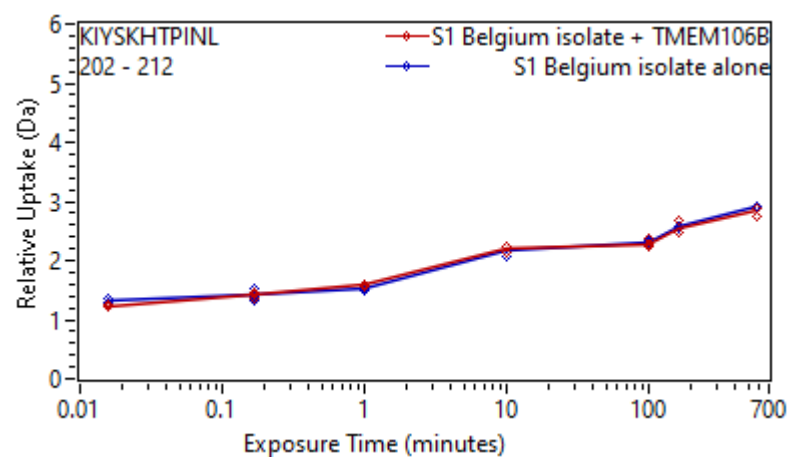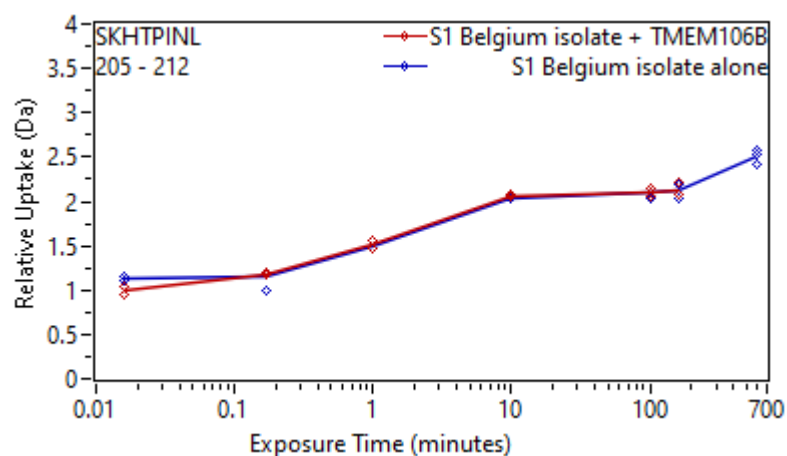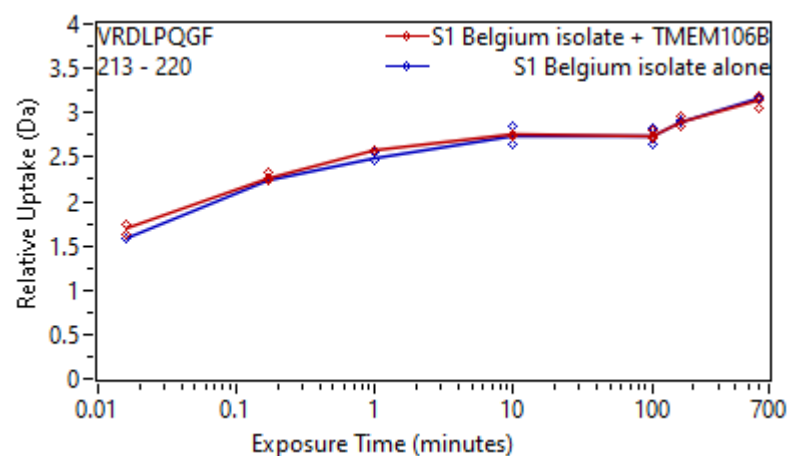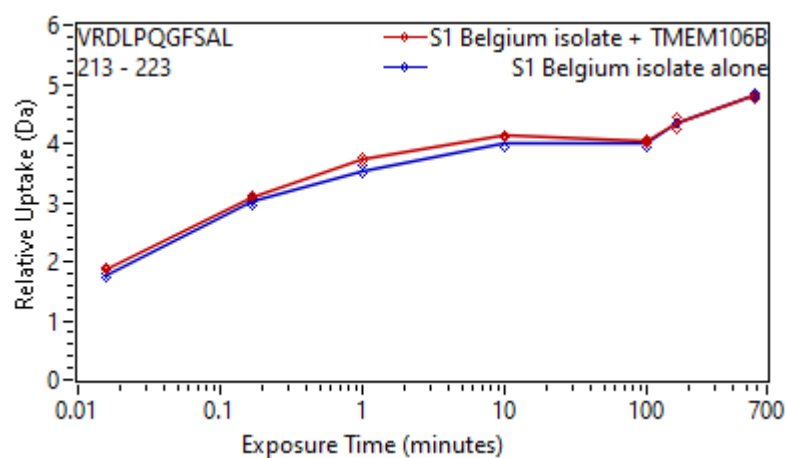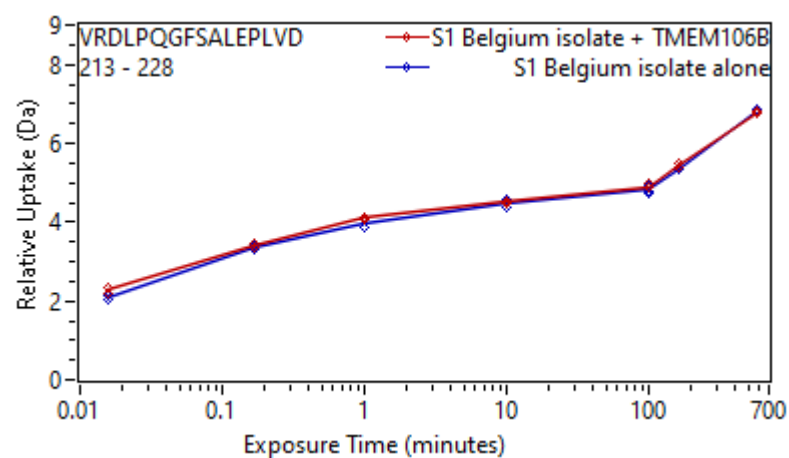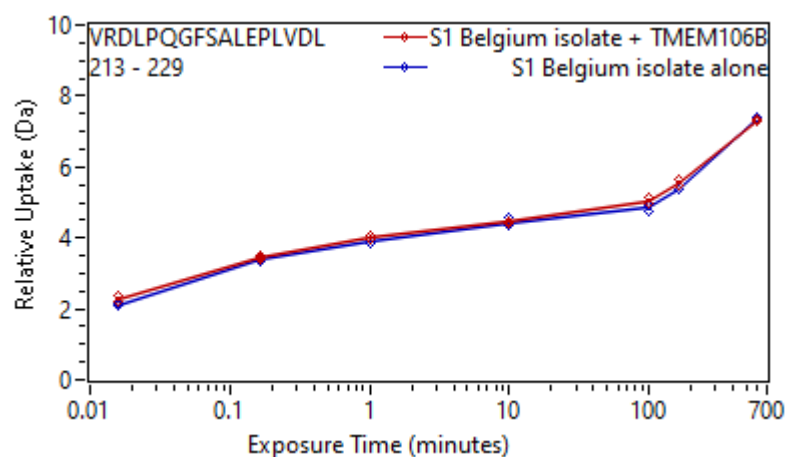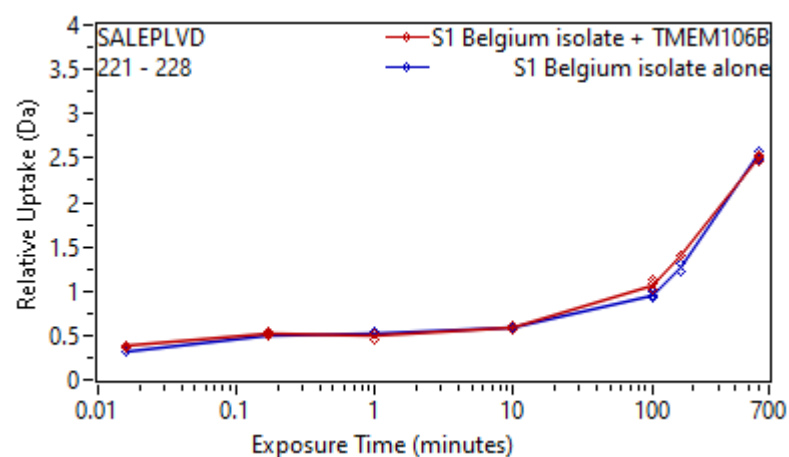

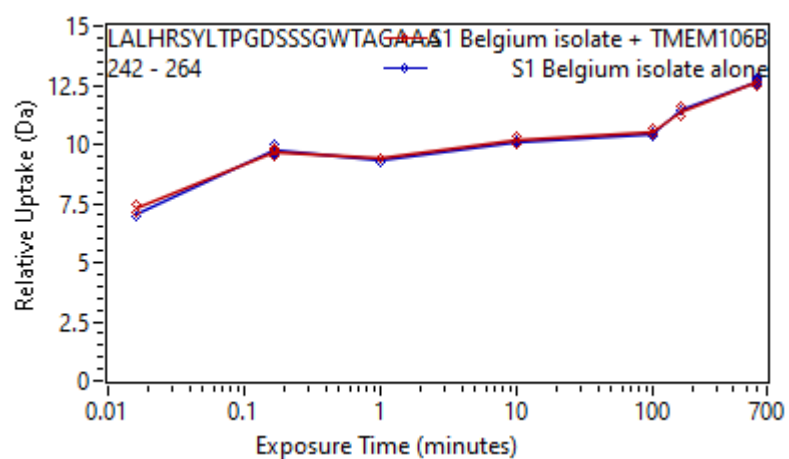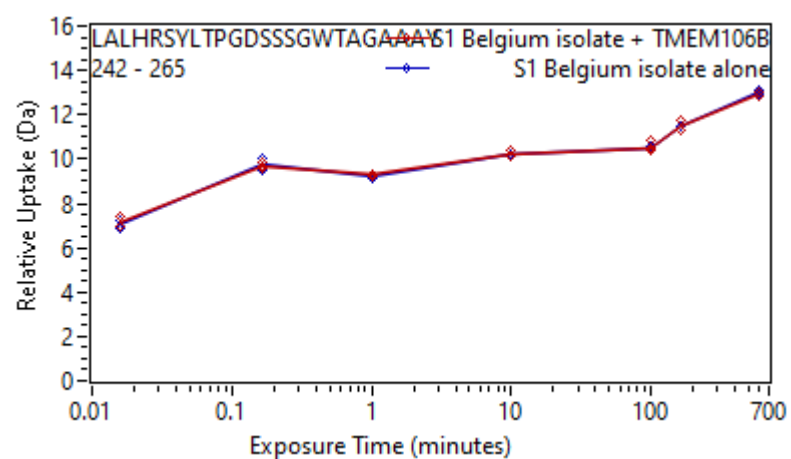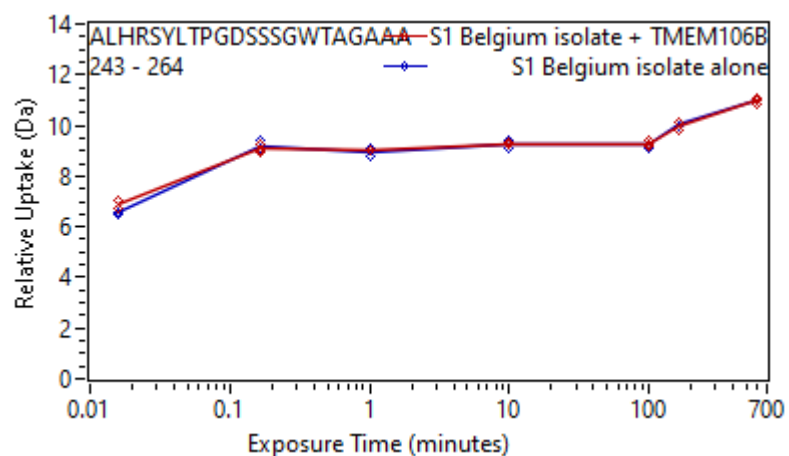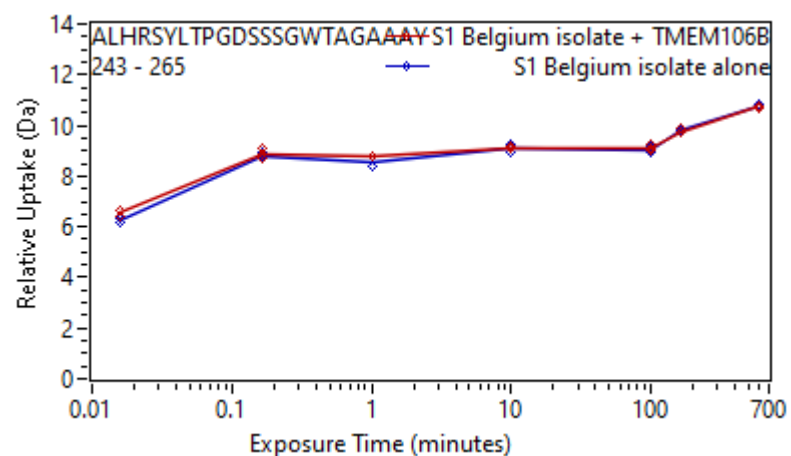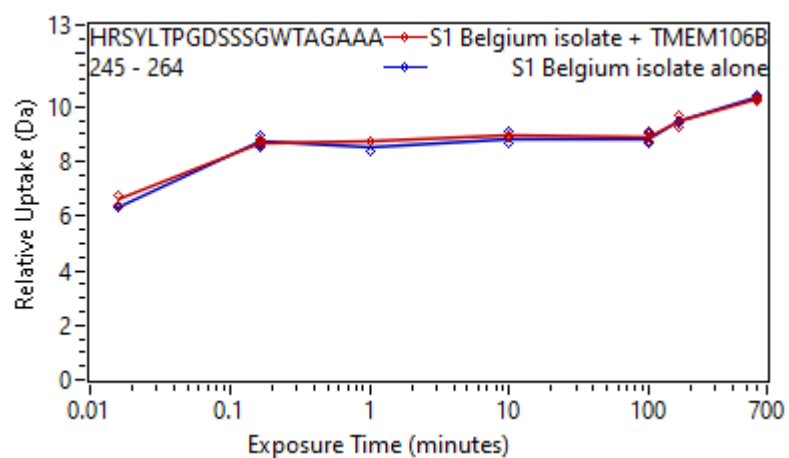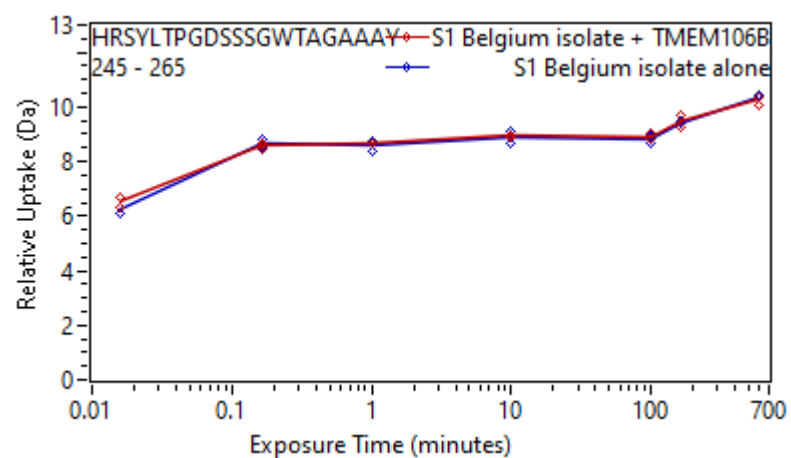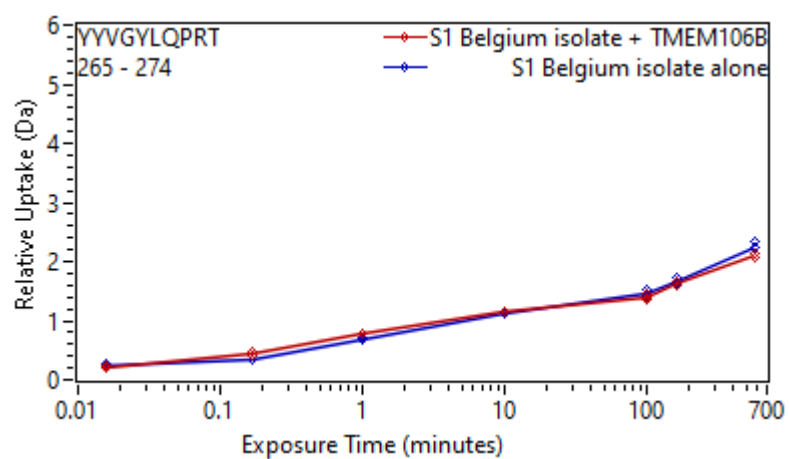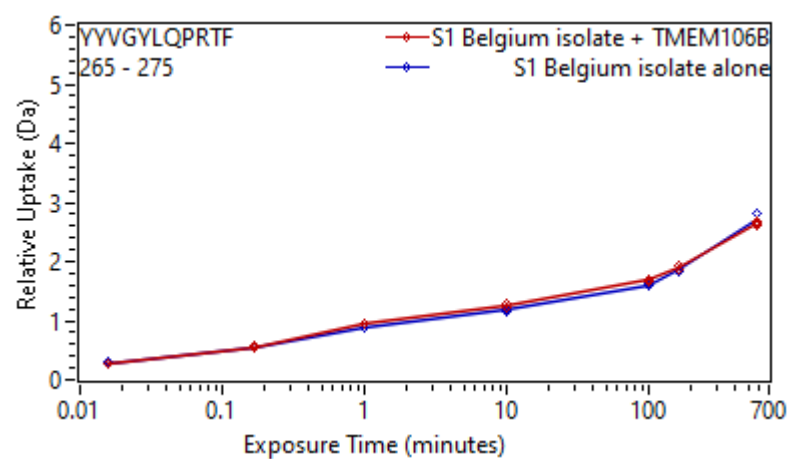

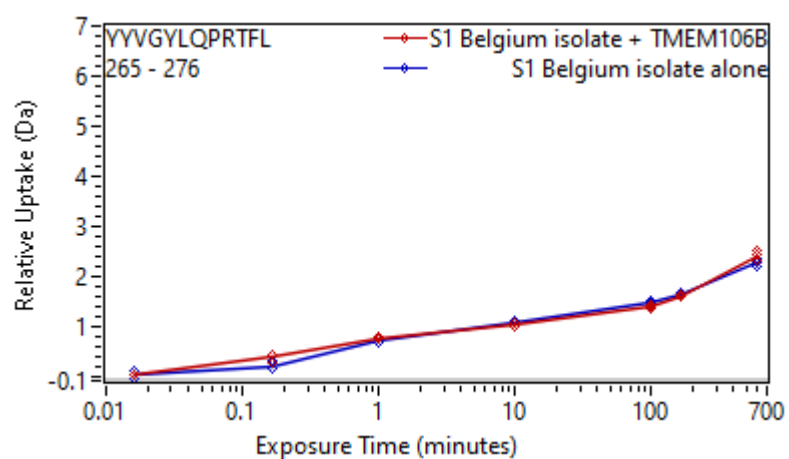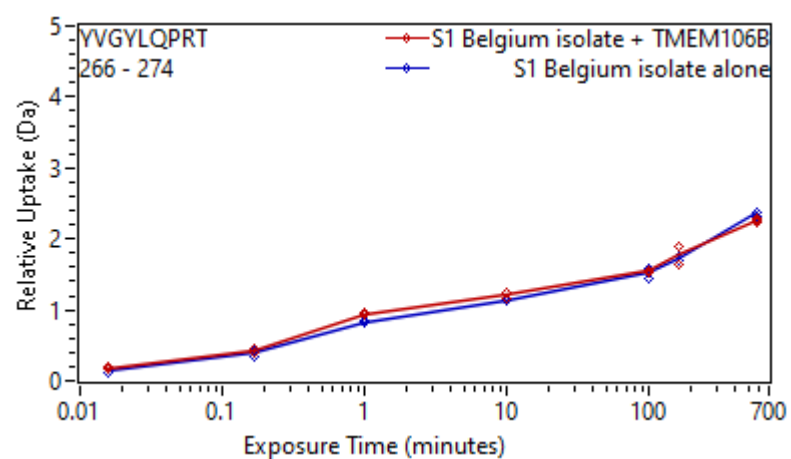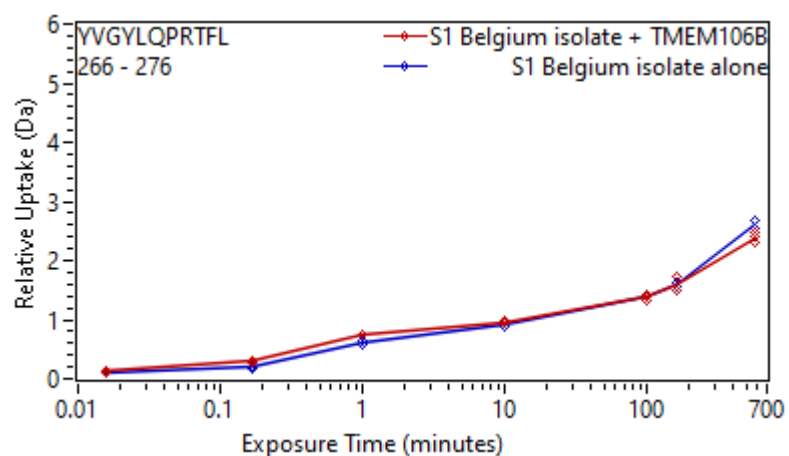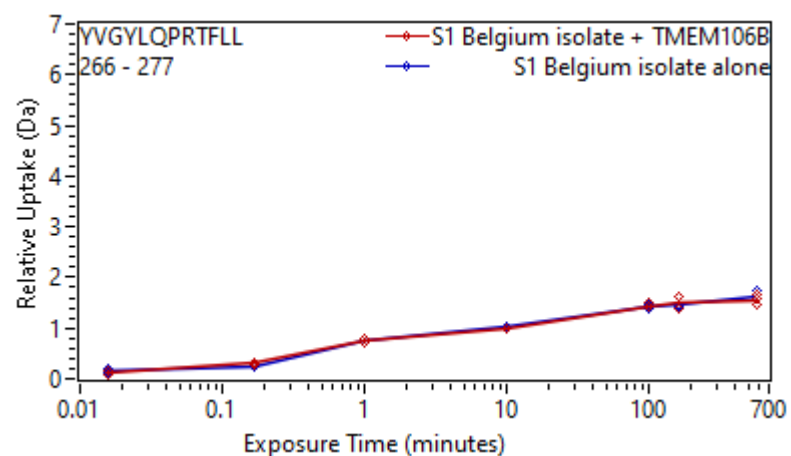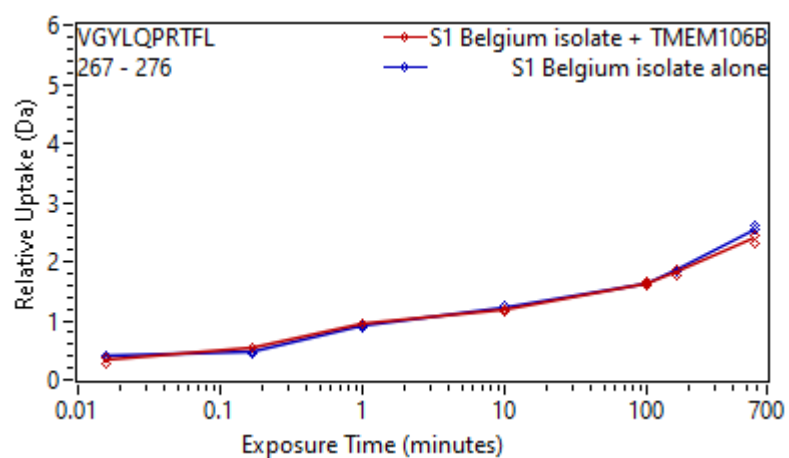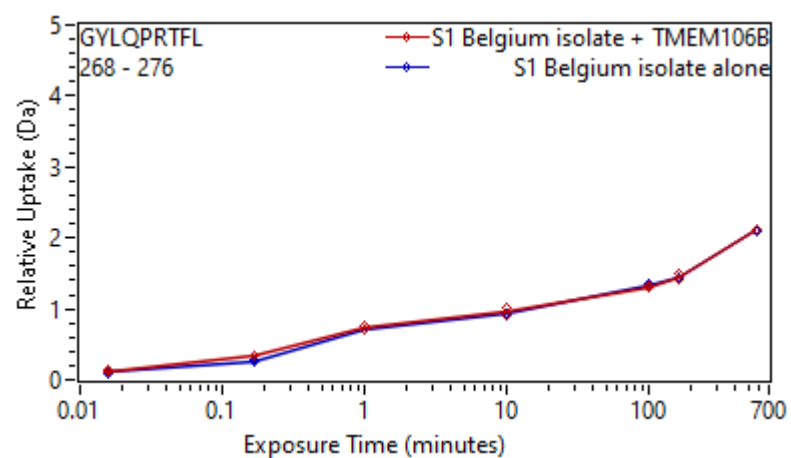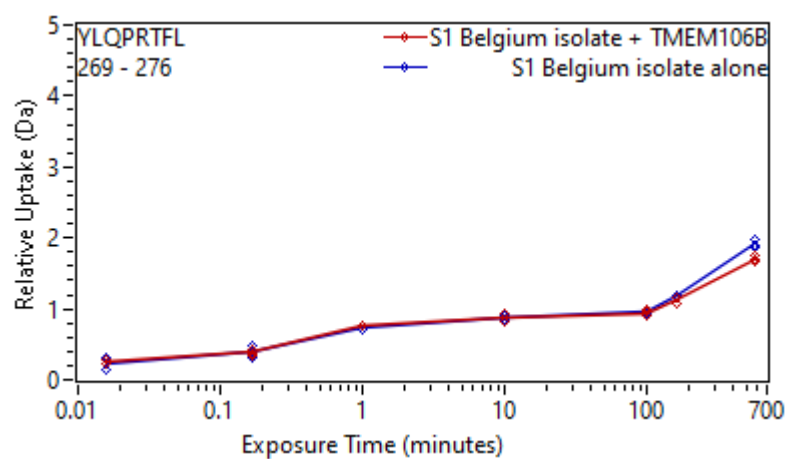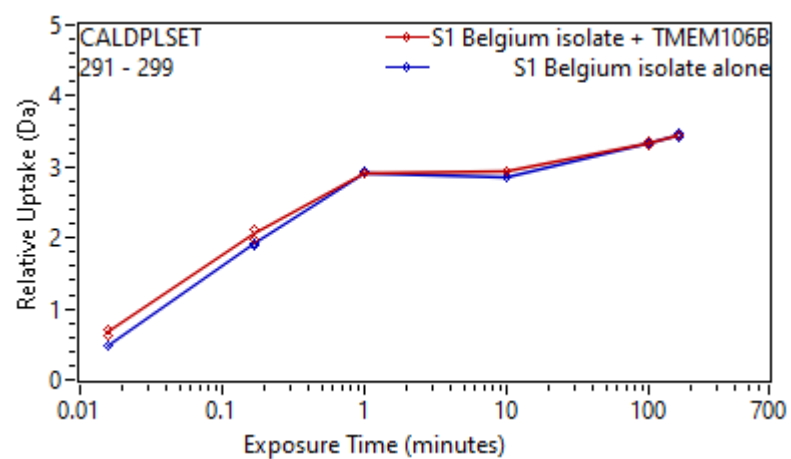

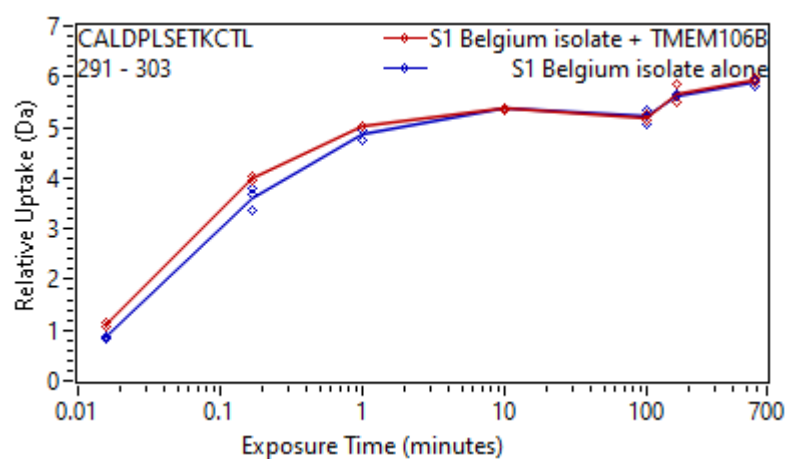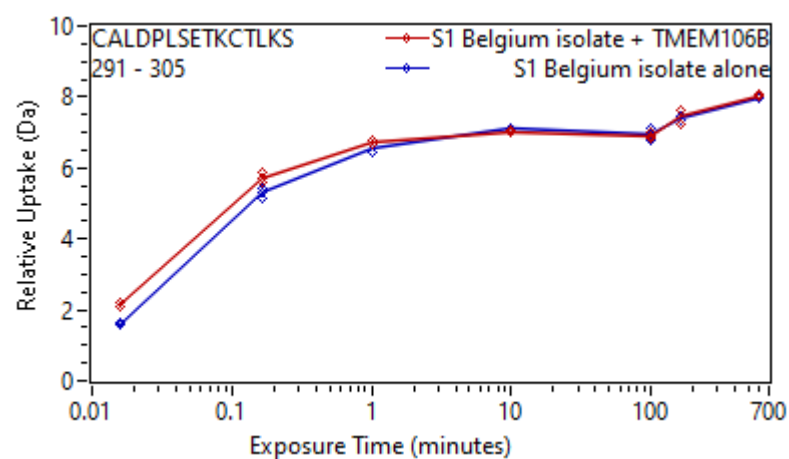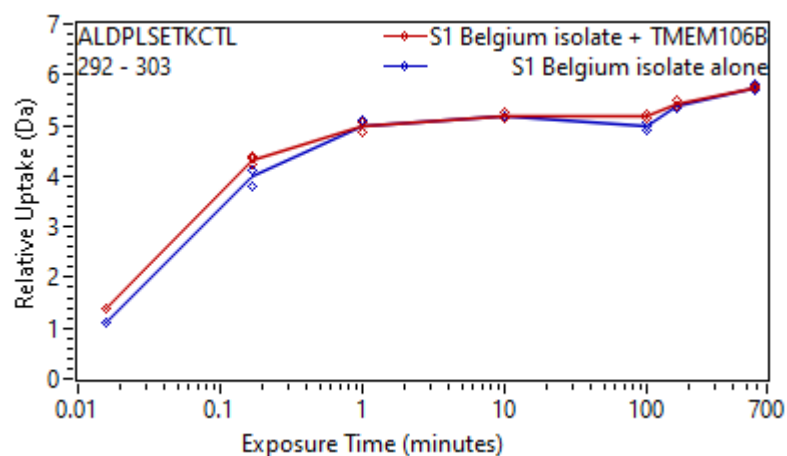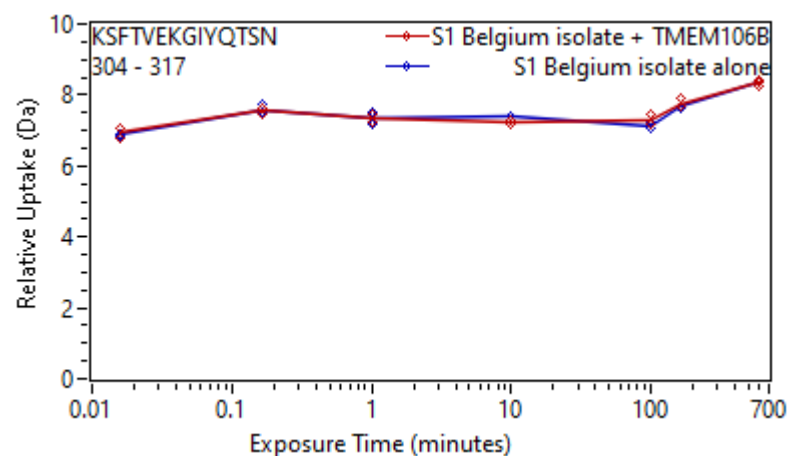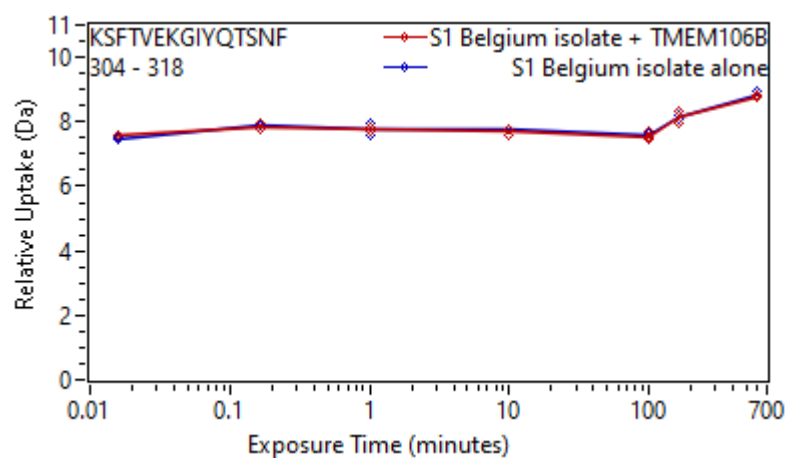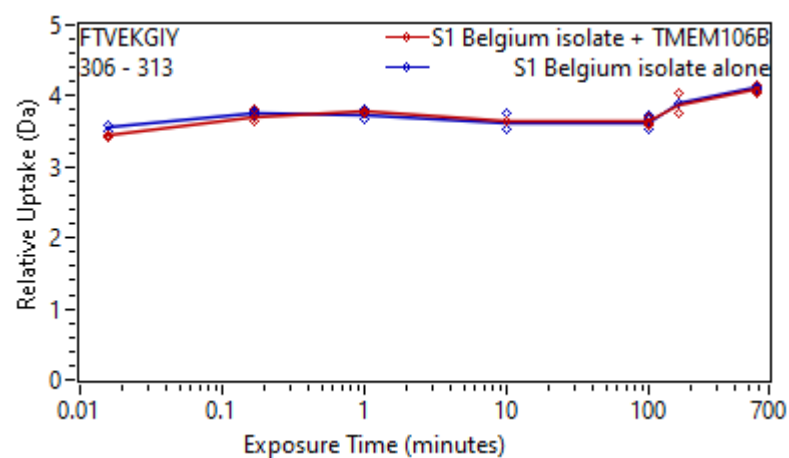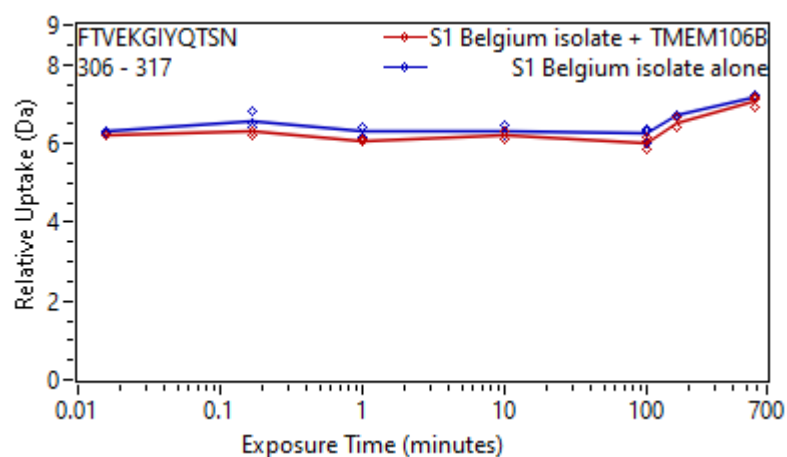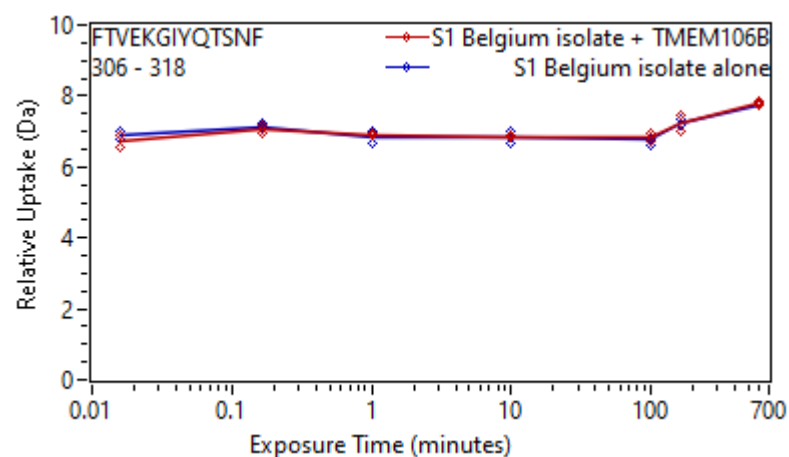

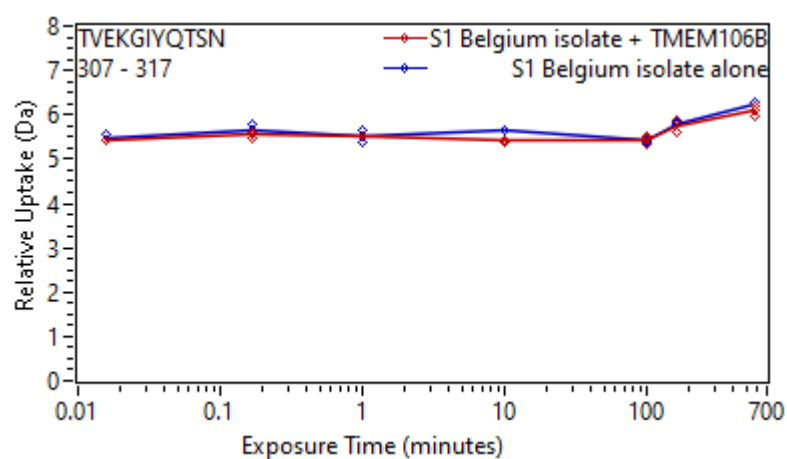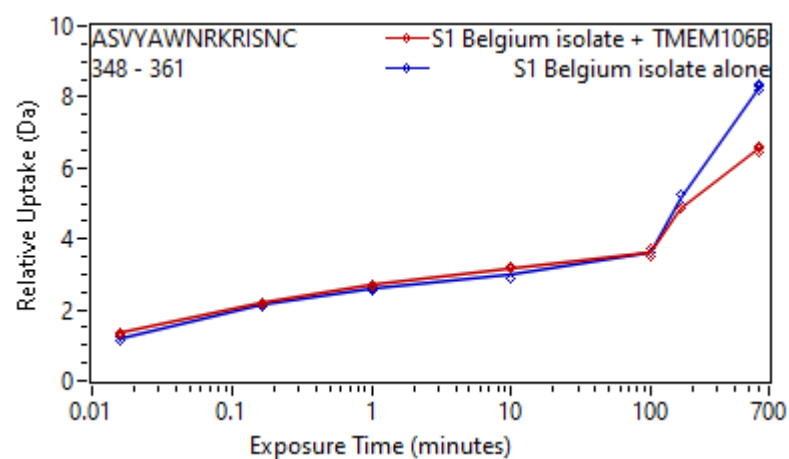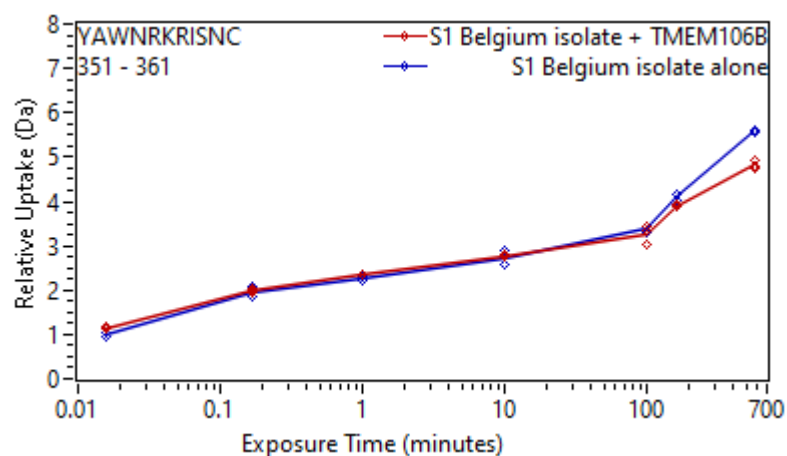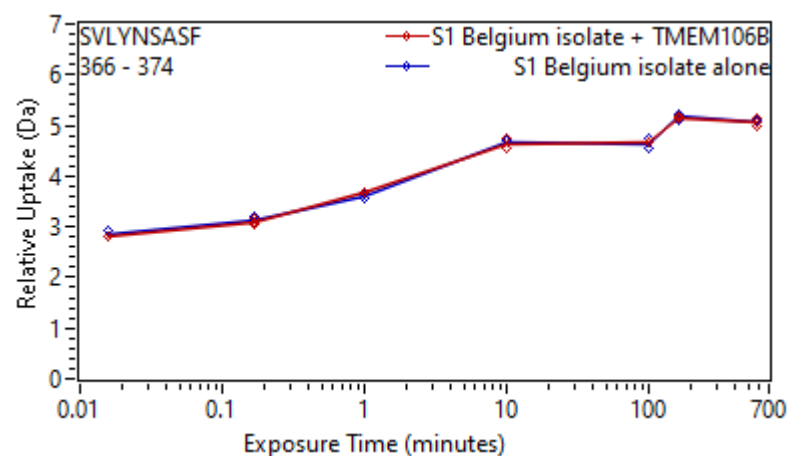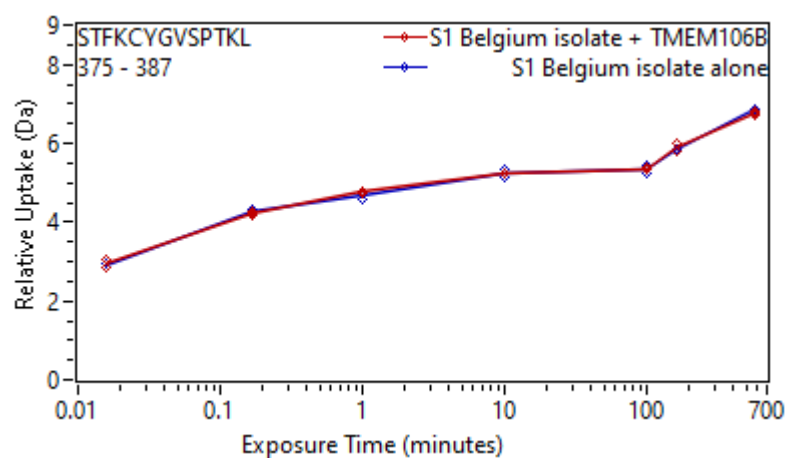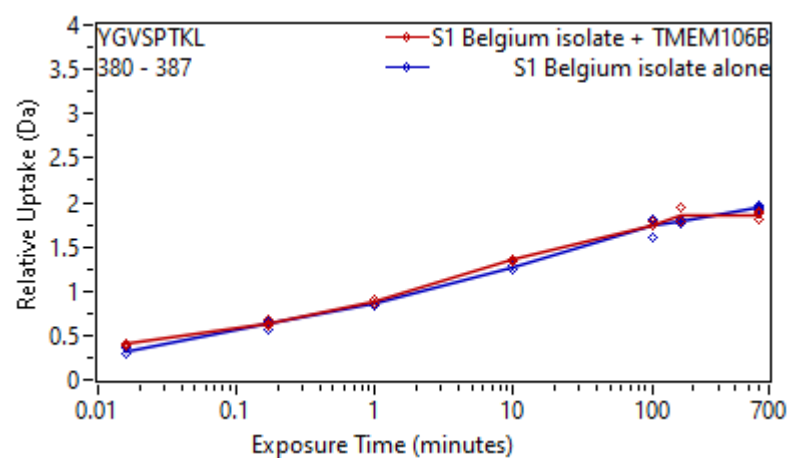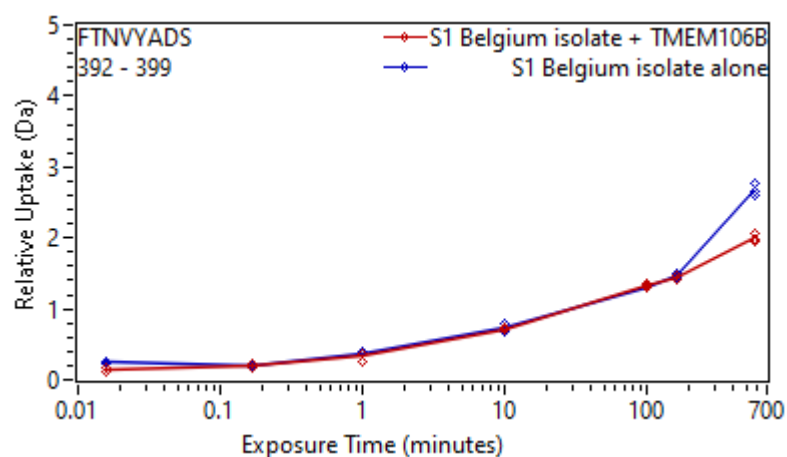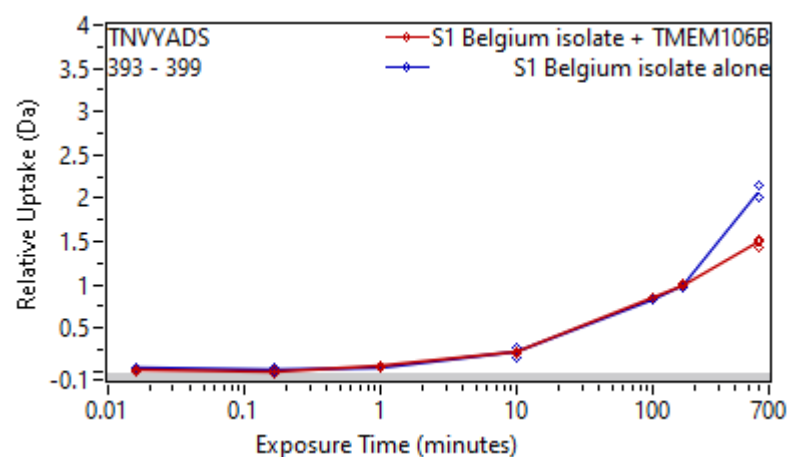

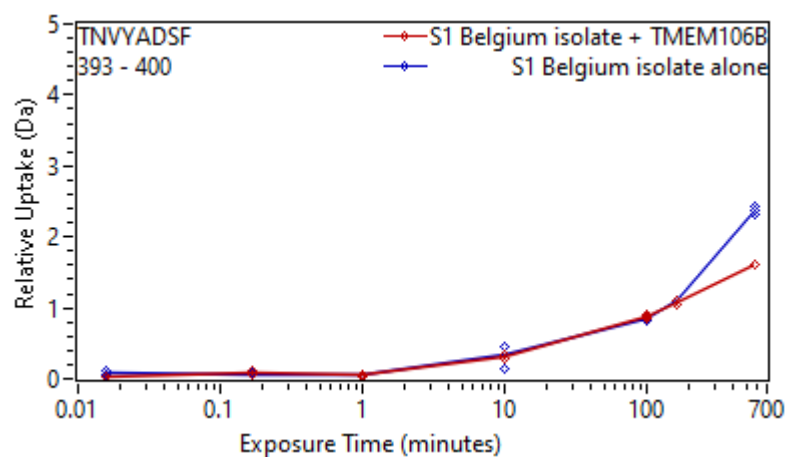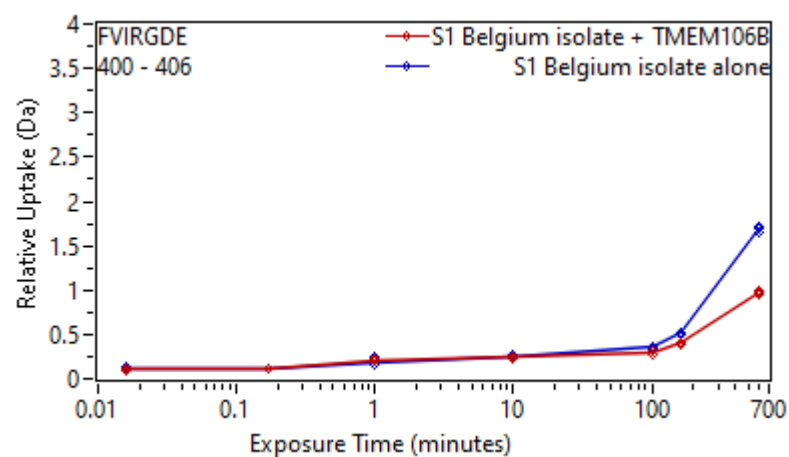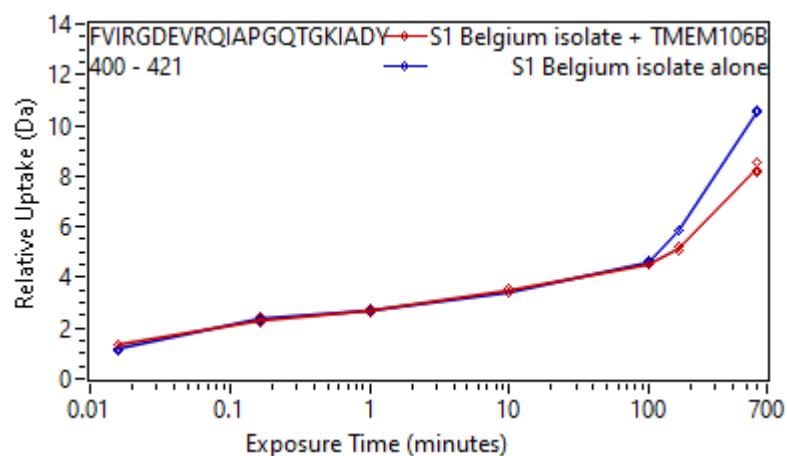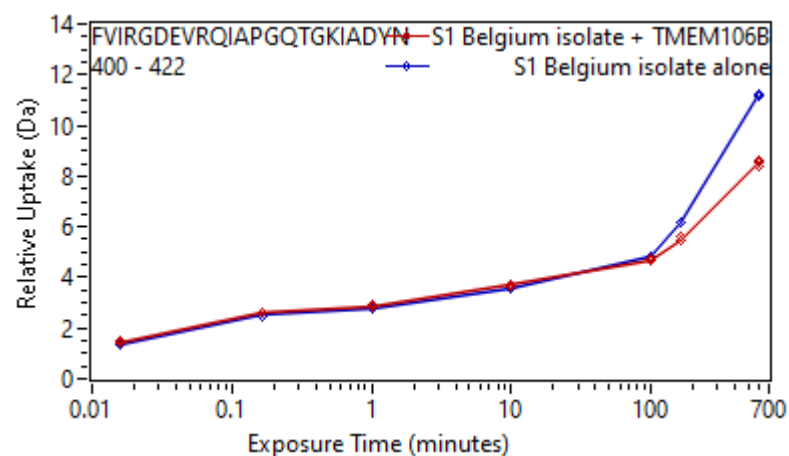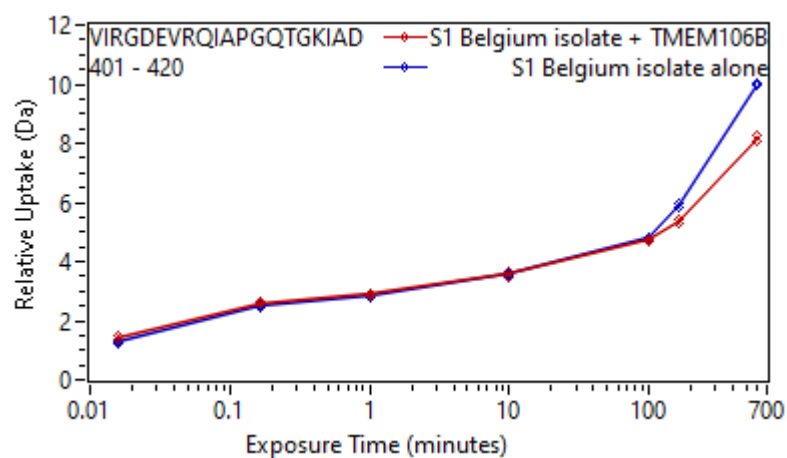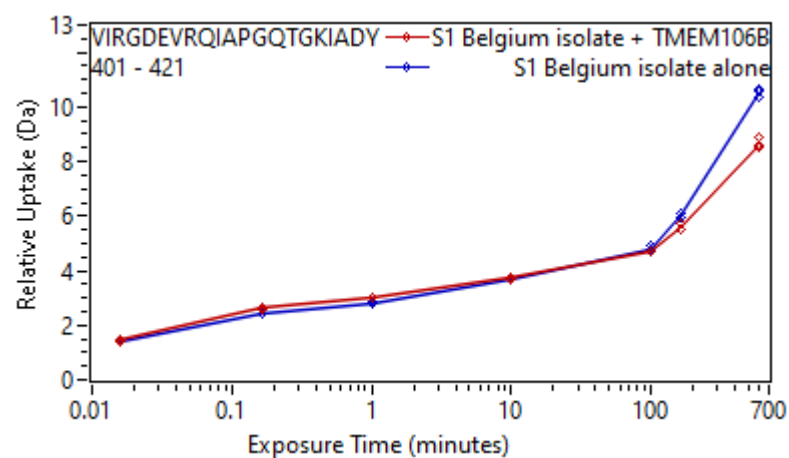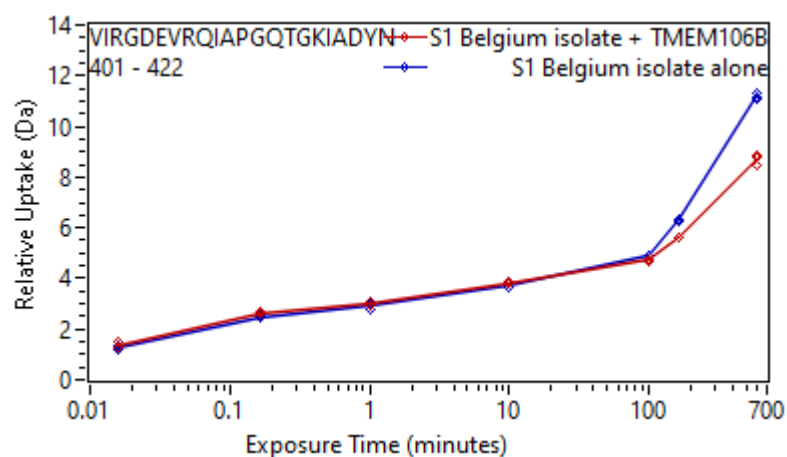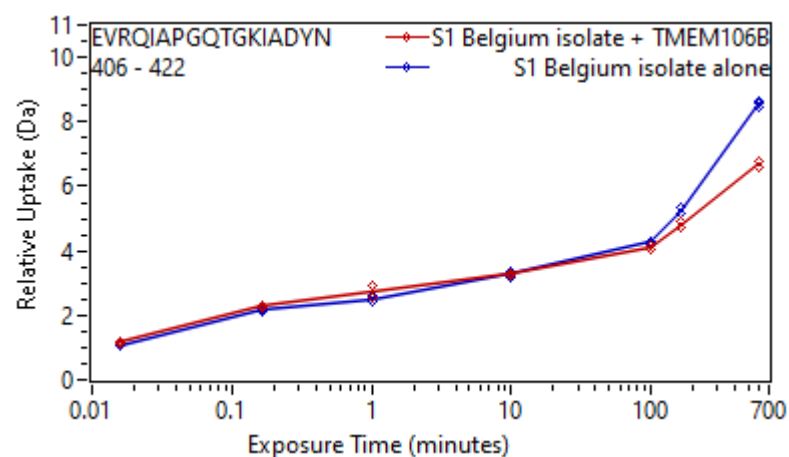

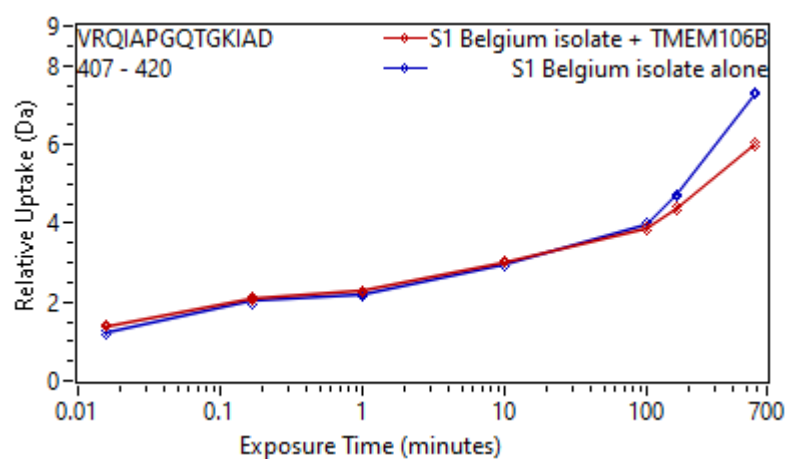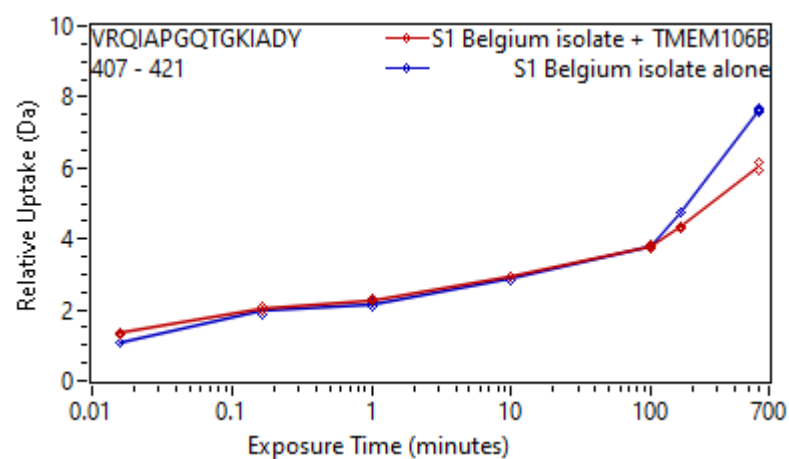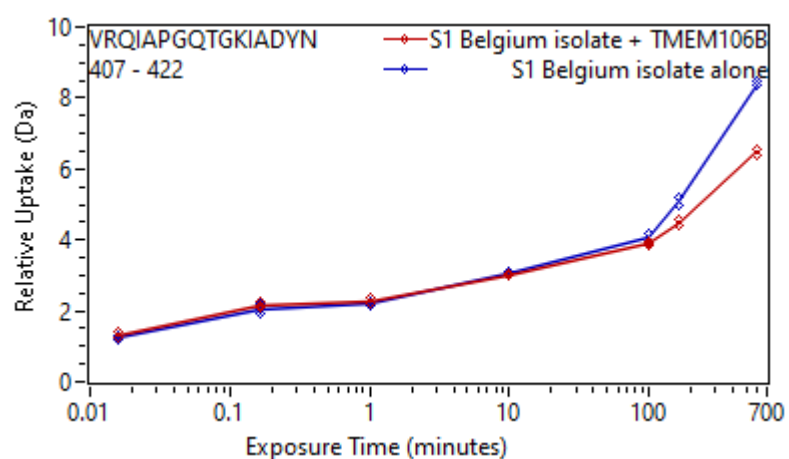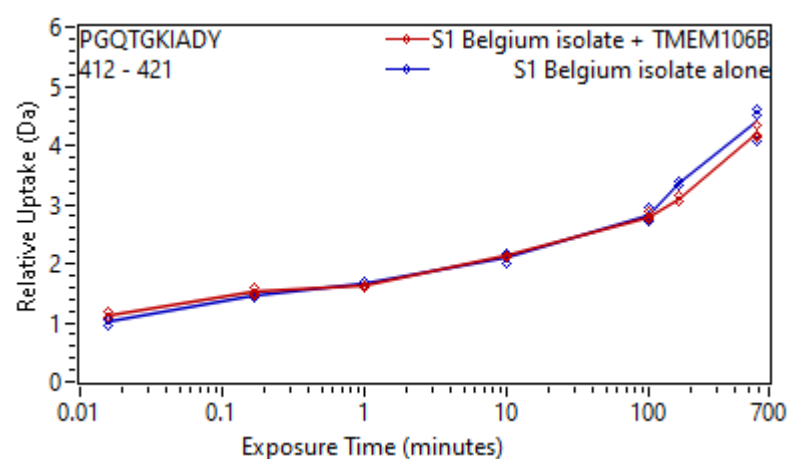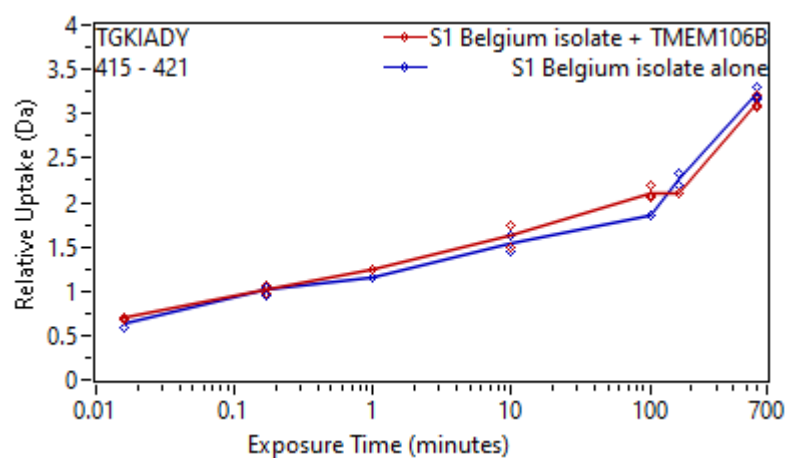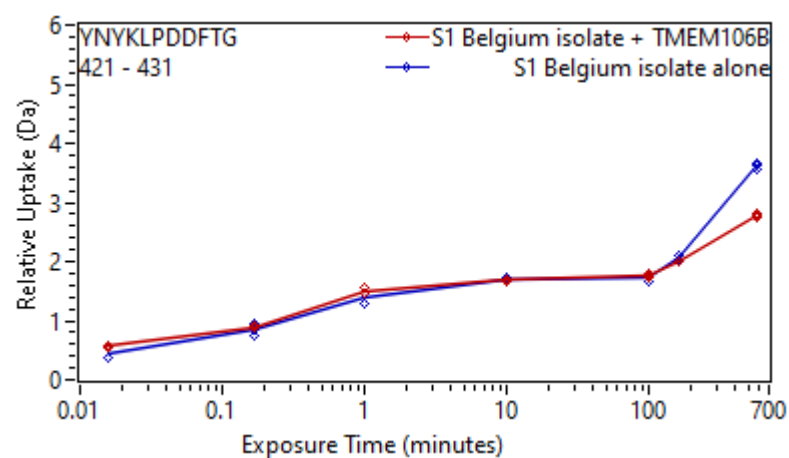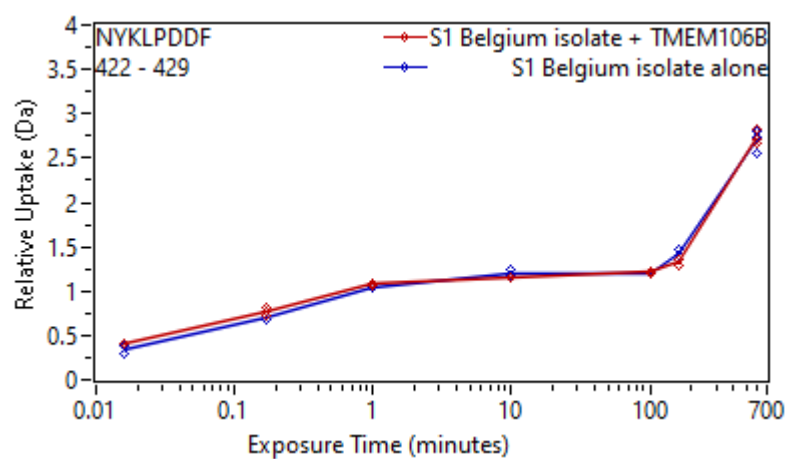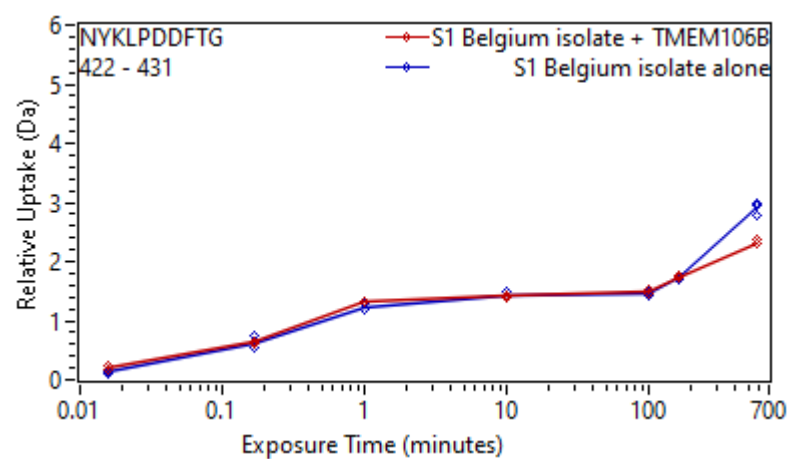

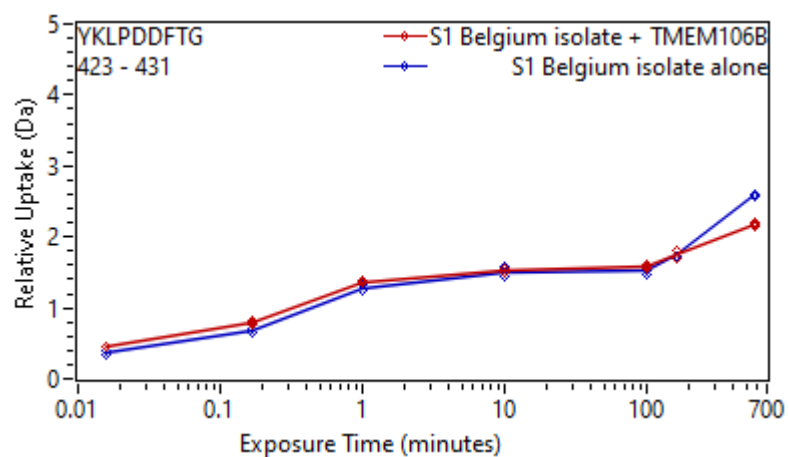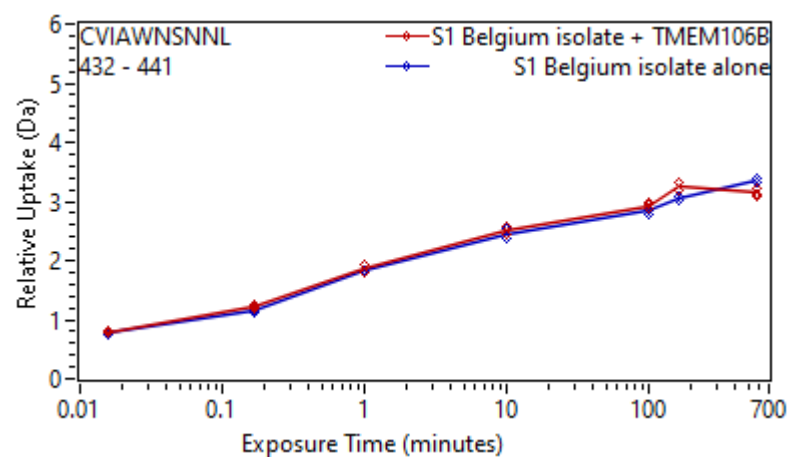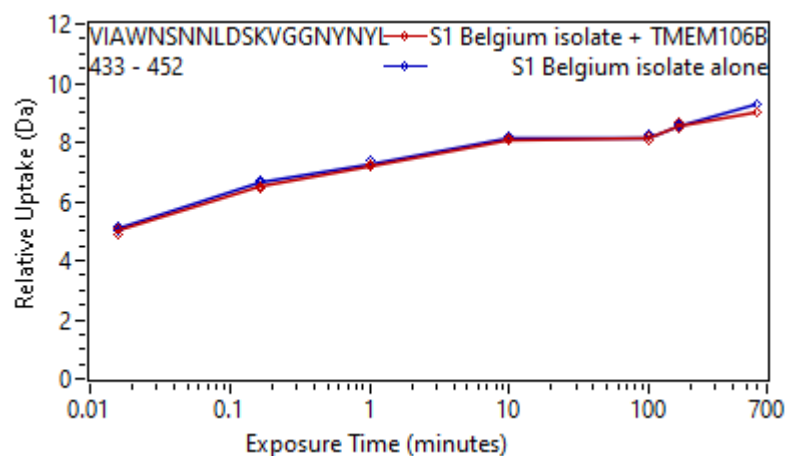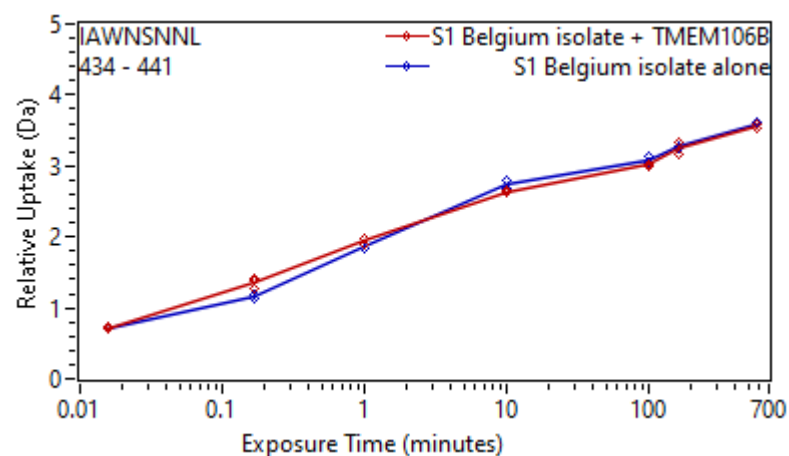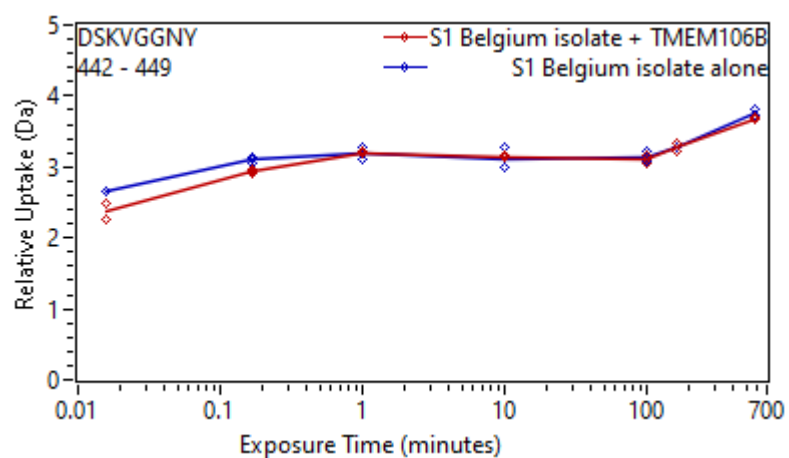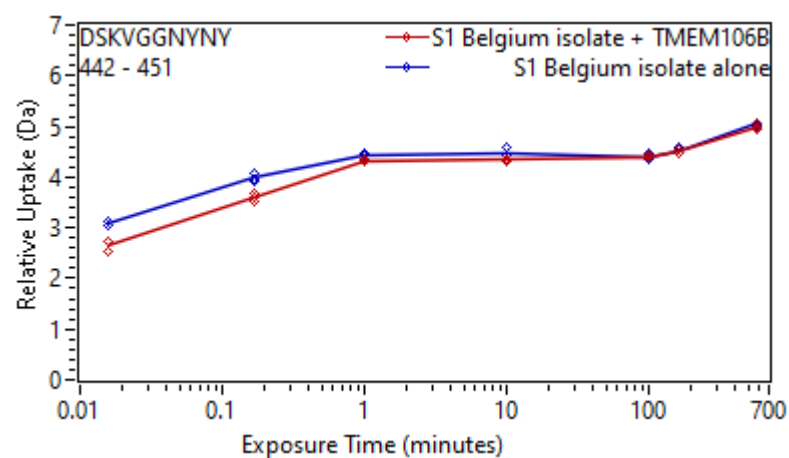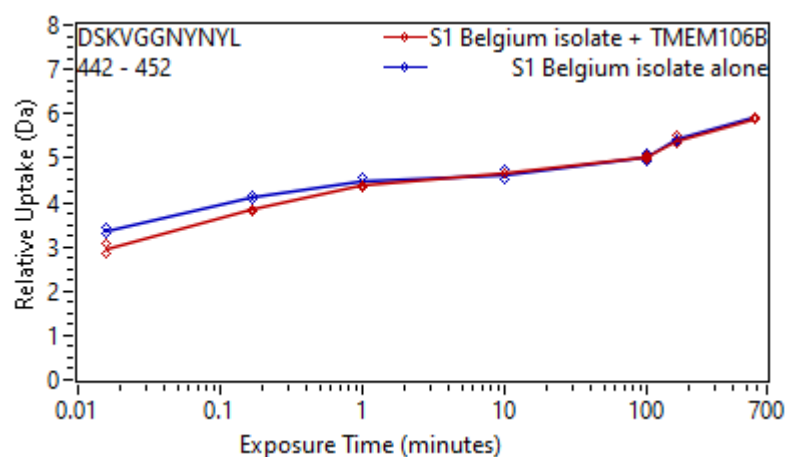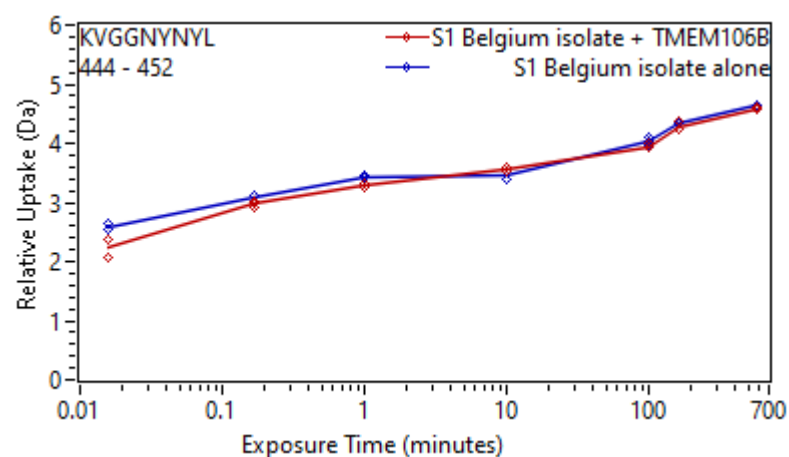

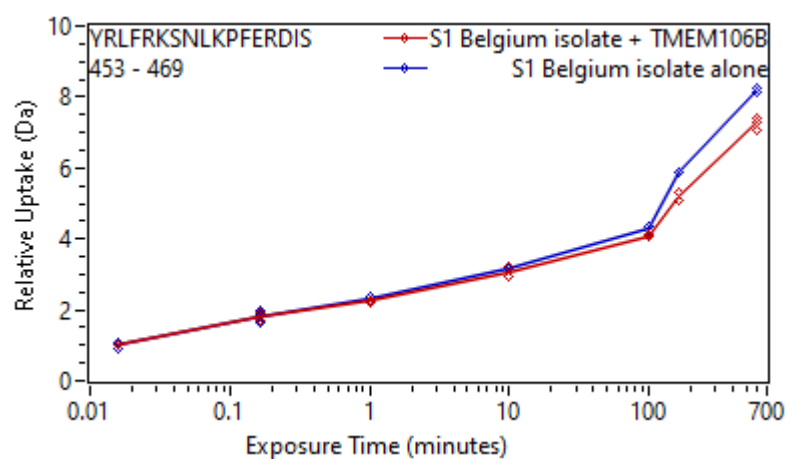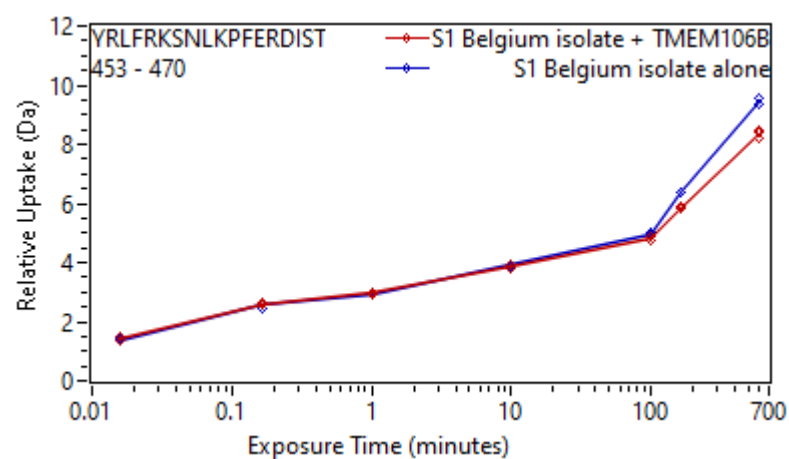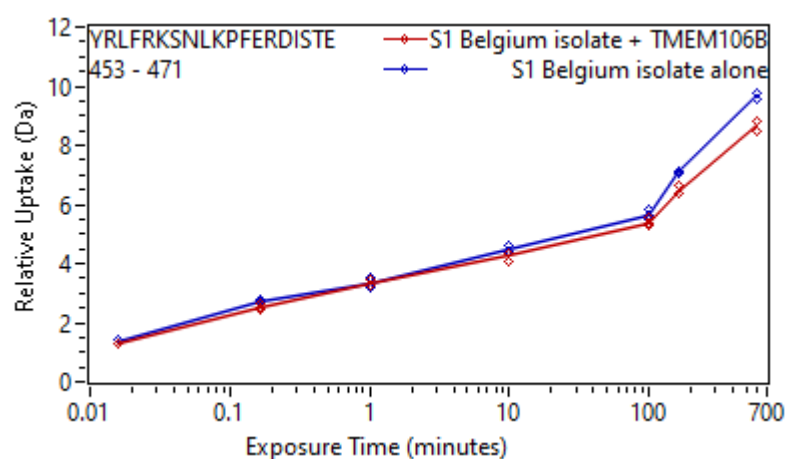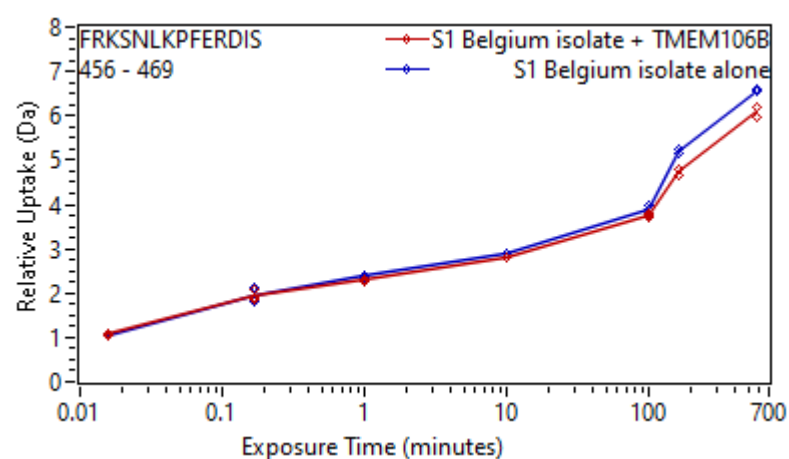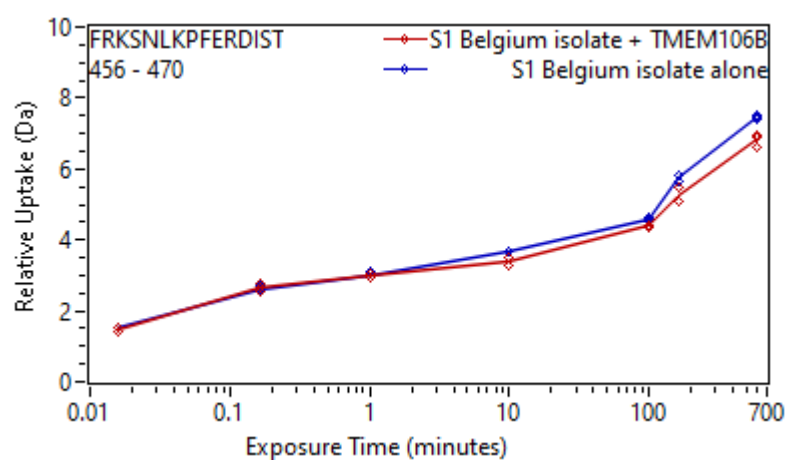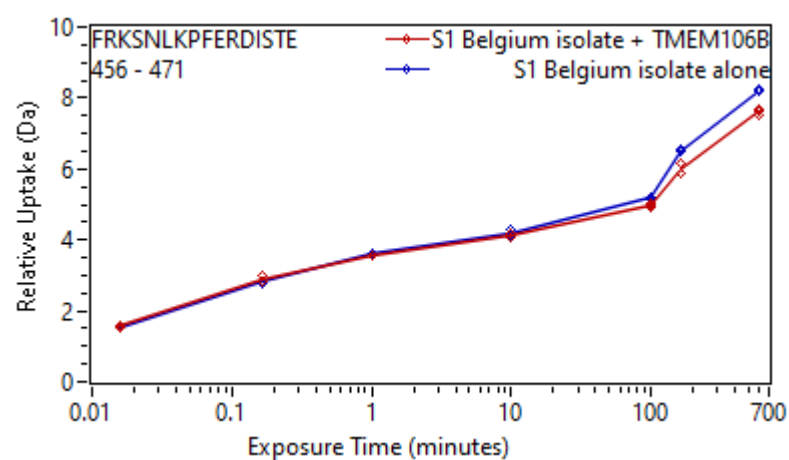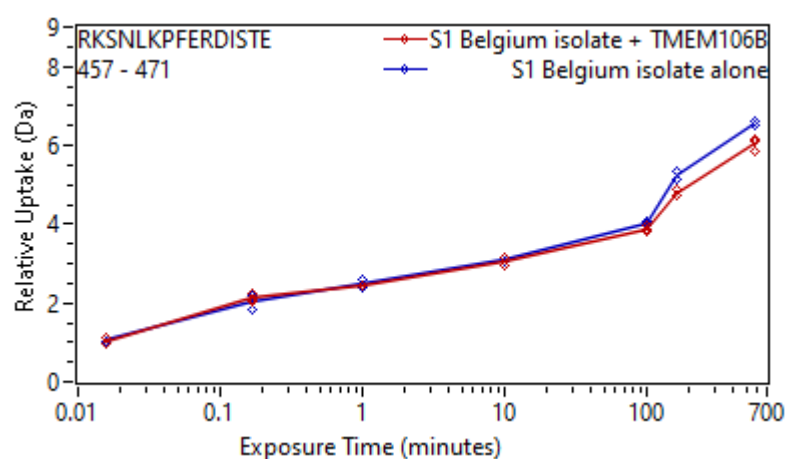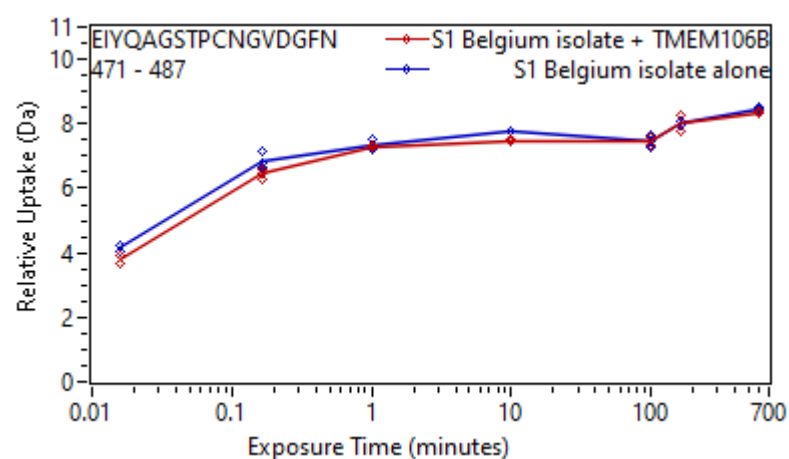

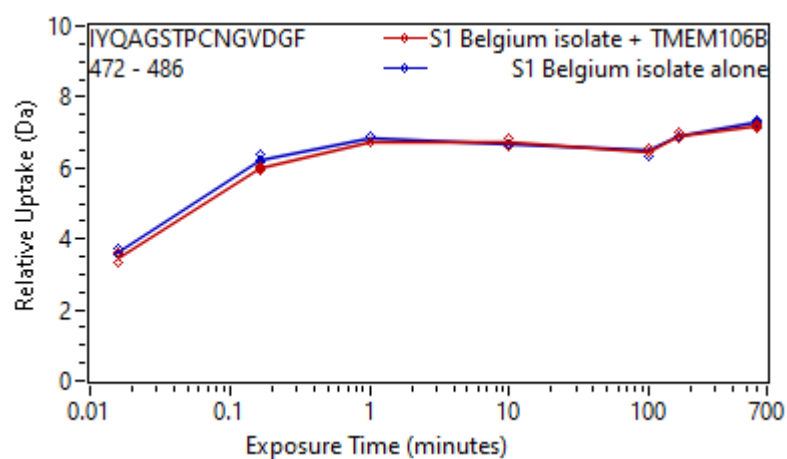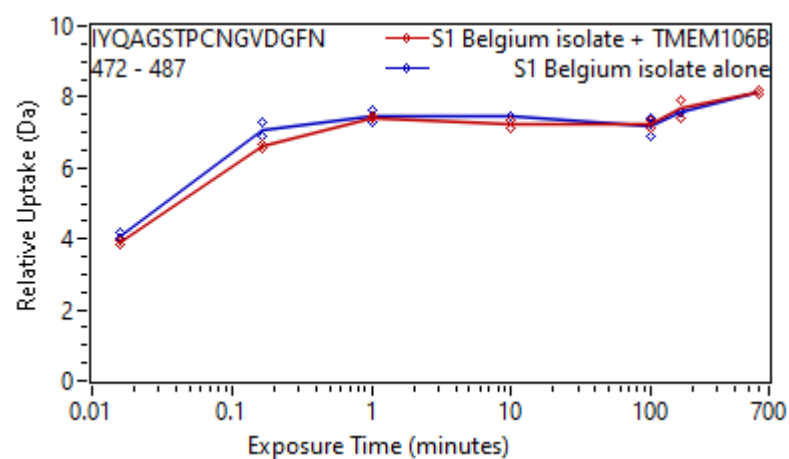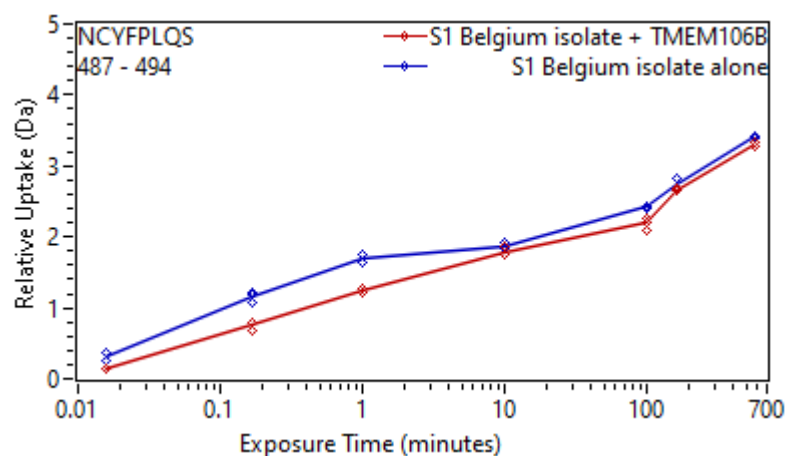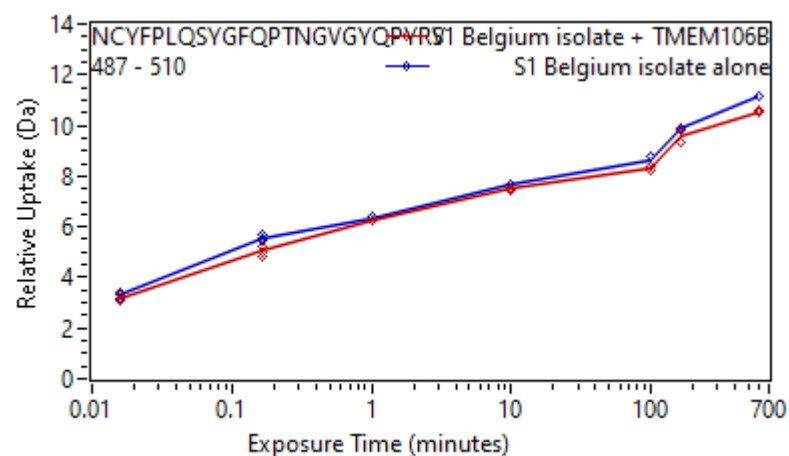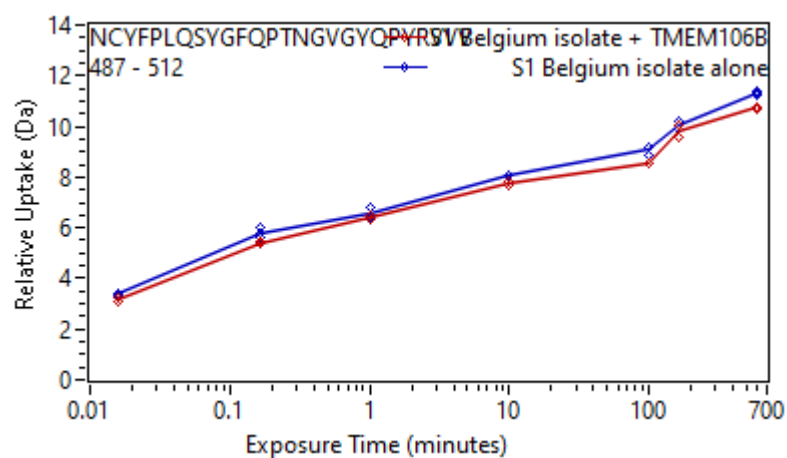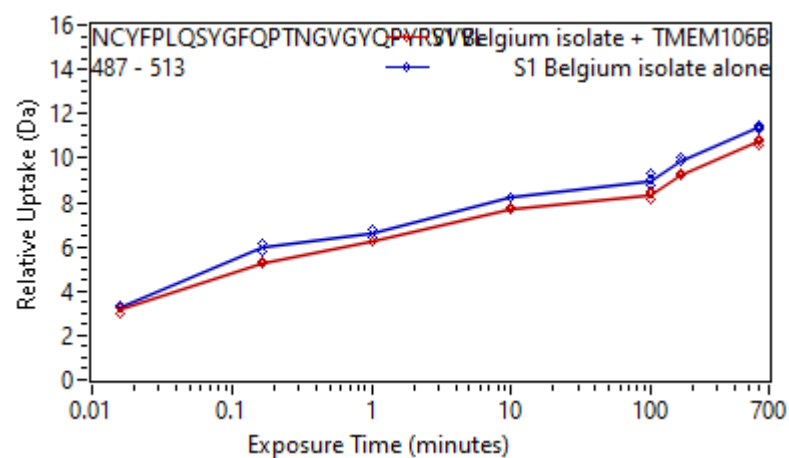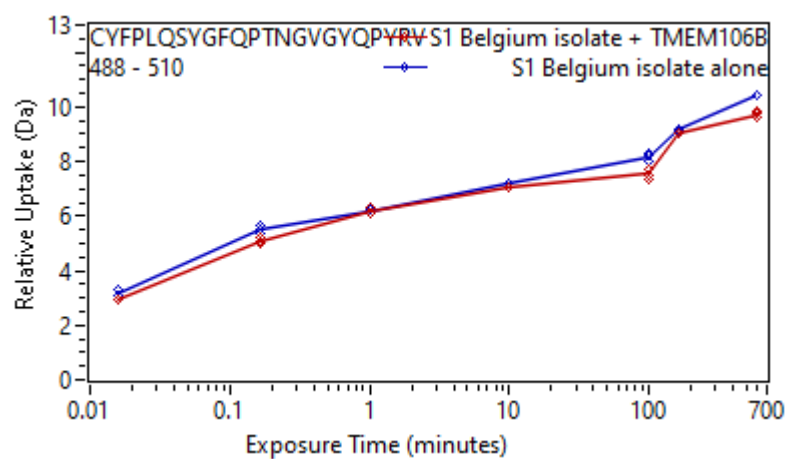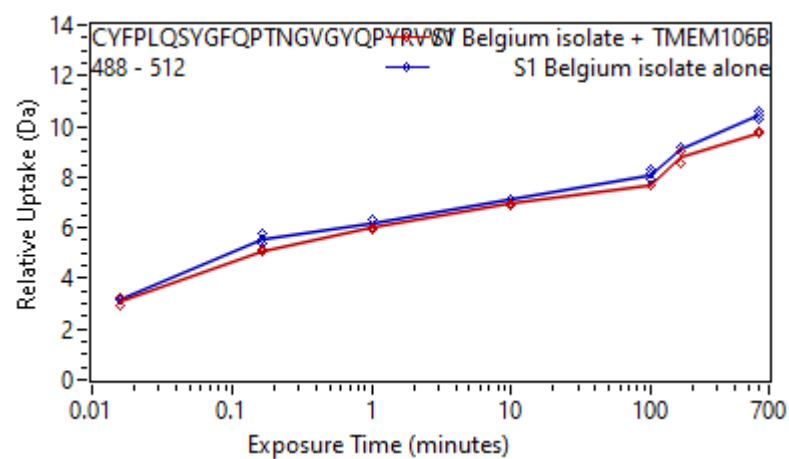

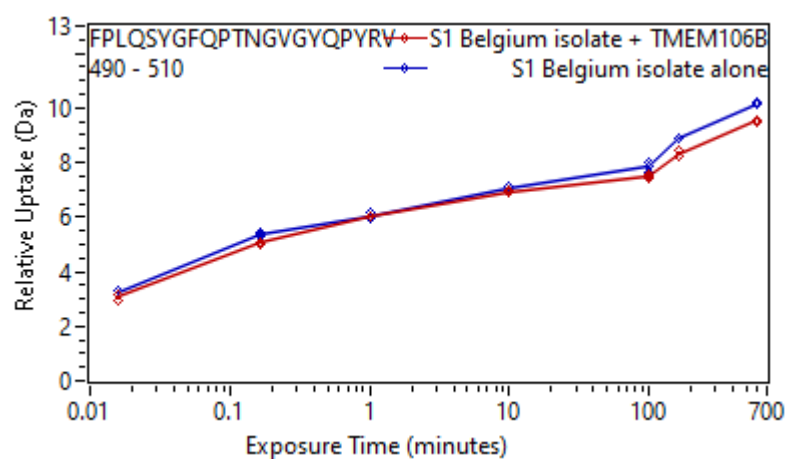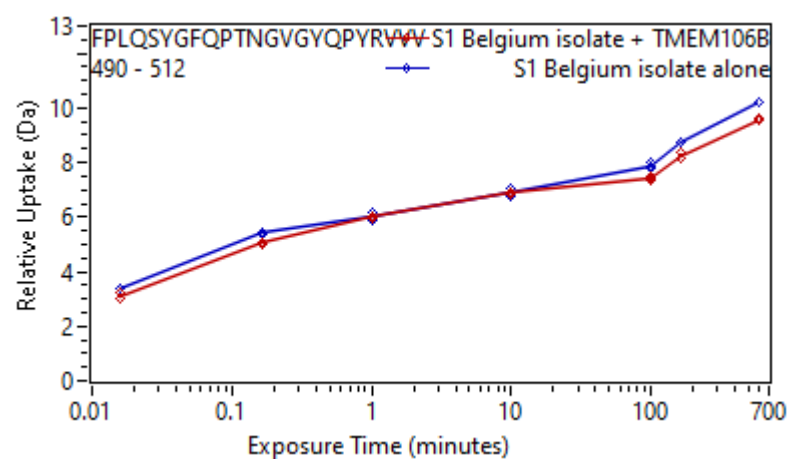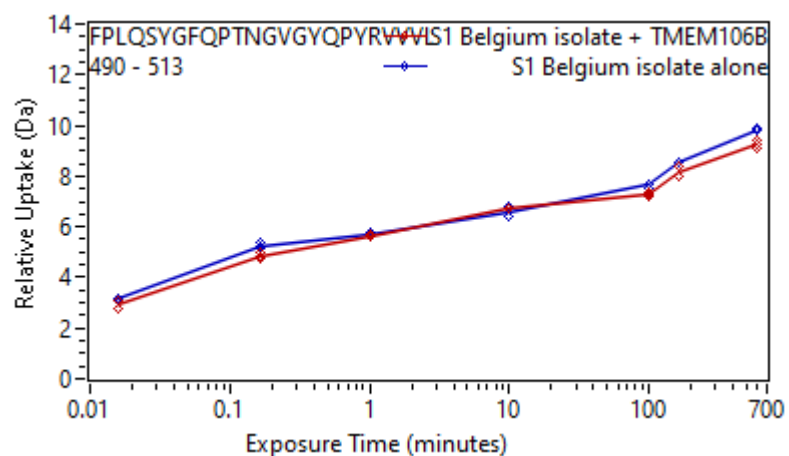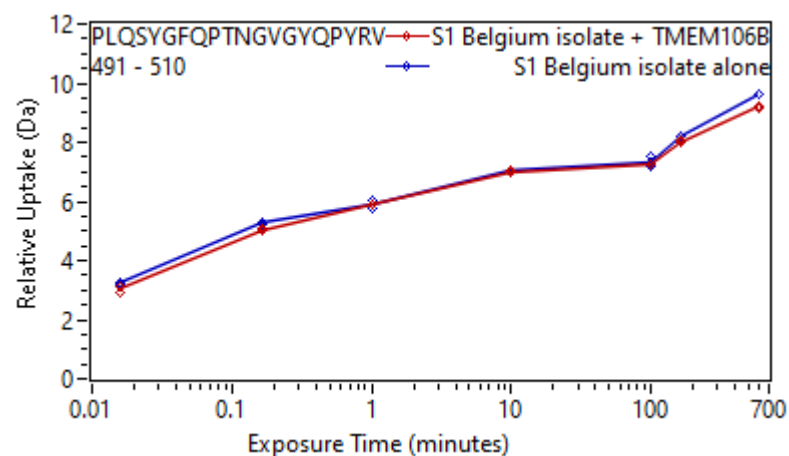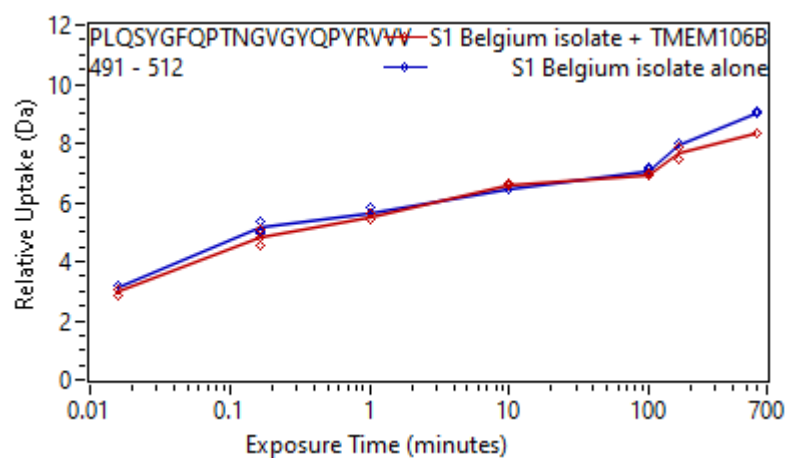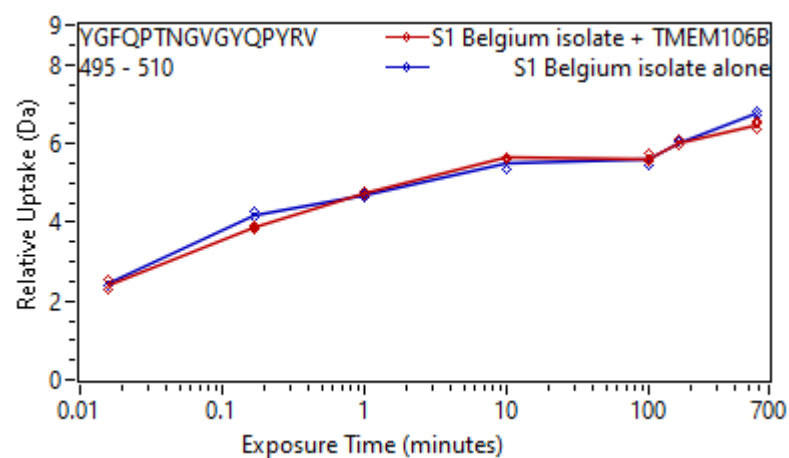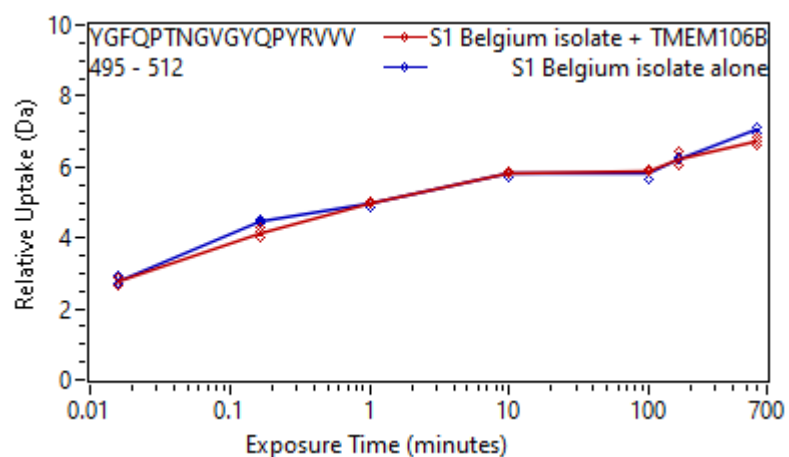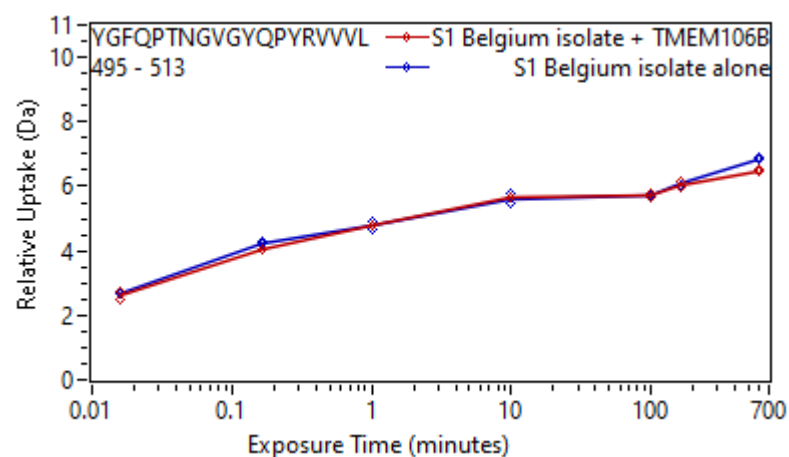

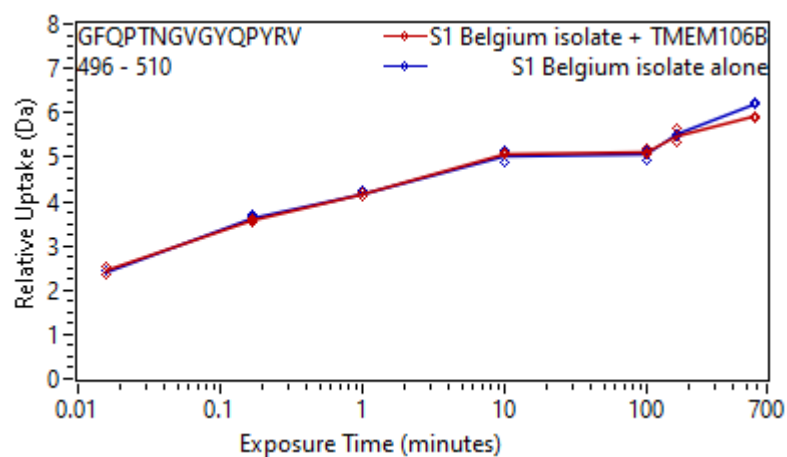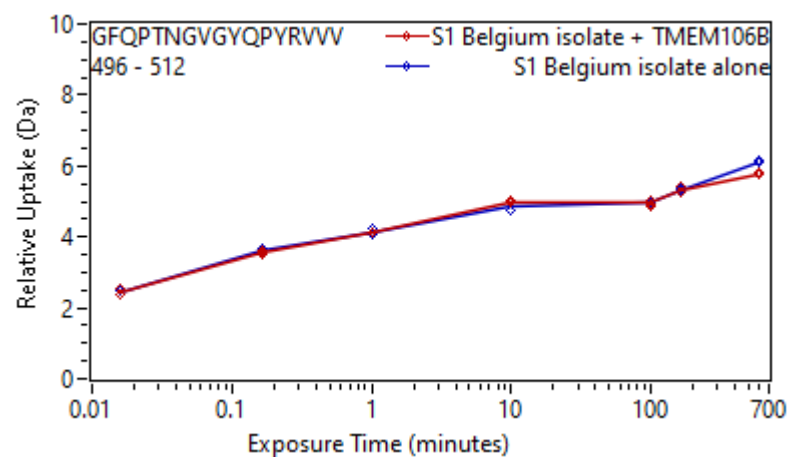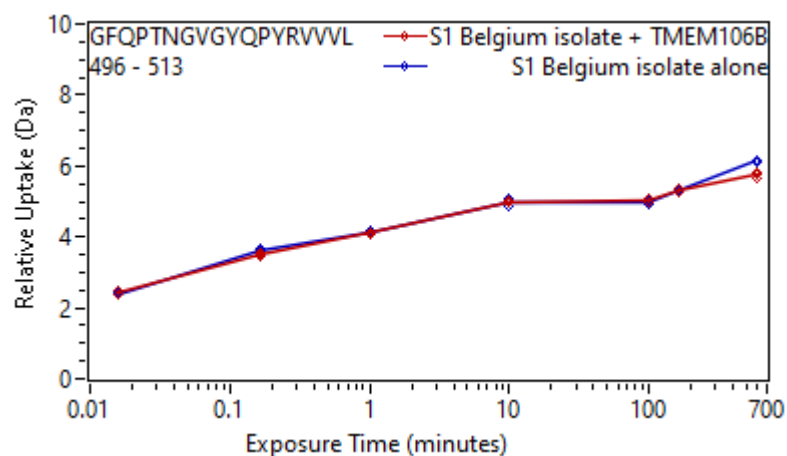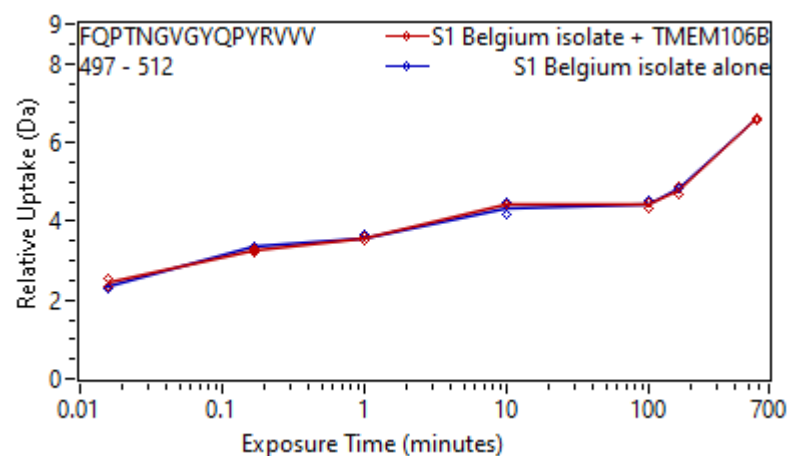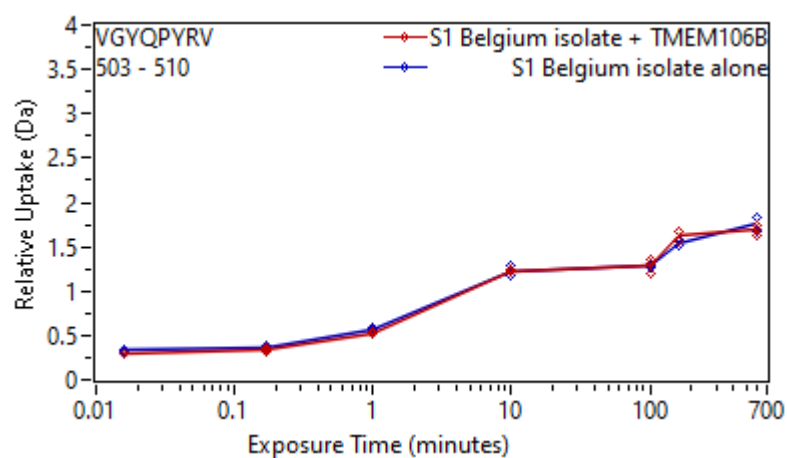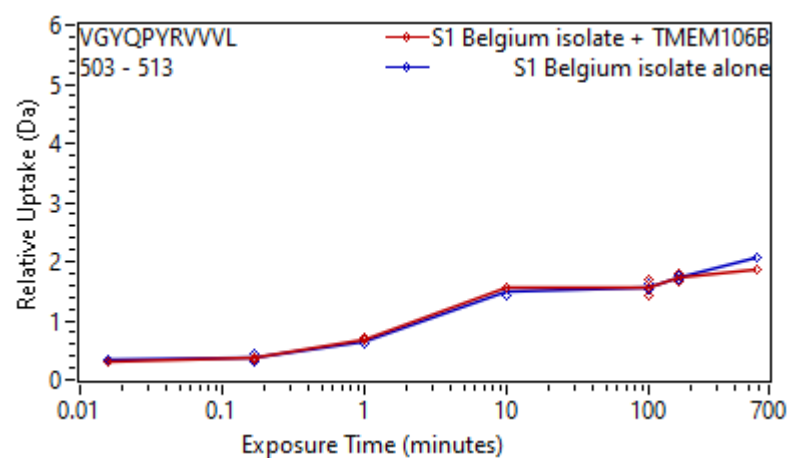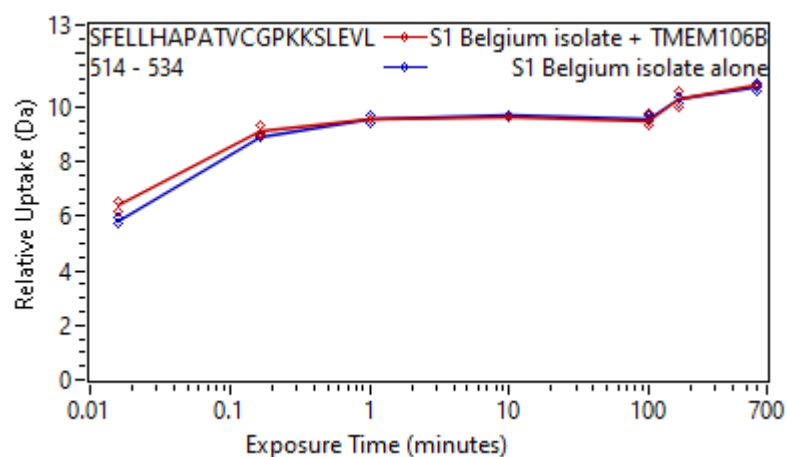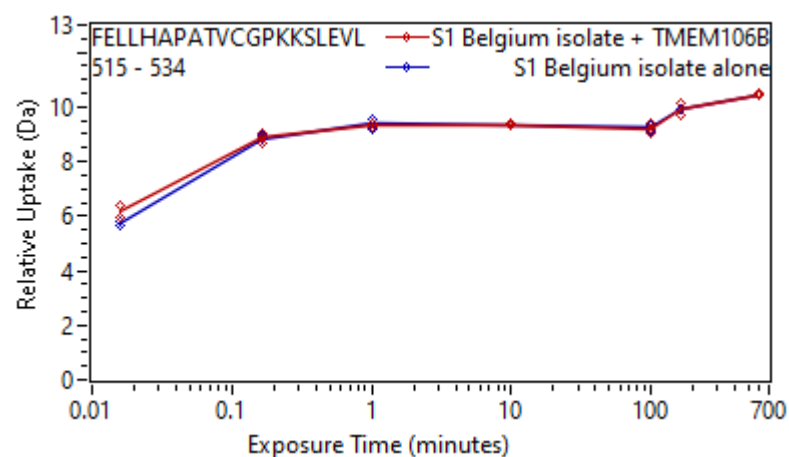

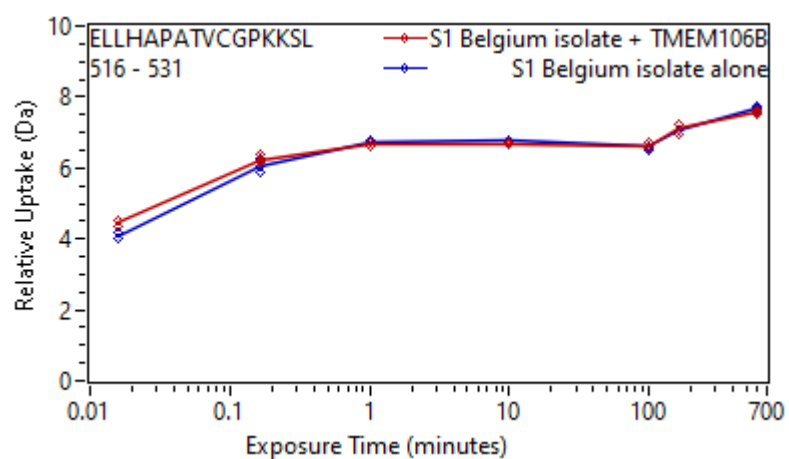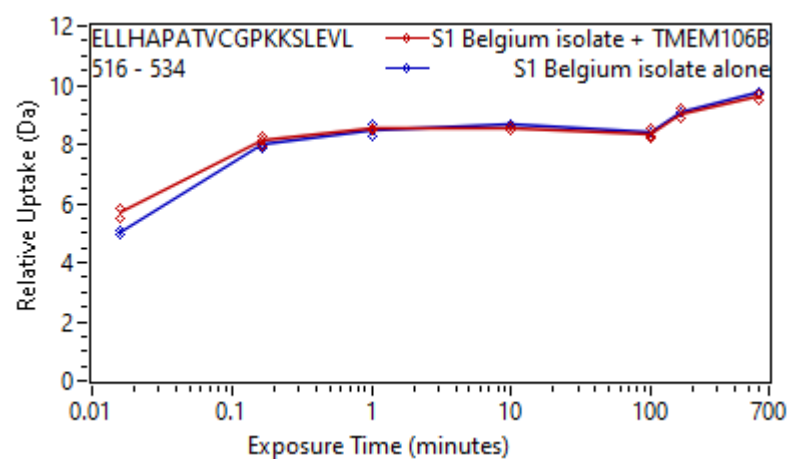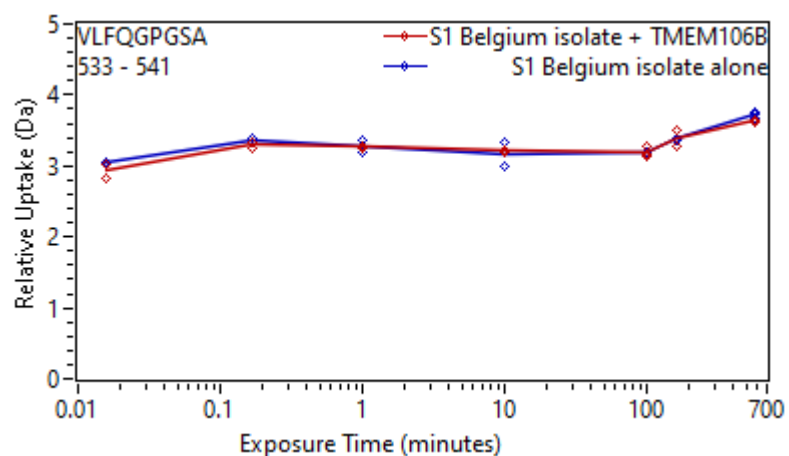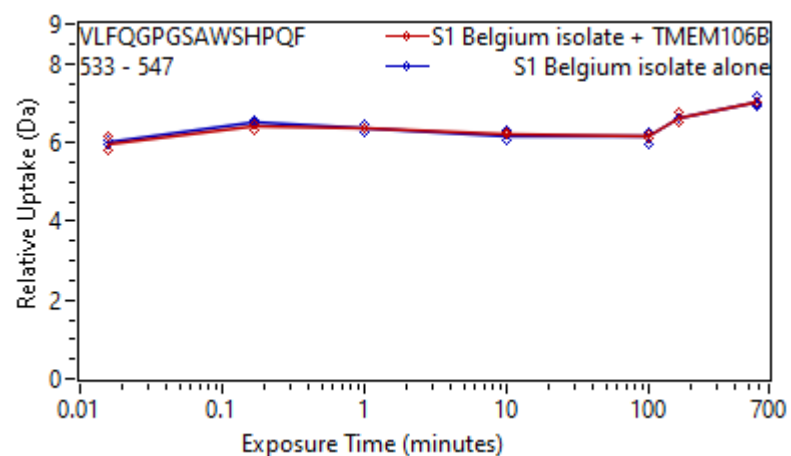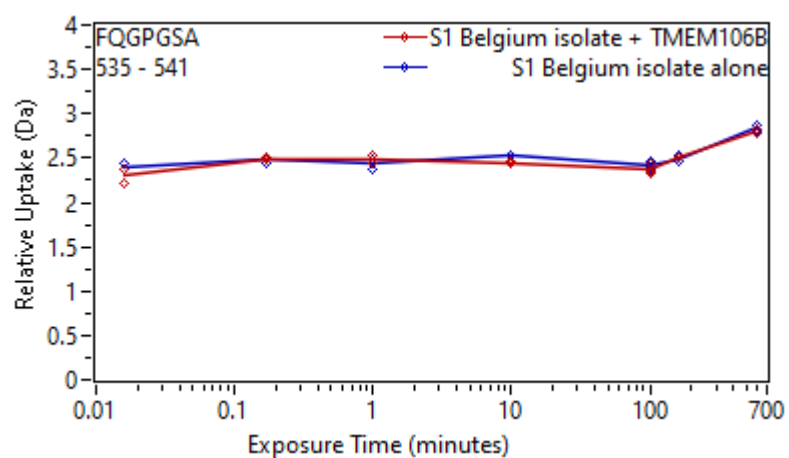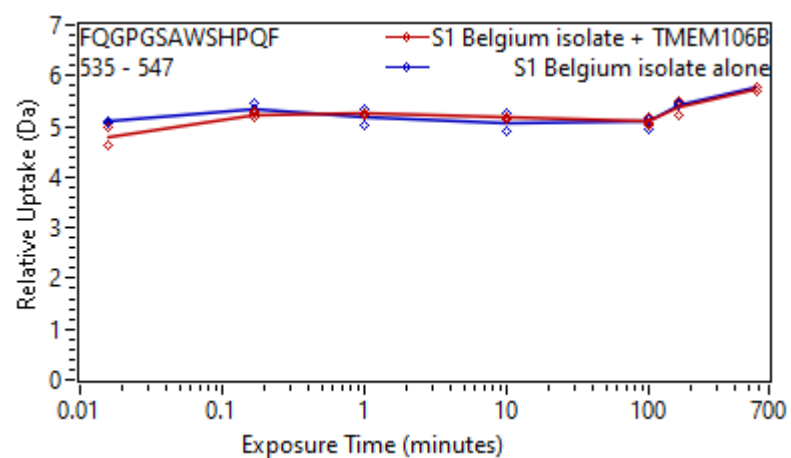

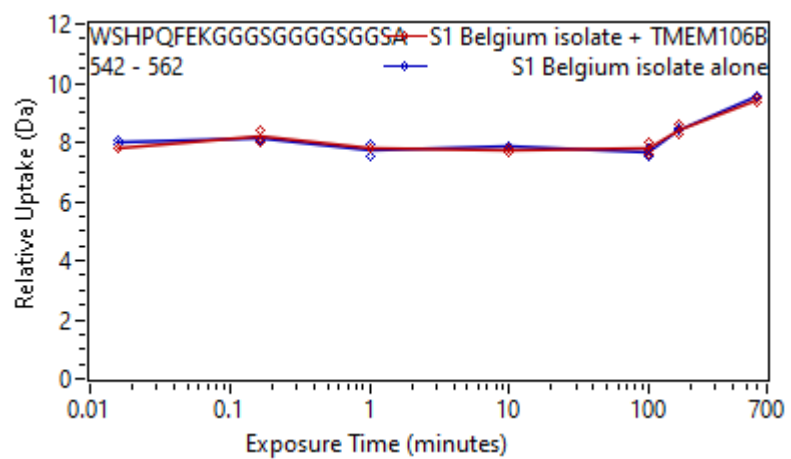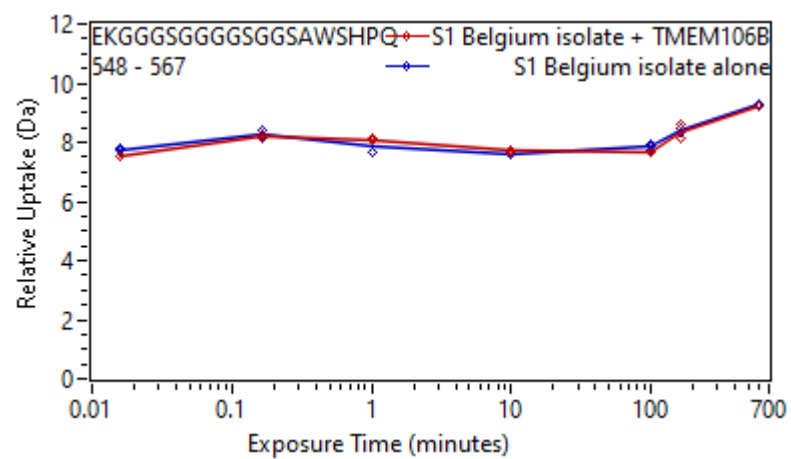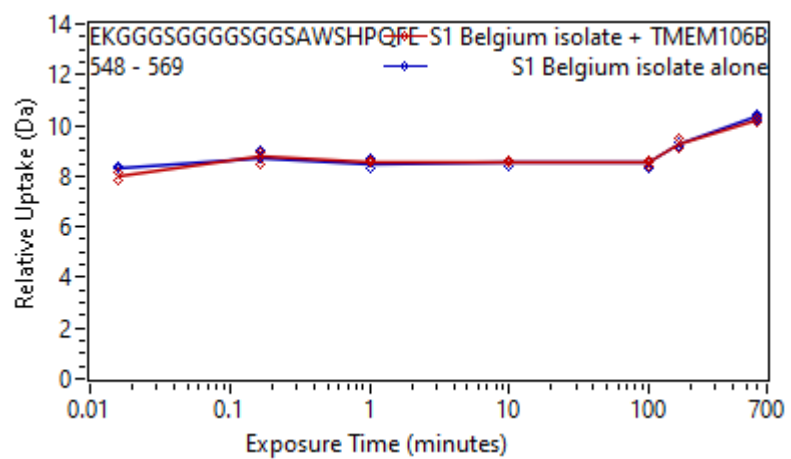

Supplement: Data S1. HDX deuterium uptake plots, related to Figure 3 [file mmc4.pdf]
